# Application of Physiologically Based Absorption Modeling to Characterize the Pharmacokinetic Profiles of Oral Extended Release Methylphenidate Products in Adults

**DOI:** 10.1371/journal.pone.0164641

**Published:** 2016-10-10

**Authors:** Xiaoxia Yang, John Duan, Jeffrey Fisher

**Affiliations:** 1 National Center for Toxicological Research, Food and Drug Administration, Jefferson, Arkansas, United States of America; 2 Center for Drug Evaluation and Research, Food and Drug Administration, Silver Spring, Maryland, United States of America; Cleveland Clinic, UNITED STATES

## Abstract

A previously presented physiologically-based pharmacokinetic model for immediate release (IR) methylphenidate (MPH) was extended to characterize the pharmacokinetic behaviors of oral extended release (ER) MPH formulations in adults for the first time. Information on the anatomy and physiology of the gastrointestinal (GI) tract, together with the biopharmaceutical properties of MPH, was integrated into the original model, with model parameters representing hepatic metabolism and intestinal non-specific loss recalibrated against in vitro and in vivo kinetic data sets with IR MPH. A Weibull function was implemented to describe the dissolution of different ER formulations. A variety of mathematical functions can be utilized to account for the engineered release/dissolution technologies to achieve better model performance. The physiological absorption model tracked well the plasma concentration profiles in adults receiving a multilayer-release MPH formulation or Metadate CD, while some degree of discrepancy was observed between predicted and observed plasma concentration profiles for Ritalin LA and Medikinet Retard. A local sensitivity analysis demonstrated that model parameters associated with the GI tract significantly influenced model predicted plasma MPH concentrations, albeit to varying degrees, suggesting the importance of better understanding the GI tract physiology, along with the intestinal non-specific loss of MPH. The model provides a quantitative tool to predict the biphasic plasma time course data for ER MPH, helping elucidate factors responsible for the diverse plasma MPH concentration profiles following oral dosing of different ER formulations.

## Introduction

Attention-deficit/hyperactivity disorder (ADHD) is one of the most common neurobehavioural disorders experienced in childhood, with estimated prevalence rates per 100,000 school-aged children ranging from 4,000–9,000 [1]. The persistence of ADHD symptoms into adolescence and adulthood has been increasingly observed in up to 50% or more of those diagnosed with ADHD in childhood. Pharmacotherapy remains the first line treatment option for ADHD, of which methylphenidate (MPH) is one of the most commonly prescribed stimulant medications. MPH works by inhibiting the presynaptic dopamine transporter [2], and the conventional, immediate release (IR) formulation of MPH became a mainstay of treatment for ADHD since 1955 when ADHD was termed Minimal Brain Dysfunction (MBD) [3]. Due to the short duration of action of IR MPH formulations, twice-daily or three-times-daily administration is required to maintain therapeutic effects during the normal school day and after-school hours. However, such a dosing schedule can be problematic due to the issues of inconvenience, security, and privacy at school, which may compromise treatment outcomes.

To address these issues, once-daily extended release (ER) MPH formulations have been designed to achive the desired continuous behavior improvements throughout the day with a single dose. Per FDA, “extended-release drug products are dosage forms that allow a reduction in dosing frequency as compared to when the drug is present in an immediate-release dosage form. These drug products can also be developed to reduce fluctuations in plasma concentrations” [4]. Various oral ER MPH formulations have been developed, such as Concerta using OROS technology (osmotic controlled-release oral system tablets comprising of 22% IR and 78% ER MPH), Metadate CD and Equasym XL using Diffucaps technology (containing 30% IR and 70% ER beads), Ritalin LA using SODAS technology (spheroidal oral drug absorption system delivering 50% IR and 50% enteric-coated ER beads), Medikinet retard (comprising 50% IR and 50% enteric coated ER pellets), as well as the newly approved Aptensio XR (using a novel multilayer release bead formulation, MLR-MPH, containing 40% IR/60% ER beads) [2, 5], and other fomulation products.

In general, many of the ER MPH formulations were designed to provide a fast onset of action, followed by a prolonged duration of action [6], except for some formulations, e.g. Metadate-ER nor Ritalin-SR. The initial rapid onset might be attributed to the “ramp” effect associated with the quick dissolution of the IR component within each of the ER formulations [7, 8]; whereas the ER component is intended to attain a lowered drug concentration at noon with a second peak in drug concentraiton occurring 6–8 hours following dosing with a gradual decline afterwards. Such an ideal formulation design ensures a regular appetite during lunch and dinner and a usual sleep pattern, while allowing for symptom control during morning and afternoon activities [6, 9]. However, the lowered drug concentration at noon is only apparent for Ritalin LA, but otherwise is not espounsed by other product labeling nor prominently seem in PK profiles of other products.

With different delivery technologies and variable ratios between the IR and ER components, these approved ER oral dosage forms of MPH exhibit diverse profiles of absorption phases and varied patterns of action over the course of the day [10, 11]. The peak plasma concentrations, the time to reach the peak concentrations, and the rate of plasma concentrations increase and decrease differ among these formulations. A potential positive correlation between plasma MPH concentrations and the extent of ADHD sympton reduction has been proposed [1, 2, 12], suggesting that the temporal clinical responses of MPH follow its plasma concentration versus time profiles. Therefore, a quantitative understanding and characterization of the diverse pharmacokinetic profiles may help inform the magnitude of clinical effects throughout the day for different formulations. Such knowledge might be valuable for clinicians in terms of optimizing treatment regimens to ensure temporal symptom controls suited to individual patient’s needs.

Physiological absorption models, with the integration of anatomy and physiology of human gastrointestinal (GI) tract as well as physicochemical properties of chemicals, have been widely utilized as a useful approach to describe oral absorption and disposition of various modified release formulations [13–15]. However, to the best of our knowledge, no such model exists for ER MPH dosage forms. In the current study, a previously developed physiologically based pharmacokinetic (PBPK) model for IR MPH [16] was modified to describe the pharmacokinetic behaviors of multiple ER MPH formulations in adult humans, where ER MPH products containing multi-particulate beads/pellets with different release characteristics were considered. Instead of describing the oral uptake with an empirical first order process as described in the original model [16], various events occurring in the GI tract were described in a mechanistic framework to account for the physiology of the GI tract, physiochemical properties of MPH, and formulation related information. This mechanistic description of the GI tract provides some insight into the diversity of the absorption profiles observed among different ER MPH formulations.

## Materials and Methods

### Key pharmacokinetic studies

Three pharmacokinetic studies in adult humans following oral administration of IR MPH [17–19] were used for the recalibration of the published PBPK model for the IR MPH formulation. Recalibration of the IR MPH model was necessary to appropriately determine model parameters describing the absorption and disposition of MPH in the GI tract, and avoid the complexity associated with various engineered release/dissolution profiles for ER formulations. The first data set is from Patrick et al. (2013), where 24 volunteers (12 men aged 25.8± 2.4 years, weight 82.2±11.1 kg and 12 women aged 26.9±4.5 years, weight 59.6±6.8 kg) were orally dosed with IR MPH at 0.3 mg/kg body weight (bw) administered as 5 and 10-mg tablets (Ritalin). Blood samples (n = 12) were collected up to 12 hours after MPH dosing and plasma samples were analyzed for *d-* and *l-*MPH concentrations [18]. In the second data set, 10 women aged 28.7±4.4 years with a body weight of 65.2±8.4 kg and 10 men aged 28.8±5.3 years with a body weight of 82.2±10.5 kg received oral IR MPH at 0.3 mg/kg bw as 5 and 10-mg tablets (Ritalin). A total of 10 blood samples were collected over a period of 10 hours for measurements of the concentrations of *d-* and *l-*MPH enantiomers [17]. In the third study by Wong et al. (1998), twenty-one male subjects aged 27.6±6.1 years with a body weight of 74.7±9.0 kg received 40 mg IR MPH tablets (four 10-mg tablets). Blood samples were collected up to 18 hours and plasma concentrations of MPH enantiomers were measured [19]. The selection of pharmacokinetic studies with the quantitation of both *d-* and *l-*MPH concentrations allows for the determination of enantiospecific model parameters to account for the distinct pharmacokinetic profiles between *d-*and *l-*MPH.

For ER formulations of MPH, adult human plasma concentration time courses for total MPH were used with MLR-MPH (multilayer-release MPH), Ritalin LA using the SODAS technology, Metadate CD using the Diffucaps technology, and Medikinet retard containing enteric coated pellets. Pharmacokinetic studies for each dosage form are briefly described below. Of note, due to the low bioavailability (1%) of *l-*MPH [20], the total concentrations reported in the following studies are essentially the same as those for the enantiospecific *d*-MPH.

For MLR-MPH, two pharmacokinetic data sets were selected for model evaluation. In the first data set, 21 young adults (11 females and 10 males) aged 19–25 years were given a single oral dose of a 20-mg capsule of MLR-MPH under a fasting state, for which 40% of the total dose is available for immediate release. A total of 24 blood samples were collected over a 24 hour period and plasma concentrations of total MPH were determined [21]. In the second data set, healthy female (n = 11) and male (n = 15) adults aged 18–45 years with a body weight of 70.4±11.7 kg received a single oral dose of a 80 mg MLR-MPH capsule containing 37% IR MPH under fasted conditions. The time course of total plasma MPH concentrations (total 24 time points) was recorded for 24 hours after dosing [6].

For Ritalin LA using the SODAS technology containing 50% IR beads and 50% delayed-release beads, three pharmacokinetic data sets were used for model evaluation. In the first study, a single oral dose of 40 mg Ritalin LA was given to 20 healthy adult male (n = 14) and female (n = 6) subjects (age, 31±7.75 years; weight, 74.3±12.42 kg) under fasting conditions. Blood samples (n = 17) were collected over a period of 24 hours to assay plasma total MPH concentrations [22]. In the second study of Markowitz et al. (2003), nineteen healthy subjects, ten male and nine female, aged 24±3 years with a body weight of 70±11.7 kg, were given a single oral dose of 20 mg Ritalin LA. Blood samples (n = 19) were obtained from each subject and plasma samples were immediately prepared for the analysis of total MPH concentrations [9]. In the third data set, male volunteers (n = 28) with a mean age of 25.8 ±2.6 years, body weight of 79.1±9.4 kg received a single oral dose of 40 mg Ritalin LA under fasting conditions. Blood samples (n = 18) were collected over a period of 24 hours and the concentrations of total MPH in plasma were determined [23].

For Metadate CD utilizing the Diffucaps technology, for which 30% of the dose is provided by IR beads and 70% is provided by ER beads, two pharmacokinetic data sets were used for model evaluation. In the first data set, healthy male (n = 14) and healthy female (n = 12) subjects, aged 21–40 years with a mean body weight of 72.4 (54.3–91.4) kg received a 20 mg Metadate CD capsule under fasted conditions. Blood samples (n = 14) were collected over a period of 24 hours and plasma total MPH concentrations were determined [24]. In the second study, 20 mg Metadate CD capsules were given to adult subjects (21 males and 14 females) under fasted conditions. The age and body weight of the subjects in the study were 29 ± 6.2 years and 71.1 ±11.8 kg. Blood samples (n = 18) were collected for 24 hours after dosing for measurements of plasma total MPH concentrations [25].

Three data sets were utilized for model evaluation of Medikinet retard, of which 50% is provided by the IR component and the other 50% is composed of enteric coated ER beads. As opposed to the other ER MPH formulations, Medikinet retard is recommended to be taken with or after breakfast to retain the medication long enough in the stomach, thus ensuring a prolonged supply of MPH over the day and resulting in a biphasic kinetic profile with increased overall exposure to MPH [23, 26]. Therefore, selected pharmacokinetic studies with Medikinet retard were performed in adult humans under fed conditions (Note that “fed” in the current manuscript refers to diet/meal in general, not particular to a high fat meal). In the first data set, volunteers (n = 28, male) with a mean age of 25.8 ±2.6 years, weight of 79.1±9.4 kg received a single oral dose of 40 mg Medikinet retard under fed conditions. Blood samples (n = 18) were collected over a period of 24 hours and concentrations of total MPH in plasma were determined [23]. In the second study, healthy volunteers (n = 14, 7 male and 7 females) aged 33±6.8 years with a body weight of 66.1±9.92 kg were given a single oral dose of 20 mg Medikinet retard under a fed state. Plasma concentrations of total MPH were measured over a period of 24 hours [27]. In the third data set, volunteers (n = 12, 8 females and 4 males) aged 35±8 years with a body weight of 71±13 kg were given a single oral dose of 20 mg Medikinet retard capsule under fed conditions, with blood samples (n = 16) obtained over a period of 24 hours for the determination of total plasma MPH concentrations [28].

### Modeling strategy

The development of the current physiological description for oral absorption of ER MPH formulations in adults was based upon the original PBPK model for IR MPH [16], with an expanded depiction of the GI tract and the release/dissolution processes of different dosage formulations. The physiological absorption model for ER MPH was first calibrated using the pharmacokinetic data sets with oral dosing of IR MPH, and then extended to describe the biphasic plasma concentration profiles of MPH for oral ER MPH formulations. Model codes describing the anatomy and physiology of the GI tract, as well as the intestinal absorption and disposition of MPH were created based on the codes contained in ADME WorkBench (the Aegis Technologies Group, Inc., Orlando, FL), the ACAT model in the Gastroplus program (Simulations Plus, Inc., Lancaster, CA), and the published literature as described below. Plasma concentration-time profiles were obtained from literature by digitizing published graphs using DigitizeIt (version 2.0.3, Braunschweig, Germany). All simulations were carried out using acslX (version 3.0.2.1, the Aegis Technologies Group, Inc., Orlando, FL) (S1 File).

### The oral absorption PBPK model

**Fig 1:**
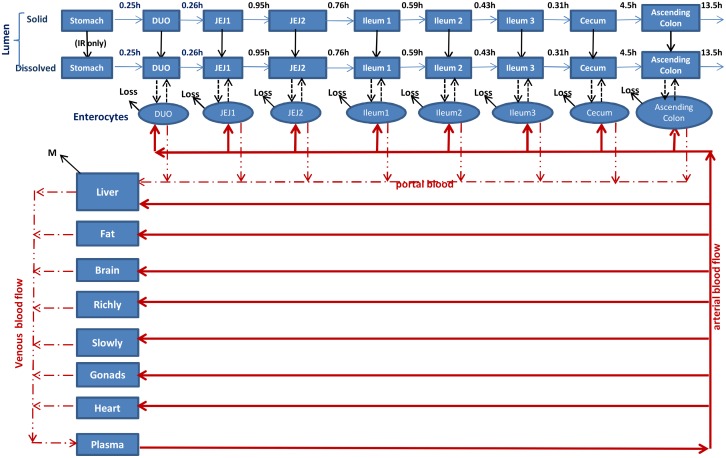
Schematic depicting the physiological absorption model for immediate release (IR) and extended release (ER) MPH formulations. The oral absorption model is composed of nine compartments, representing the stomach, duodenum, jejunum divided into two compartments, ileum divided into three compartments, cecum, and ascending colon. Dissolution of the IR component occurs throughout the GI tract, primarily in the stomach, whereas the release/dissolution of the ER component takes place only in the intestine accompanied by a delay time. M, metabolism; Loss, non-specific loss, IR immediate release.

The schematic of the whole body PBPK model incorporated with the oral absorption model is depicted in Fig 1. Model parameters for the whole body PBPK model presented in the original paper [16] were used in the current model, except that hepatic metabolic constants representing hydrolysis were derived directly from in vitro studies and model parameters describing hepatic oxidation, as well as non-specific loss in the GI tract were recalibrated as described below.

Consistent with the original model, the hydrolysis of MPH enantiomers in the liver was described with a Michaelis-Menten equation integrating the competitive binding to the hCES1A1 enzyme between *d-* and *l-*MPH [16]. The Michaelis constants for *d*- and *l*-MPH (*Kmliverd* and *Kmliverl*, μg/L) were set equal to the reported Km values, and the maximum hepatic reaction velocity (*VmaxliverdC* and *VmaxliverlC*, μg/h/kg^0.75^) were derived based on the reported in vitro catalytic constant values (*Kcat*, 0.165 min^-1^ and 0.335 min^-1^) experimentally determined using the recombinant human CES1A1 enzyme [29] as described in the original paper [16] without any adjustment. Accordingly, the clearance terms (*KmetdC* and *KmetlC*, L/h/kg^0.75^) representing the oxidation of MPH enantiomers in the liver were determined to ensure that 20% of the total dose metabolized in the liver undergoes this pathway [30].

The oral absorption model, similar to what has been proposed in the ACAT model in the Gastroplus program (Simulations Plus, Inc.), is composed of nine compartments representing the stomach, duodenum, jejunum divided into two compartments, ileum divided into three compartments, cecum, and ascending colon. Physiological characteristics (e.g. pH, volume, surface area, transit time, permeability) of each section and MPH-specific input data (e.g. solubility, permeability, logP, pKa) were either obtained from the literature, derived based on available data, or estimated using the ADMET Predictor (Simulations Plus, Inc.) (Tables 1 and 2). The IR component of MPH formulations undergoes dissolution quickly in GI fluids, primarily in the stomach, while the ER beads are assumed subject to release/dissolution in the intestine due to the engineered delayed delivery systems (Fig 1). Both solid and dissolved forms of MPH are emptied from the stomach and transit along the GI tract, which is described as a first-order process governed by transit time between each section (Table 1). The dissolved drug from both IR and ER components within the luminal fluids of the intestine undergoes passive diffusion into the enterocytes, where the MPH is either taken up into the portal vein and the liver or subjected to non-specific loss in the GI tract. The key equations are described below.

**Table 1:** Physiological model parameters for the gastrointestinal tract [31, 32].

Compartment	pH^a^	Transit Time (hr)^a^	Volume (mL)^a^	Length(cm)^a^	Radius (cm)^a^	Surface area (cm^2^)^b^	Bile Con (mM)^c^	Blood Flow (fraction of cardiac output)^d^	Mass (fraction of body weight)^e^
Stomach	1.3/4.9^f^	0.25/1^f^	50	/^g^	/^g^	/^g^	/^g^	/^g^	/^g^
Duodenum	6.0/5.4^f^	0.26	48	15	1.6	19,995	2.8/14.44^f^	0.024/0.026^h^	0.00077/0.00088^h^
Jejunum 1	6.2/5.4^f^	0.95	175	62	1.5	77,482	2.33/12.02^f^	0.024/0.026^h^	0.0019/0.0019^h^
Jejunum 2	6.4/6.0^f^	0.76	140	62	1.34	69,217	2.03/10.46^f^	0.024/0.026^h^	0.0019/0.0019^h^
Ileum 1	6.6	0.59	109	62	1.18	60,952	1.41/7.28^f^	0.024/0.026^h^	0.0014/0.0015^h^
Ileum 2	6.9	0.43	79	62	1.01	52,171	1.16/5.99^f^	0.024/0.026^h^	0.0014/0.0015^h^
Ileum 3	7.4	0.31	56	62	0.85	43,906	0.14/0.73^f^	0.024/0.026^h^	0.0014/0.0015^h^
Caecum	6.4	4.50	53	13.75	3.5	1,964	0	0.024/0.026^h^	0.0004/0.0004^h^
Ascending Colon	6.8	13.5	57	29.02	2.5	2,961	0	0.024/0.026^h^	0.00085/0.00093^h^

^a^ Set to values reported byAlmukainzi, et al (2014);

^b^ Calculated using the equations developed by Helander and Fandriks (2014) incorporating the surface enlargements due to plicae circulares, villi and microvillie in the small intestine (*A*_*i*_ = *Length*_*i*_×*Diameter*_*i*_×*π*×1.57×6.5×13) and the large intestine (*A*_*i*_ = *Length*_*i*_×*Diameter*_*i*_×*π*×1×1×6.5)

^c^ Values obtained from the Gastroplus program (Simulations Plus, Inc. Lancaster, CA);

^d^ Assuming the blood flow to the GI tract equals the portal vein blood flow and is evenly distributed among each section; portal vein blood flow is calculated as total blood flow to the liver minus blood flow to hepatic artery;

^e^ Mass for each section of jejunum as well as mass for each section of ileum is assumed to be the same and calculated based on the reported mass for jejunum and ileum respectively by Annals of the ICRP (2003); mass for cecum and ascending colon is caculated based on reported total mass of cecum and ascending colon by Annals of the ICRP (2003) to account for the ratio of the length of these two sections;

^f^ Fasting/fed, values obtained from the Gastroplus program (Simulations Plus, Inc. Lancaster, CA);

^g^ No oral uptake is considerred to occur in the stomach, therefore the associated model parameters for the stomach are not used and listed in the Table;

^h^ Male/female.

**Table 2:** Physiochemical and biochemical model parameters for IR and ER MPH.

	IR-MPH	MLR-MPH	Ritalin LA	Metadate CD	Medikinet Retard	References
**Physiochemical properties**						
Molecular weight	233.3062	233.3062	233.3062	233.3062	233.3062	[33]
Oral dose (mg)	20, 40	20, 80	20, 40	20	20, 40	Study specific
*Pka* (Base)	8.77	8.77	8.77	8.77	8.77	[34]
Exp. *LogP* (Oct/Water)	0.20	0.20	0.20	0.20	0.20	[34]
*P*_*app*_ (×10^−6^ cm/s) in MDCK	24.7	24.7	24.7	24.7	24.7	[35]
Human *Peff* (×10^−4^ cm/s)	0.963	0.963	0.963	0.963	0.963	Drived based on P_app_ and Optimization
Aqueous solubility (mg/L) at pH 10.58	9250	9250	9250	9250	9250	ADMET Predictor (Simulations Plus, Inc)
Partition Coefficient in the GI tract	5.66	5.66	5.66	5.66	5.66	Set to the value of the liver [16]
**Biochemical properties**						
Non-specific loss in the GI tract						
*K5dC* (1/h/kg^0.75^)	0.79	0.79	0.79	0.79	0.79	Optimization
*K5lC* (1/h/kg^0.75^)	37.8	37.8	37.8	37.8	37.8	Optimization
Hepatic hydrolysis						
*Kmliverd* (μg/L)	27,600	27,600	27,600	27,600	27,600	[29]
*Kmliverl* (μg/L)	10,172	10,172	10,172	10,172	10,172	[29]
*VmaxliverdC* (μg/h/kg^0.75^)	25,826	25,826	25,826	25,826	25,826	[29]
*VmaxliverlC* (μg/h/kg^0.75^)	52,404	52,404	52,404	52,404	52,404	[29]
Hepatic oxidation						
*KmetdC* (L/h/kg^0.75^)	0.43	0.43	0.43	0.43	0.43	[30]
*KmetlC* (L/h/kg^0.75^)	0.43	0.43	0.43	0.43	0.43	[30]
**Weibull function**						
*Tlag* (h)	/	4	4	4	4	Visual Fit
*A* (h^b^)	/	6	0.5	0.5	0.5	Visual Fit
*b*	/	4	2	2	2	Visual Fit

#### Release/dissolution

The rate of dissolution of IR MPH formulation in the luminal fluid (*dAdiss*_*i*_*/dt*, μg/h) was accounted for using the diffusion layer model based on the Noyes-Whitney equation for spherical particles [36] with parameters set to default values [37].

$$\frac{dAdiss_{i}}{dt}=\frac{3\times Diffcoeff}{rho\times rparticle\times DLT}\times Vlumen_{i}\times Csolid_{i}\times (Solif_{i}-Cdiss_{i})\times 60\times 100$$ (1)

where *Adiss*_i_ (μg) is the dissolved amount of IR MPH in the luminal fluid of the ith compartment at time *t*, *Diffcoeff* is the diffusion coefficient set to 1×10^−4^ cm^2^/min, rho is the density of drug particle set to 1 g/mL, *rparticle* is the current spherical radius set to 5 μm, *DLT* is the diffusion layer thickness set to 30 μm, *Vlumen*_*i*_ is the volume (mL) of the lumen of the ith compartment *Csolid*_*i*_ is the concentration (μg/mL) of undissolved IR MPH in the luminal fluid of the ith compartment, *Solif*_*i*_ is the solubility (mg/L) of MPH in the luminal fluid of the ith compartment, and *Cdiss*_*i*_ is the concentration (μg/mL) of dissolved MPH in the luminal fluid of the ith compartment.

The dissolution of IR beads/pellets within the ER MPH formulations was described as that for IR MPH formulations, whereas the release/dissolution of the ER component was described using a general empirical Weibull function:

$$\text{Re}lease=Max\times [1-\text{exp}(\frac{-{\left(t-T_{lag}\right)}^{b}}{A})]$$ (2)

where *Release* (μg) is the amount of MPH released/dissolved from ER beads/pellets at time *t*, *Max* (μg) is the amount of MPH embedded in ER beads/pellets available for release at time *t*, *A* is the scale parameter which delineates the time scale of the process, the shape parameter *b* defines the shape of the curve (b = 1 exponential; b>1 sigmoid; and b < 1 parabolic), and the lag time, *T*_*lag*_, represents the time (h) prior to the start of drug release. The value of *T*_*lag*_ was set to 4 hours for different ER formulations, which was estimated by visual fitting to achieve agreement with the onset of the second peak of plasma MPH concentration time courses for Ritalin LA and substantially ascending plasma concentration profiles across all the other data sets. The values of A and b were set to describe in vivo dissolution versus time profiles that best fit the time courses of plasma concentrations for MPH following oral dosing of ER MPH in adult subjects.

#### Solubility

In addition to the dissolution kinetics of IR and ER components of ER MPH formulations, the solubility of MPH in the luminal fluid along the GI tract was described using the following equation with the incorporation of local pH, ionization, and bile salt concentrations [38]:

$$Solif_{i}=Solwater_{i}+SolBile_{i}$$ (3)

where *Solif*_*i*_ (mg/L) is the solubility of MPH in the luminal fluid of the ith compartment, accounting for the impact of ionization at local luminal pH (*Solwater*_*i*_) and effect of bile salt concentrations on MPH solubility (*SolBile*_*i*_).

*Solwater*_*i*_ (mg/L) is the solubility of MPH in the luminal fluid of the ith compartment in the absence of bile salt, which was derived based on the Henderson-Hasselbalch equation for a monoprotic weak base [39] using the following equation:

$$LogSolwater_{i}=LogS_{0}+Log(10^{pK_{a}}{}^{-pH}+1)$$ (4)

where *S*_*0*_ is the intrinsic solubility (9,109 mg/L) calculated based on the estimated solubility of 9,250 mg/L at pH 10.58 using the ADMET Predictor (Simulations Plus, Inc.), *pH* is the *pH* value of the luminal fluid of the ith compartment and the reported *pKa* for MPH is 8.77 [34] (Table 1).

*SolBile*_*i*_ (mg/L) represents the effect of bile salt on the solubility of MPH in the luminal fluid of the ith compartment [38]

$$SolBile_{i}=SR\times SCaq\times MW\times Bile_{i}$$ (5)

where *MW* (233.31 g/mol) is the molecular weight of MPH, *Bile*_*i*_ is the concentration of bile in the ith compartment (mM), which is obtained from the Gastroplus program (Simulation Plus, Inc.) (Table 1). *SR* is the ratio of the solubilization capacity of the bile salt to the solubilization capacity of water, calculated as *SR* = 10^0.606×*LogP*+2.234^ where the experimentally determined *LogP* of MPH is 0.20 [34], and *SCaq* is aqueous solubilization capacity, calculated as *SCaq* = *Solwater*_*i*_×(*MW*_*water*_/*MW*)×10^−6^.

#### Absorption

The rate ($\frac{ddiff}{dt}$, μg/h) of absorption of dissolved MPH across intestinal mucosa is described as a passive diffusion process, since no information on the active transport of MPH could be located:

$$\frac{ddiff}{dt}=60\times 60\times HPeff_{est}\times ESA_{i}\times NI_{i}\times (Cdiss_{i}-Cmem_{i}/1000)$$ (6)

where *HPeff*_*est*_ is the estimated human effective permeability (cm/s) derived based on the relationship established between in vitro apparent permeability across Madin-Darby canine kidney (MDCK)-low efflux cells (*P*_app_) and in situ human effective permeability (*LogHPeff*_*est*_ = 0.86+0.92×*LogP*_*app*_) [40]. In vitro apparent permeability (*P*_app_) experimentally determined using the transwell assay with MDCK cells was 24.7 × 10^−6^ cm/s for MPH [35].

*ESA*_*i*_ (cm^2^) is the estimated surface area for the ith compartment, which is calculated using the equations developed by Helander and Fandriks (2014) incorporating the surface enlargements due to plicae circulares, villi and microvillie in the small intestine (*A*_*i*_ = *Length*_*i*_×*Diameter*_*i*_×*π*×1.57×6.5×13) and large intestine (*A*_*i*_ = *Length*_*i*_×*Diameter*_*i*_×*π*×1×1×6.5) [41], for which the length and diameter for each compartment was obtained from [31] (Table 1); *NI*_*i*_ is the unionized fraction of MPH in the ith compartment; *Cdiss*_*i*_ (μg/mL) is the concentration of dissolved MPH in the luminal fluid of the ith compartment and *Cmem*_*i*_ (μg/L) is the concentration of MPH within enterocytes for the ith compartment, for which the mass (volume, L) of the wall for each compartment is determined based on the ICRP Publication 89 [32] (Table 1).

#### Systemic uptake and non-specific loss in the GI tract

The uptake of MPH into the portal blood is described as a flow-limited process, where fractional blood flow to the GI tract is estimated as the difference between fractional total liver blood flow (0.255 of cardiac output for males and 0.27 of cardiac output for females) and fractional arterial blood flow to the liver (0.065 of cardiac output for both males and females) [32]. Fractional blood flow to each section of the intestine is assumed to be equal and calculated as fractional blood flow to the GI tract divided by 8, yielding a value of 0.024 of cardiac output for males and 0.026 of cardiac output for females.

With the calibration of model parameters describing hepatic hydrolysis and oxidation, non-specific loss of MPH in the GI tract was introduced to achieve agreement with observed plasma concentration profiles of MPH following oral dosing of IR MPH [17–19], which was described as a first-order process (*K5dC* and *K5lC*, 1/h/kg^0.75^). The values of *K5dC* and *K5lC*, along with the derived human effective permeability (*HPeff*_*est*_) were determined by optimization against time courses of plasma concentrations of *d*- and *l*-MPH following oral dosing of IR MPH in adult humans [17–19].

### Population simulations

Monte Carlo simulations were implemented to evaluate the population variability of the time course of plasma MPH concentrations following oral administration of IR and ER MPH formulations. Consistent with previous studies [42–45], a normal distribution was assumed for parameters representing blood flows and tissue volumes, while model parameters describing cardiac output, partition coefficients, and chemical specific model parameters, as well as those associated with the GI tract were assumed to be log-normally distributed [46, 47] (S1 Table). The coefficients of variation (CV) for cardiac output and partition coefficients were assumed to be 9% and 20%, while a CV of 30% was assumed for the rest model parameters, except for those representing the anatomy and physiology of the GI tract, for which a CV of 10% was assumed unless otherwise noted [46, 47]. To ensure physiological plausibility, the upper and lower bounds of the distribution were truncated at 1.96 times the standard deviation (SD) above and below the mean values [45]. The distribution of body weight was set based on information, if available, in each specific study. To maintain blood and mass balance, the randomly designated physiological parameters were adjusted in a fractional manner [45, 48]. The model was run 1000 times for each PK study used for model simulation with model parameters randomly sampled from the defined distributions, and the mean and 90% confidence interval of simulated plasma concentration curves were calculated.

### Assessment of model performance

In all the data sets used for model development, the mean values and standard deviation (SD) or error of the observed concentrations were reported instead of individual data points. Therefore, the accuracy of model predictions was graphically assessed by superimposing the predicted and observed mean plasma concentration-time profiles [49]. Predictions that were within a factor of two of the experimental data were considered adequate as proposed by the World Health Organization [50].

The ability of the current model to predict the pharmacokinetic parameters, e.g. the area under the plasma concentration-time curve from time 0 to the last measurable concentration point (*AUC*_*last*_), the area under the plasma concentration-time curve from time t1 to time t2 as specified in each data set (*AUC*_*t1-t2*_), the maximal concentration (*C*_*max*_), and the time (T_*max*_) to reach the *C*_*max*,_ was also examined. For this analysis, the mean and SD of the predicted pharmacokinetic parameters from 1000 population simulations for each study were evaluated and compared with the respective reported values in literature. Predictions of pharmacokinetic parameters were considered successful if the fold error, i.e. difference between predicted and observed mean values, was less than a factor of two [49, 51–55].

### Parameter sensitivity analysis

A local sensitivity analysis was performed using the built-in functionality of acsIX to evaluate influence of model parameter perturbations on model predicted time courses of plasma total MPH concentrations for different MPH formulations. The normalized sensitivity coefficient (NSC) was calculated by the following equation [56]:

$$NSC=\frac{(O_{i}-O)/O}{(P_{i}-P)/P}$$ (7)

where *O* is the model output associated with the original parameter value, *O*_*i*_ is the model output resulting from the 1% increase in the parameter value, *P* is the original parameter value, and *P*_*i*_ is the parameter value increased by 1%. Model parameters with maximum absolute NSC values greater than 0.1 were considered to be sensitive; whereas those with maximum absolute NSC values exceeding 1 had a high impact on model output. Additionally, a direct correlation between the model output and the corresponding parameter is suggested by a positive NSC, while a negative NSC indicates the model output is inversely associated with the specific parameter.

## Results

### Model Calibration for IR MPH

With the Michaelis affinity constants (*Kmliverd* and *Kmliverl*) and the maximum hepatic reaction velocities (*VmaxliverdC* and *VmaxliverlC*) describing *d*- and *l*-MPH hydrolysis in the liver set directly to the in vitro derived values (Table 2), clearance terms representing hepatic oxidation of MPH enantiomers, *KmetdC* and *KmetlC*, were set to a value of 0.43 L/h/kg^0.75^, yielding 20% of the total MPH metabolized in the liver undergoing the oxidation pathway [30]. The optimized gut non-specific loss constants (*K5dC* and *K5lC*) and effective human permeability (*HPeff*_*est*_) were 0.79 1/h/kg^0.75^, 37.8 1/h/kg^0.75^, and 9.63×10^−5^ cm/s, respectively (Table 2).

**Fig 2:**
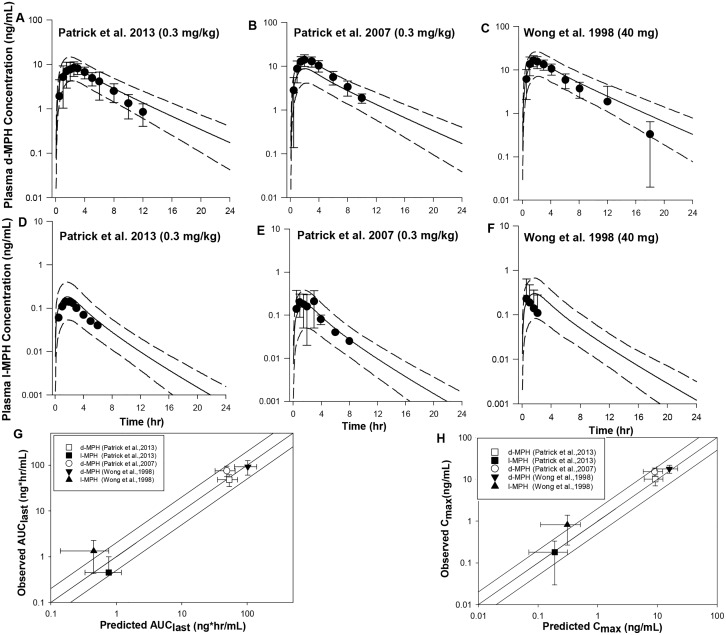
Model simulated versus observed *d*- MPH (A, B, C) and *l*-MPH (D, E, F) plasma concentration profiles in adults receiving a single oral dose of IR MPH at 0.3 μg/kg [17, 18] and 40 mg [19] under fasted conditions as well as model predictability of the pharmacokinetic parameters *AUC* (G) and *C*_*max*_ (H). Solid lines in A-F represent simulated mean plasma concentration-time profiles, whereas dashed lines represent the 5^th^ and 95^th^ percentiles for the predicted values. The observed data points (●) are shown as mean ± SD (error bars) or means only. The solid lines in G and H illustrate unity as well as a twofold deviation from unity; whereas the data points represent observed mean ± SD values with respective to the simulated mean ± SD values for *AUC* (G) and *C*_*max*_ (H). In the study of Patrick, Straughn et al. (2007), *AUC* and *C*_*max*_ values only reported for *d*-MPH.

Model predicted mean plasma *d*- and *l*-MPH concentration time courses were in good agreement with observations across all the data sets, with predicted mean concentrations within a factor of two of the observed values [17–19] (Fig 2A–2F), except that the mean concentrations for *d*-MPH at 18 h and *l-*MPH at 1.5 and 2 h were slightly overestimated in subjects receiving 40 mg IR MPH [19] by a factor of 2.1–2.7 (Fig 2C and 2F). The model-predicted mean pharmacokinetic model parameters *C*_*max*_ and *AUC*_*last*_ were consistently within twofold of the experimental values (Fig 2G and 2H) for all three data sets, except that the estimated mean *AUC*_*last*_ and *C*_*max*_ values were somewhat lower compared with observations for *l*-MPH in one study [19] (S2 Table). Of note, the reported mean *C*_*max*_ and *AUC*_*last*_ values in Table II for *l*-MPH for the study of [19] were not consistent with those shown in Figure 2 of the same paper [19]. The recalculated mean *C*_*max*_ and *AUC*_*last*_ values derived by digitalized concentration time course data from Fig 2 [19] were in good agreement with model predictions (S2 Table). In addition, the model-predicted *T*_*max*_ for *d*- and *l*- MPH in general agreed with observations for all three data sets except for *l*-MPH for the study of [19], for which the predicted median time to reach peak plasma *l*-MPH concentration was delayed compared with experimental data by more than 2 fold (S2 Table).

### Model Evaluation for ER MPH

**Fig 3:**
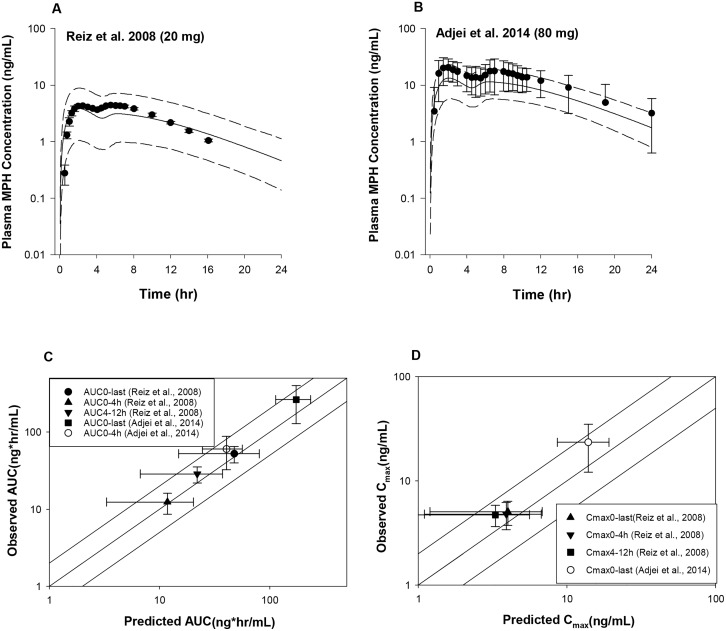
Model simulated versus observed total MPH plasma concentration profiles in adults receiving a single oral dose of MLR-MPH at (A) 20 mg [21] and (B) 80 mg [76] under fasting conditions, as well as model predictability of the pharmacokinetic parameters *AUC* (C) and *C*_*max*_ (D). Solid lines in A and B represent simulated mean plasma concentration-time profiles, whereas dashed lines represent the 5^th^ and 95^th^ percentiles for the predicted values. The observed data points (●) are shown as mean ± SD (error bars) for the study of Adjei et al (2014), while for Reiz et al. (2008), the observed data points are expressed as mean ± SE. The solid lines in C and D illustrates unity, as well as a twofold deviation from unity; whereas the data points represent observed mean ± SD values with respective to the simulated mean ± SD values for *AUC* (C) and *C*_*max*_ (D).

To fit the extended release MPH formulations, parameters of the Weibull equation (Eq 2) were adjusted except for the lag time, which was set to 4 hr. For MLR-MPH, a value of 6 (hr^b^) for *A* and a value of 4 (unitless) for *b* (Table 2) were visually fitted to achieve better agreement with observed plasma MPH concentration-time profiles in adults received 20 mg [21] and 80 mg [6] MLR-MPH capsule under fasting conditions. As shown in Fig 3A and 3B, model-simulated mean plasma MPH concentration time profiles were in good agreement with observations for both data sets, except for the 0.5 h time point in the study of [21] where observed mean plasma total MPH concentration was overestimated by 5-fold.

The adequate description of experimental mean plasma profiles in subjects receiving MLR-MPH was further supported by the good estimation of pharmacokinetic parameters. Model predicted *AUC* and *C*_*max*_ values all met the evaluation criterion for both studies, with predicted/observed mean values in the range of 0.6–0.95 (Fig 3C and 3D and S3 Table). Also, the observed *T*_*max*_ values were tracked very well by the current model (S3 Table).

**Fig 4:**
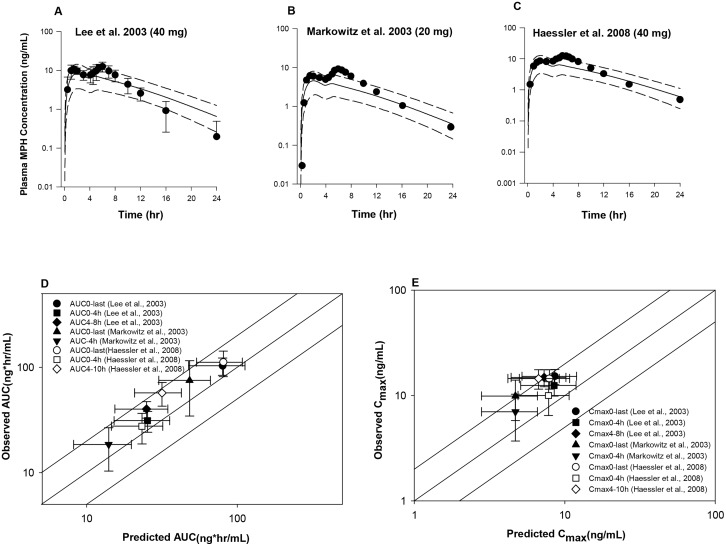
Model simulated versus observed total MPH plasma concentration profiles in adults receiving a single oral dose of Ritalin LA at (A, C) 40 mg [22, 23] and (B) 20 mg [9] under fasting conditions, as well as model predictability of the pharmacokinetic parameters *AUC* (D) and *C*_*max*_ (E). Solid lines in A-C represent simulated mean plasma concentration-time profiles, whereas dashed lines represent the 5^th^ and 95^th^ percentiles for the predicted values. The observed data points (●) are shown as mean ± SD or mean only. The solid lines in D and E illustrates unity, as well as a twofold deviation from unity; whereas the data points represent observed mean ± SD values with respective to the simulated mean ± SD values for *AUC* (D) and *C*_*max*_ (E).

For Ritalin LA, a value of 0.5 (hr^b^) for *A* and a value of 2 (unitless) for *b* (Table 2) were utilized to predict plasma MPH concentration-time profiles in adults received 40 mg [22, 23] and 20 mg [9] Ritalin LA capsule under fasting conditions (Fig 4). Model-simulated and observed mean plasma concentration profiles of MPH contained within the IR beads, corresponding to the time course from 0 to 4 h, were in good agreement for all the data sets, except that the first time point for the study of [9] was largely overestimated (Fig 4A–4C). For the time course concentration profiles associated with the ER component, i.e. from 4 h to 24 h, observed peak plasma concentrations were somewhat under-predicted across all the data sets by 2 to 2.6-fold (Fig 4A–4C). In addition, model-predicted mean plasma MPH concentrations at 16 and 24 h for the study of [22] were approximately 2.2 and 3.3-fold of the observed values. Consistent with the discrepancy between model-predicted and observed mean plasma concentration time profiles, some degree of inaccuracy was noticed for the predicted pharmacokinetic model parameters. The current model properly predicted the average dose metrics of *C*_*max*_ over the time period from 0 to 4 h [9, 22, 23]; while model predictions of *C*_*max*_ from 4 h to 8 h [22] and 4 h to 10 h [23] were not considered adequate, with an error greater than two fold. In spite of inaccurate predictions of the second *C*_*max*_, model predictions of *AUC* met the evaluation criterion for all three data sets, with the predicted/observed mean values in the range of 0.55 to 0.84 (S3 Table, Fig 4D and 4E). For the *T*_*max*_, though the model-predicted average times to reach the first peak (*T*_*max1*_) [9, 22] and the second peak (*T*_*max2*_) [22] were consistent with observations, because of the failure to track the second peak concentrations, which were greater compared with the first one, the model predicted average *T*_*max*_ over a period of 24 h was imprecise compared with those found in the experimental data (2.6 versus 4.22 h and 2.6 versus 5.5 h) [9, 22] (S4 Table).

**Fig 5:**
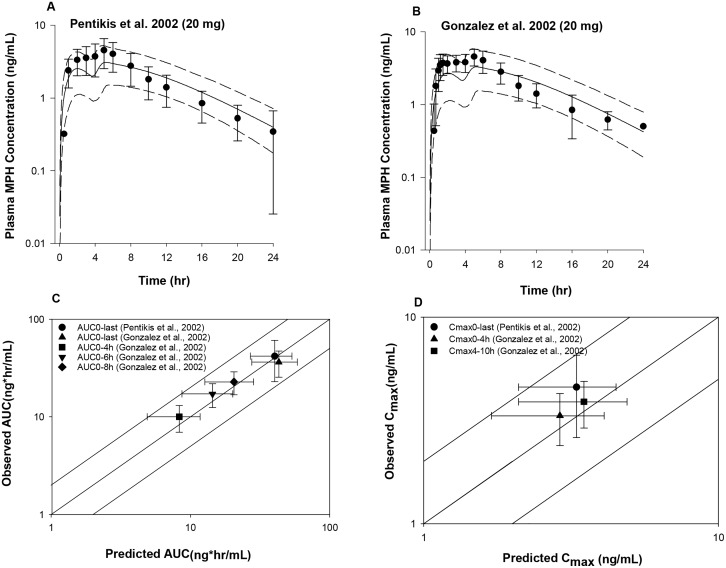
Model simulated versus observed total MPH plasma concentration profiles in adults receiving a single oral dose of Metadate CD at 20 mg [24, 25] under fasting conditions (A and B), as well as model predictability of the pharmacokinetic parameters *AUC* (C) and *C*_*max*_ (D). Solid lines in A-B represent simulated mean plasma concentration-time profiles, whereas dashed lines represent the 5^th^ and 95^th^ percentiles for the predicted values. The observed data points (●) are shown as mean ± SD. The solid lines in C and D illustrates unity, as well as a twofold deviation from unity; whereas the data points represent observed mean ± SD values with respective to the simulated mean ± SD values for *AUC* (C) and *C*_*max*_ (D).

For Metadate CD (Fig 5), a value of 0.5 (hr^b^) for *A* and a value of 2 (unitless) for *b* (Table 2) were visually fitted to achieve better agreement with observed plasma MPH concentration-time profiles in adults received a 20 mg Metadate CD capsule under fasting conditions [24, 25]. The model was capable of simulating plasma MPH concentration profiles resulting from exposure to oral Metadate CD for both data sets, with the difference of predicted and observed mean values within a factor of two, except that mean plasma MPH concentrations at 0.5 h in both studies were somehow overestimated by two to three-fold (Fig 5A and 5B). Consistent with the adequate description of the experimental plasma MPH concentration profiles, model-predicted pharmacokinetic parameters (*T*_*max*_, *C*_*max*,_ *AUC*) for both studies were in excellent agreement with observations indicated by the ratio of predicted/observed mean values within a twofold error range (S5 Table, Fig 5C and 5D). Of note, the lag time of 4 hours used in the current model is obtained by achieving agreement between model simulations and experimental data across all the ER formulations. This is somehow inconsistent with empirical evidences for Metadate CD, for which the ER component of Metadate CD was shown to release over 3–4 hours in vivo [1] and 1 hour after placement in an aqueous medium in vitro [57].

**Fig 6:**
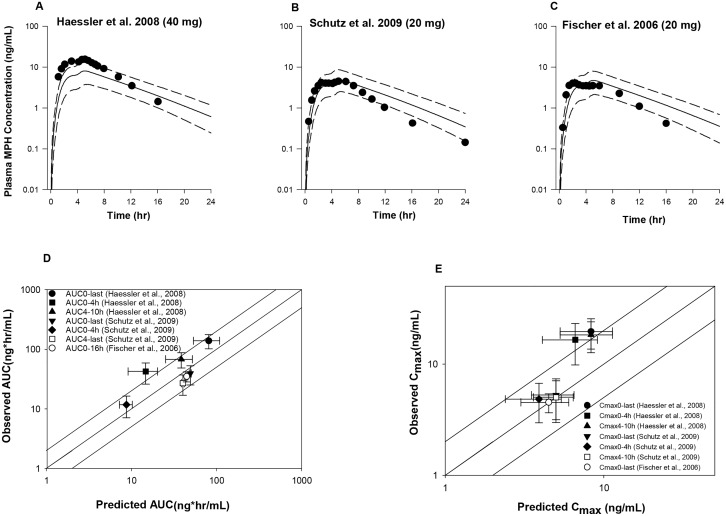
Model simulated versus observed total MPH plasma concentration profiles in adults receiving a single oral dose of Medikinet Retard at (A) 40 mg [23] and 20 mg [27, 28] (B and C) under fed conditions, as well as model predictability of the pharmacokinetic parameters *AUC* (D) and *C*_*max*_ (E). Solid lines in A-C represent simulated mean plasma concentration-time profiles, whereas dashed lines represent the 5^th^ and 95^th^ percentiles for the predicted values. The observed data points (●) are shown as mean except for Schutz et al (2009) where the observed data points are expressed as geometric mean. The solid lines in D and E illustrates unity, as well as a twofold deviation from unity; whereas the data points represent observed mean ± SD values (geometric mean ± SD values for the study of Schutz et al., 2009) with respective to the simulated mean ± SD values (geometric mean ± SD values for the study of Schutz et al., 2009) for *AUC* (D) and *C*_*max*_ (E).

For Medikinet Retard (Fig 6), a value of 0.5 (hr^b^) for *A* and a value of 2 (unitless) for *b* (Table 2) were visually fitted to achieve better agreement with observed plasma MPH concentration-time profiles in adults received with 20 mg [27, 28] and 40 mg [23] Medikinet Retard capsule under fed conditions. For the study of [23], observed mean plasma MPH concentrations were underestimated from time 0 to 4 hour by 2.3 to 4.5 fold [23], while for the remainder of the time course concentration profile, model simulations adequately reproduced collected kinetic data (Fig 6A). For the studies of [27] and [28], at earlier time points (0.5–1.5 h) observed mean plasma MPH concentrations were underestimated; while at later time points (12–24 h), mean plasma MPH concentrations were over-predicted compared with observations by approximately 2 to 3-fold (Fig 6B and 6C). Systemic clearance of MPH after approximately 8 hours appears faster than model-predicted across all the three data sets (Fig 6A–6C). Accordingly, some degrees of disparity between model-forcasted and experimental pharmacokinetic parameters (*T*_*max*_, *C*_*max*_, *AUC*) were observed (S6 Table). The observed average dose metrics of *C*_*max*_ and *AUC*_*0-4h*_ for the study of [58] were underestimated, with an error fold in the range of 0.3–0.4; while for the studies of [27] and [28], model predictions of pharmacokinetic parameters were considered adequate, with ratios of predicted/observed mean values for *AUC* and *C*_*max*_ within a factor of two (Fig 6D and 6E), except that the predicted *T*_*max*_ for the study of [28] was approxiamately 3 hours late compared with observations (2 h versus 5 h) (S6 Table).

### Sensitivity Analysis

**Fig 7:**
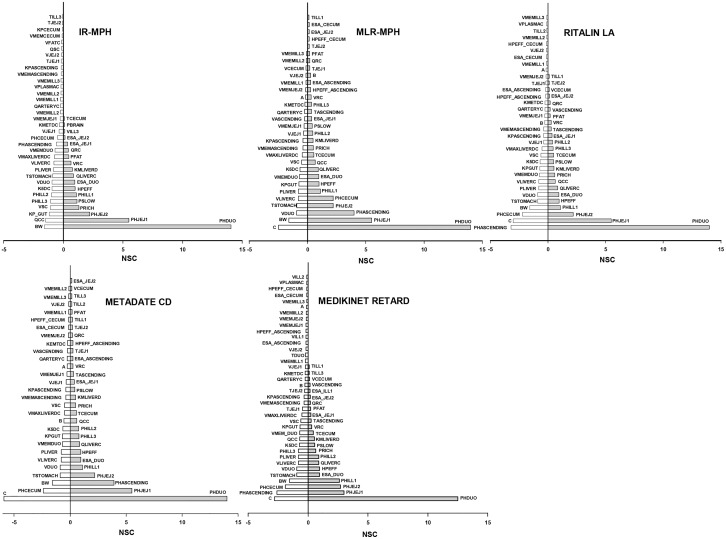
Sensitivity analysis. Model parameters with absolute normalized sensitivity coefficient (NSC) values greater than 0.1 are listed in the Figure.

Fig 7 shows sensitive model parameters with absolute NSC values greater than 0.1 based on the time course of MPH plasma concentration profiles over a period of 24 hour as model output. The definitions of model parameters are presented in Tables 1 and 2 and S1 Table.

For IR MPH, in addition to those model parameters identified to be sensitive in the original PBPK model [16], parameters representing the anatomy and physiology of the gastrointestinal tract also impact model predicted MPH plasma concentrations. Of these, the pH values of each small intestine section as well as the partition coefficient of the enterocytes, along with cardiac output, body weight, volume of slowly perfused tissues, partition coefficients of slowly and richly perfused tissues, appear to influence model output to a greater extent.

The sensitivity pattern was in general similar across different ER formulations, though the extent of the influence of model parameter perturbation on predicted MPH plasma concentrations as well as the correlations between model parameters and model output may vary. Compared with IR MPH, some model parameters associated with the cecum and ascending colon were found to be sensitive as well for the ER formulations but not for IR MPH. This suggests that the absorption of IR MPH predominantly occurs in the upper region of the small intestine, while for ER MPH, the cecum and ascending colon also contribute to a great extent to the absorption of MPH, primarily for the ER component. In addition, model parameters describing the Weibull function were found to influence model-predicted MPH plasma concentrations, of which the lag time significantly affect model output in an inverse manner.

Of note, the values shown in Fig 7 are the maximum absolute values over a period of 24 hours. The time course patterns of the influence of model parameters on model output vary across all the sensitive model parameters; some model parameters impact only a small portion of the time course, while others have a continuous effect throughout the period of 24 hours. In addition, the association of one specific model parameter with estimated MPH plasma concentrations may vary with time, i.e. positively correlated at some time points while negatively related at other time points (data not shown).

## Discussion

This work presents the extension of a recently published whole-body PBPK model for IR MPH [16] and delineates the pharmacokinetics of multiparticulate ER MPH formulations in humans with the integration of mechanistic description of the kinetic behaviors of MPH in the GI tract. Compared with a simple conventional approach, namely using the known i.v. profiles and bioavailability of the drug to implement a dual absorption model with one fraction absorbed fast and the second fraction absorbed more slowly, the utilization of highly sophisticated modeling of GI absorption to produce the oral dose profiles for products with rapidly absorbed and slowly absorbed components exhibits several advantages. First, the sophisticated description of the GI tract provides a mechanistic framework to account for the physiology of the different sections of the GI tract, physiochemical properties of MPH, and formulation related information. Second, in contrast to the empirical description of the oral absorption in the conventional approach, the absorption of MPH across the GI tract is accounted for by the physiology of the GI tract (e.g. pH, volume, surface area of each section, etc) and physiochemical properties of ER MPH (e.g. release kinetics, ionization, permeability, etc). This allows for the quantitative track of the kinetics of IR and ER components across the GI tract to account for the release, permeability and loss occurring in each section of the GI tract, which can be different across different sections. Therefore, this sophisticated approach could provide more insights into the diversity of the absorption profiles observed among different ER MPH formulations, from both physiological and formulation-related perspectives. Third, with the integration of inter-individual viabilities in those model parameters representing the physiology of the GI tract, the current model can be used to assess population variability of internal dose metrics of MPH following oral dosing of ER MPH. In addition, the mechanistic description of the GI tract allows for the evaluation of ER MPH kinetics under different physiological/pathological (e.g. some diseases affecting the physiology of the GI tract) and dosing conditions (e.g. fed conditions, co-administration of drugs mediating the pH of the GI tract).

Due to different model calibration strategies used in the current model and the previous one [59], i.e. the direct use of in vitro to in vivo extrapolation (IVIVE)-derived model parameters describing hepatic hydrolysis (*VmaxliverdC* and *VmaxliverlC*) in the current model versus the adjustment of these derived model parameters based on i.v. dosing data sets in the previous exercise [59], model parameters describing hepatic hydrolysis, and accordingly those representing hepatic oxidation as well as intestinal non-specific loss used in the current model differ substantially from those used in the previous model. However, the use of different physicochemical and pharmacokinetic parameters in these two exercises does not mean that the kinetics of MPH (e.g hepatic metabolism), except for oral absorption, should differ between IR and ER formulations. Such apparent dissimilarity is solely because of the different strategies used for model calibration. In addition, the renal clearance terms included in the previous model were used for the description of the systemic clearance of the metabolite ritalinic acid (RA) from the volume of distribution compartment, which was derived based on available urinary excretion and plasma concentration profiles of RA after IR MPH dosing. These clearance terms for RA after dosing of ER MPH in the current model are assumed to be the same as those determined in the previous model [59]. Since there is no available RA kinetic data after oral dosing of ER MPH to evaluate these renal clearance terms, these parameters were not reported in the current model.

In the current model, whole blood flow instead of plasma flow was utilized given that simulating whole blood flow to organs provides a physiologically realistic description of blood perfusion to tissue. Then calculating plasma concentration versus whole blood concentration can easily be carried out. To simulate plasma MPH concentrations, a plasma compartment was included as shown in Fig 1 and the volume of plasma was utilized. Due to the lack of raw data, observed mean plasma concentrations versus time profiles were compared with model predictions. To avoid the potential bias in comparing mean concentrations only, scatterplot visual prediction check (VPC) would be a highly useful tool to evaluate how well the model predictions overlay with original array of plasma concentrations if raw data sets are available.

Of note, in the current model, intestinal non-specific loss of MPH was introduced to achieve agreement with observed plasma MPH concentration profiles following oral dosing of IR MPH after the calibration of hepatic metabolic constants. The underlying mechanisms responsible for the non-specific loss in the GI tract remain unclear. One possibility is that MPH might undergo metabolism in the GI tract. While no direct information is available regarding the metabolism of MPH in the GI tract, the expression of hCES1A1 mRNA, the predominant human CES1 enzyme responsible for MPH hydrolysis as identified in human livers, has been observed in both small intestine and large intestine tissues (cecum and ascending colon) [60, 61]. Also, the observation of the hydrolysis of flurbiprofen derivatives [62], which are excellent substrates for hCES1 but not for hCES2, in human intestinal microsomes also suggests the existence of hCES1 enzyme in the GI tract. Moreover, as stated in the original paper [16], route-dependent discrepancies were found for plasma and urine profiles of *d*- and *l*-MPH as well as *d*- and *l*-RA in the first two hours following i.v. and oral administration [63, 64]. Higher plasma *d*-MPH concentrations compared with *l*-MPH concentrations appeared immediately following oral dosing, while after i.v. dosing such difference was not observed until 1.5 h. In addition, higher *l*-RA concentrations compared with *d*-RA concentrations in both human plasma and urine samples were noticed in the first 2 hours after oral dosing but not after i.v. dosing. These route-dependent discrepancies could imply potential enantioselective presystemic metabolism of MPH in the GI tract. However, such assumption could be misleading and bears uncertainties as well given that there exist controversial studies showing that there is no expression of CES1 in the GI tract [65]. Also CYP3A4 is the predominant gut oxidative CYP enzyme and does not appear to metabolize MPH [66]. Therefore, more studies are warranted to better understand the kinetic behaviors of MPH in the GI tract, e.g. metabolic stability studies with human intestinal preparations.

With some exceptions, plasma concentration profiles following a single oral dose of these ER MPH dosage forms in adult humans were reasonably well predicted using the established physiological absorption model for the first time. The current model describes the dosage forms containing multiple beads/pellets that are readily dispersed throughout the GI tract; while for Concerta^®^ using the OROS^®^ technology to deliver MPH by osmotic pressure, the tablet moves along the GI tract as a whole. Therefore, pharmacokinetic studies in adults with Concerta^®^ were not utilized for current model evaluation.

The present physiological absorption model predicted that the IR MPH is rapidly dissolved in the GI fluid, primarily in the stomach. The dissolved MPH moves along the GI tract and gets completely absorbed into enterocytes, with the majority of absorption occurring in the proximal portion of small intestine, predominantly in the jejunum perhaps due to the relative longer transit time, and only a very small portion absorbed in the cecum and the ascending colon (Table 3). Consistent with the IR formulation, the majority of the IR component in the ER formulations was also absorbed in the jejunum under fasting conditions, with fraction absorbed in each section varying along with the ratio of IR/ER component across different ER formulations; while under fed conditions, the absorption of the majority of the IR component occurred in the ileum, which was in part due to the decreased pH in the proximal portion (duodenum and jejunum) of small intestine under fed conditions, resulting in greater ionization for the basic MPH and herein decreased absorption across the intestinal epithelial cell membranes. In accordance with this, pH values of each section of the small intestine were determined to be sensitive in terms of impacting MPH plasma concentrations (Fig 7). In contrast to the IR component, because of the delay due to the engineered formulation technology, the model indicated that the release of ER component takes place primarily in cecum and ascending colon, with the majority of absorption occurring in the ascending colon (Table 3), which has been suggested previously as well [67]. In spite of the complete absorption of the IR component, the fraction of total MPH absorbed among different ER MPH formulations varied from 65–75%. The current model suggests that about 25–35% of the total dose moves to the distal portion of the colon and undergoes fecal excretion either as unreleased solid forms (19–27%) or dissolved forms (6–9%). Therefore, the low absolute oral bioavailability of ER MPH, which found to be comparable with that of IR MPH, i.e. approximately 30% for *d*-MPH and 1% for *l*-MPH [6, 20, 63, 68], could be in part due to the incomplete absorption of ER MPH [18, 69].

**Table 3:** Fraction of ER component released and total MPH absorbed in each section.

	IR:ER	Duodenum	Jejunum1	Jejunum2	Ileum1	Ileum2	Ileum3	Caecum	Ascending Colon	Total
**Released (% of ER component)**										
*MLR-MPH*	40:60	0	0.5	1.3	2	2.2	2	35	18.2	61
*Ritalin LA (SODAS)*	50:50	0	1	2.6	3.7	3.7	3.3	34.7	13.1	62
*Metadate CD (Diffucaps)*	30:70	0	1	2.6	3.7	3.7	3.3	34.7	13	62
*Medikinet Retard (enteric coated)*	50:50	0.3	3.6	5.3	5.8	5.1	4	28.8	9	62
**Absorbed (% of total dose)**										
*IR-MPH*	NA	6	26.3	23.9	18.8	13.9	8.4	0.5	1.6	99
*MLR-MPH*	40:60	2.2	9.8	9.2	7.8	6.5	4.9	4.6	21.4	66
*Ritalin LA (SODAS)*	50:50	3	13.3	12.5	10.6	8.9	6.6	3.8	15.6	74
*Metadate CD (Diffucaps)*	30:70	1.8	8.1	8	7.4	6.9	5.9	5.2	21.2	65
*Medikinet Retard (enteric coated)*	50:50	0.79	3	9.2	19.1	15.6	10.9	3.5	13.2	75

The disparate formulation technologies, along with the variable ratio of IR MPH to ER MPH components among different ER products, yield each a unique plasma concentration profile for each preparation. For MLR-MPH, the MPH is placed in different layers in each bead, where the IR component resides in the outermost bead layer and the ER component is situated in the innermost layer and surrounded by a delayed release and a controlled release coating [70]. There is a rapid initial release provided by the IR component, which accounts for 40% of the total dose, followed by a break and subsequent release of the ER part, with the second peak comparable to the first one, creating a bimodal plasma concentration profile [21]. For Ritalin LA with the SODAS technology, the delayed release beads that contain 50% of the total dose are polymer coated, which intervals the contact of the gastrointestinal fluid with the interior drug core of the beads [71, 72]. The latency of the release of the ER component depends on the penetration of the gastrointestinal fluid into the interior core of the beads and the erosion of the polymer coating [73]. There is also a double-peak plasma concentration profile similar to that of twice-daily IR MPH, for which the ER component (50% of total dose) provides a higher peak plasma concentration compared with the IR component [5]. For Metadate CD utilizing the Diffucaps technology, the neutral core is covered by active drug layer, followed by one or more functional membranes controlling the release rate of ER MPH [27]. Metadate CD produces a biphasic plasma concentration profile, during which a rapid initial absorption is followed by a gradual escalation of plasma MPH concentrations with a higher peak concentration accounting for 70% of the total dose observed. No evidence of a plateau in plasma concentration over the lunchtime period was observed [5]. Distinct from these formulations, Medikinet Retard, consisting of IR beads and enteric coated ER beads, needs to be administered with or after breakfast to achieve the biphasic profiles, where the second peak attributed to the 50% of the total dose is comparable or slightly lower compared with the first peak [6].

Unlike the IR MPH, for which the gastric emptying time primarily controls the absorption of MPH, the absorption of MPH from various ER dosage forms depends mostly on the programmed drug release and dissolution profiles [1]. The current model integrated a Weibull function to describe the release processes of MPH across different ER MPH formulations, allowing for the depiction of diverse absorption profiles resembling multiple doses of IR MPH to varying degrees. A lag time of 4 hour was introduced to account for the different mechanisms of delay before the drug is released, along with a visually fitted A and b parameters to designate the time course of release profiles. In general, the established physiological absorption model tracked the kinetic behaviors of plasma MPH concentrations following oral dosing of MLR-MPH and Metadate CD; while some improvements are needed to reproduce accurately the time courses of MPH plasma concentrations in adults receiving Ritalin LA and Medikinet Retard. The peak concentrations associated with the ER component were underestimated for adults received Ritalin LA, for which the rate and extent of MPH release from the inner core of the beads appear greater than model predictions using the Weibull function; while for Medikinet Retard, plasma clearance were underestimated to some extent following the second peak, perhaps implying the effect of food on the clearance of MPH. Therefore, the utilization of more complex mathematical models might be needed to account for the exact mechanisms underlying these different engineered release technologies and to track the diverse plasma kinetic profiles across different formulations [74].

In addition, instead of employing mathematical models to describe the in vivo release/dissolution profiles, an alternative approach to describe the in vivo release/dissolution rate is importing dissolution profiles obtained in vitro using bio-relevant media into the physiological absorption model. However, to the best of our knowledge, such information is not available in literature for different ER MPH formulations. One study examined the in vitro dissolution profiles for three different Ritalin LA formulations with different release rates, however, plasma concentration profiles as well as in vivo absorption were not significantly correlated with in vitro dissolution profiles observed in the same study [67]. Therefore, more studies to investigate the in vitro dissolution profiles of each ER MPH formulation using bio-relevant media may be helpful. Such information may potentially be integrated into the current model for better model performance.

There are several potential applications for the current adult physiological absorption model. One important application is to predict plasma MPH concentration profiles in children following oral administration of different ER MPH formulations. Considering the high prevalence rate of ADHD in school-aged children and the particular issues pertinent to MPH use at school, e.g. security and privacy, current clinical practices favor the prescription of the ER formulations. Thus, a better understanding of the pharmacokinetic profiles of different ER MPH products in children may assist clinicians with the development of optimized therapeutic regimens. Many PBPK models have been developed to investigate the exposure of children to chemicals or drugs, where adult models were scaled to children with the integration of age-specific physiological and biochemical differences [75], including the anatomy and physiology of the gastrointestinal tract. For MPH, although age-related physiological changes (e.g. blood flow and tissue volumes) have been extensively studied, chemical specific parameters, e.g. hydrolysis and oxidation of MPH in the liver and potentially in the small intestine, as a function of age, have not been characterized fully in a quantitative manner. In particular, because of the lack of knowledge on the specific enzymes involved in MPH oxidation, inferring age-specific oxidation constants for MPH from adults to young children involves more uncertainties. In addition, although efforts have been made to estimate the age-dependent anatomy and physiology of the GI tract, many assumptions need to be tested and verified. Therefore, in order to reduce the uncertainty for the estimation of internal dose metrics of MPH in young children, further studies are needed when extrapolating adult human models to children. Of particular importance are metabolic studies with MPH using various in vitro systems, e.g. microsomes and hepatocytes, from different age groups to derive age-dependent hepatic and gastrointestinal hydrolysis and oxidation metabolic constants, along with the quantitative characterization of the anatomy and physiology of the GI tract across different developmental stages.

Another potential extension of the current model is to evaluate the food effect on the pharmacokinetics of different ER MPH formulations. It has been observed that following a high-fat meal, there is a possibility that the time to reach the peak concentrations (*T*_*max*_) may be delayed probably due to the delayed gastric emptying (e.g. Metadate CD), along with potential inconsistent changes in peak concentrations (*C*_*max*_), suggesting a potential temporal association with high fat intake [1]; while for some dosage forms (e.g Ritalin LA), such correlation was not observed [22]. In addition, soft food, such as applesauce, was found to have no impact on the bioavailability and kinetic profiles of some dosage forms (e.g. Metadate CD, Ritalin LA, MLR-MPH) given as applesauce/sprinkle mix [6, 22, 24], implying that the effect of food on the extent and rate of absorption for ER MPH formulations may vary depending on the of composition the food as well as the formulation itself. Further, some studies have indicated that in addition to the impact on *T*_*max*_ and *C*_*max*_, administration of ER MPH dosage forms under different fed conditions (e.g. high fat versus standard breakfast) could result in altered biphasic plasma MPH concentration profiles (e.g. MLR-MPH) [76], indicating that the food itself, along with their effects on the physiology of the GI tract, may influence the programmed release and dissolution patterns of some ER formulations. Therefore, additional studies are needed to understand fully the kinetics of different ER formulations under fed conditions, allowing for the integration of such knowledge into the current model for the assessment of plasma MPH concentration time courses under various fed conditions with more confidence.

Last, but not the least, it has been suggested that the magnitude of symptom reduction by MPH follows its plasma concentration profiles [11, 77], in addition to the association between clinical response with the rate of rise in blood drug concentrations (the “ramp” effect) being proposed [1]. That is, a potential positive association is observed between blood MPH concentrations and clinical responses [2, 12], unless adverse effects exceed the efficacies [21]. The formulation with the highest plasma MPH concentrations at any specific time points usually yields better control of ADHD symptoms compared with other formulations [2]. Therefore, the prediction of the time courses of plasma MPH concentrations using the current model with the integration of patient-specific physiological conditions can help estimate the extent of the clinical effects throughout the day [2]. Such knowledge may assist the clinicians in the process of selecting appropriate MPH formulations to best fit the patient’s individual needs, ensuring symptom controls during the particular circumstances throughout the day. Moreover, understanding the potential differences in the plasma concentration time courses of MPH among various ER MPH formulations can help with the dose adjustment when converting a patient from one MPH ER formulation to another [21]. For example, Ritalin LA had a higher initial *C*_*max*_ compared with MLR-MPH, so it may be appropriate to start with a relatively higher dose when converting a patient from Ritalin LA to MLR MPH. In addition, the establishment of the association between dose and clinic effects with the incorporation of the pharmacodynamic model [78] into the current model would provide valuable insights into the time course profiles of the clinical outcome and possible side effects, offering guidance on individualized optimum treatment for ADHD for maximum efficacy [11].

In addition, the modeling approaches, e.g. the mathematical description of the dissolution, permeability, metabolism, distribution, etc, developed in the current study can be used for other drug products, including both immediate release and extended release formulations. With the integration of drug-specific information (physicochemical properties, such as solubility, Log P, pKa, and partition coefficient, etc; and metabolism kinetics) and formulation-related information (release characteristics described using Weibull function or in vitro dissolution profiles in bio-relevant media) into the current model, the absorption profile and disposition of the drug can be described in a quantitative manner. As discussed previously, compared with non-compartmental or compartmental classic pharmacokinetic modeling, the mechanistic platform established in the current model can better assist the understanding of the kinetic behaviors of other drugs in the GI tract, as well as their distribution and disposition in the system.

## Conclusion

In the current study, a physiological absorption model was developed to characterize the pharmacokinetics of MPH in adults following oral dosing of both IR and ER MPH dosage forms. With the integration of information on the anatomy and physiology of the GI tract as well as MPH physicochemical properties and formulation related information, the current model provides a mechanistic description of the kinetic behaviors of ER MPH in the GI tract, instead of an empirical first-order absorption rate constant as in the original model. The model performs well compared with observed data, with excellent fit to plasma concentration profiles of MPH in adult humans receiving IR MPH, suggesting the success of the model for the description of MPH absorption and disposition in the body. For ER MPH, the model, in general, tracked the kinetic behavior of plasma MPH in adults dosed with MLR-MPH and Metadate CD; while for Ritalin LA and Medikinet Retard, some discrepancy between model predictions and observations were observed, implying the necessity of employing more complicated mathematical approaches to describe the release of MPH from these formulations. The establishment of the current model provides a valuable basis for the evaluation of ER MPH kinetics in children, as well as the assessment of food effect; it also offers assistance for the clinician in choosing the optimized dosage regimens to best fit the patient’s individual needs. In addition, the practice of the current model development provides some insights into the potential factors responsible for the observed diversity of MPH plasma concentration profiles among different ER formulations: 1) the ratio of IR/ER components across different ER formulations; 2) fasting/fed condition, for which gastric emptying and intestinal absorption rates vary; 3) engineered release profiles.

## Supporting Information

S1 File PBPK modeling code. (PDF)

S1 Table Distribution of parameters used for population analysis. (DOC)

S2 Table Model predicted versus observed pharmacokinetic model parameters for subjects receiving IR MPH under fasting conditions. (DOC)

S3 Table Model predicted versus observed pharmacokinetic model parameters for subjects receiving MLR MPH under fasting conditions. (DOC)

S4 Table Model predicted versus observed pharmacokinetic model parameters for subjects receiving Ritalin-LA under fasting conditions. (DOC)

S5 Table Model predicted versus observed pharmacokinetic model parameters for subjects receiving Metadate CD under fasting conditions. (DOC)

S6 Table Model predicted versus observed pharmacokinetic model parameters for subjects receiving Medikinet Retard under fed conditions. (DOC)
