# Supplementary material for: Application of Physiologically Based Absorption Modeling to Characterize the Pharmacokinetic Profiles of Oral Extended Release Methylphenidate Products in Adults
Source: PLoS One. 2016 Oct 10;11(10):e0164641. doi: 10.1371/journal.pone.0164641 (PMC5056674; doi:10.1371/journal.pone.0164641)
Supplement: S1 File — (PDF) [file pone.0164641.s001.pdf]

```
    PROGRAM: PBPK model for extended release Methylphenidate (MPH)in adult
humans.
    !!!Note parameter values in the csl codes may be replaced by m files

    INITIAL
    !!!%%%%%%%%%%%%%%%%%%%%%%%%%%%%%%% MODEL CONSTANTS USED FOR the absorption
model %%%%%%%%%%%%%%%%%%%%%%%%%%%%
    !!EXCHANGE SURFACE AREA, CM^2
    CONSTANT ESA_DUO           =  19995
    CONSTANT ESA_JEJ1          =  77482
    CONSTANT ESA_JEJ2          =  69217
    CONSTANT ESA_ILL1          =  60952
    CONSTANT ESA_ILL2          =  52171
    CONSTANT ESA_ILL3          =  43906
    CONSTANT ESA_CECUM         =  1964
    CONSTANT ESA_ASCENDING     =  2961

    !!INTESTINAL TRANSIT TIMES ARE IN HOUR (Almukainzi 2014)
    CONSTANT TSTOMACH          =  0.25
    CONSTANT TDUO              =  0.26
    CONSTANT TJEJ1             =  0.95
    CONSTANT TJEJ2             =  0.76
    CONSTANT TILL1             =  0.59
    CONSTANT TILL2             =  0.43
    CONSTANT TILL3             =  0.31
    CONSTANT Tcecum            =  4.5
    CONSTANT Tascending        =  13.5

    !!INTESTINAL PH values (Almukainzi 2014)
    CONSTANT PHSTOMACH         =  1.3
    CONSTANT PHDUO             =  6.0
    CONSTANT PHJEJ1            =  6.2
    CONSTANT PHJEJ2            =  6.4
    CONSTANT PHILL1            =  6.6
    CONSTANT PHILL2            =  6.9
    CONSTANT PHILL3            =  7.4
    CONSTANT PHcecum           =  6.4
    CONSTANT PHascending       =  6.8

    !!Volume of the lumen (mL)(Almukainzi 2014)
    CONSTANT VSTOMACH          =  50
    CONSTANT VDUO              =  48
    CONSTANT VJEJ1             =  175
    CONSTANT VJEJ2             =  140
    CONSTANT VILL1             =  109
    CONSTANT VILL2             =  79
    CONSTANT VILL3             =  56
    CONSTANT Vcecum            =  53
    CONSTANT Vascending        =  57

    !!PHYSCHEM PROPERTIES
    CONSTANT ION               =  1         !NOTE -1=Acid, 0=Neutral, 1=Base ,
2= Zwitterion  !!Singh et al. 2005
    CONSTANT PKABASE1          =  8.77      !Singh et al. 2005
    CONSTANT PKABASE2          =  8.77
    CONSTANT PKAACID1          =  16
    CONSTANT PKAACID2          =  16
    CONSTANT MW                =  233.3062  !g/mol
    CONSTANT MW_WATER          =  18.02     !g/mol

    !!SOLUBILITY- Estimated using ADMET Predictor (Simulations Plus, Inc.)
    CONSTANT SOL               =  9250      !mg/L
    CONSTANT REFPHSOL          =  10.58     !reference pH of the user
solubility

    !!LOGICAL CONSTANTS
```

```
    LOGICAL  INTESTINAL
    CONSTANT INTESTINAL        =  0         !Not intestinal SOL, False

    !!CONSTANTS FOR Permeabilty ESTIMATION (HPEFF AND DIFF)
    CONSTANT CACO2PEFF            = 24.7! MDCKII 24.7 x 10^-6 cm/s; in the 
form of 1 not 1e10^-6 cm/s;(Feng 2008)
    CONSTANT CACO2PEFF_cecum      = 24.7!
    CONSTANT CACO2PEFF_ascending  = 24.7!

    !For dissolution constant (Kd)calculation (dissolve)
    CONSTANT DIFFCOEFF_IR         = 1e-4                        !cm^2/min, 
default value 10-4 cm2/min
    CONSTANT rho                  = 1                           !particule 
density in g/mL
    CONSTANT rparticle            = 5.0                         !radius in um, 
default value 5 um; effective drug particle radius !1 um = 0.0001 cm
    CONSTANT DLT                  = 30                          !diffusion 
layer thickness in um, defult 30 um

                         !!!!!!!!!!!!!!!!!!! Physiological Parameters 
!!!!!!!!!!!!!!!!!!!

    ! Fractional Blood Flows to Tissues (fraction of cardiac output)

    CONSTANT QCC                  = 15.87       ! (L/h/kg^0.75) |Cardiac 
output ACAT
    CONSTANT QFatC                = 0.053       ! |Fractional blood flow to 
the fats ; female: 0.091
    CONSTANT QbrainC              = 0.11        ! |Fractional blood flow to 
the brain ;
    Constant QgonadC              = 0.00054     ! |Fractional blood flow to 
the gonads; female: 0.00022
    CONSTANT QHeartC              = 0.038       ! |Fractional blood flow to 
the heart; female: 0.047
    CONSTANT QLiverC              = 0.255       ! |Fractional blood flow to 
the liver: total blood flow, portal plus arterial;0.27 for female
    CONSTANT QDUOC                = 0.02375     ! !Fractioanl blood flow to 
the duo; 0.025625 for females obtained by QgutC/8
    CONSTANT QJEJ1C               = 0.02375     !0.025625
    CONSTANT QJEJ2C               = 0.02375     !0.025625
    CONSTANT QILL1C               = 0.02375     !0.025625
    CONSTANT QILL2C               = 0.02375     !0.025625
    CONSTANT QILL3C               = 0.02375     !0.025625
    CONSTANT QCECUMC              = 0.02375     !0.025625
    CONSTANT QASCENDINGC          = 0.02375     !0.025625

    ! Fractional Tissue Volumes of BW

    CONSTANT BW                   = 80          !(kg)         |Body weight
    CONSTANT VplasmaC             = 0.0435      !(%BW)        |Fractional 
Volume of the Plasma
    CONSTANT VFatC                = 0.213       !(%BW)        |Fractional 
Volume of the fat; female: 0.327
    CONSTANT VbrainC              = 0.02        !(%BW)        |Fractional 
Volume of the brain
    CONSTANT VgonadC              = 0.0007      !(%BW)        |Fractional 
Volume of the gonads; female: 0.0027
    CONSTANT VHeartC              = 0.0045      !(%BW)        |Fractional 
Volume of the heart; female: 0.0042
    CONSTANT VLiverC              = 0.026       !(%BW)        |Fractional 
Volume of the liver
    CONSTANT VbodyC               = 0.6         !(%BW)        |Fractional 
Volume of the body
    CONSTANT VMEMDUOC             = 0.000767    !Female: 0.000877 Valentin 
2002 Table 6.6 and 6.7
    CONSTANT VMEMJEJ1C            = 0.00192     !Female 0.00192
```

```
    CONSTANT VMEMJEJ2C            = 0.00192     !Female 0.00192
    CONSTANT VMEMILL1C            = 0.00142     !Female 0.00149
    CONSTANT VMEMILL2C            = 0.00142     !Female 0.00149
    CONSTANT VMEMILL3C            = 0.00142     !Female 0.00149
    CONSTANT VMEMCOLONMEMC        = 0.00125     !Female 0.00137 |Fractional
Weight of cecum and asending colon  obtained from ICRP Publication 89 , Table
6.9, 6.10
    CONSTANT VMEMCECUMMEMC        = 0.0004      !!0.0004    !Adjusted based on
length
    CONSTANT VMEMASCENDINGMEMC    = 0.000848    !!0.00093

                        !!!!!!!!!!!!!!!!!!! Chemical Specific
Parameters!!!!!!!!!!!!!!!!!!

    ! Partition Coefficients for MPH

    CONSTANT PFat                 = 1.79 !(no units)|Partitioning into the fat
  (Fat/blood)
    CONSTANT Pbrain               = 6.07 !(no units)|Partitioning into the
brain (Brain/blood)
    CONSTANT PRich                = 5.66 !(no units)|Partitioning into the
richly perfused tissues (Richly perfused/blood)
    CONSTANT PSlow                = 2.47 !(no units)|Partitioning into the
slowly perfused tissues (Slowly perfused/blood)

    CONSTANT PGonad               = 3.12 !(no units)|Partitioning into the
gonads (Gonads/blood)
    CONSTANT Pheart               = 2.19 !(no units)|Partitioning into the
heart (Heart/blood)
    CONSTANT PLiver               = 5.66 !(no units)|Partitioning into the
liver (Liver/blood)
    CONSTANT KPGUT                = 5.66 !set to the value of liver;
    CONSTANT KPcolon              = 5.66!

    ! Kinetic Parameters

    !! MPH oral uptake and metabolism

    CONSTANT Kmliverd             = 27600!(ug/L)        |Km of d-MPH
hydrolysis in the liver
    CONSTANT Kmliverl             = 10172!(ug/L)        |Km of l-MPH
hydrolysis in the liver
    CONSTANT VmaxliverdC          = 25826!(ug/h/kg^0.75)|Vmax of d-MPH
hydrolysis in the liver
    CONSTANT VmaxliverlC          = 52404!(ug/h/kg^0.75)|Vmax of l-MPH
hydrolysis in the liver
    CONSTANT kmetdC               = 0.43 !(L/h/kg^0.75) |Clearance term of
d-MPH oxidation in the liver
    CONSTANT KmetlC               = 0.43 !(L/h/kg^0.75) |Clearance term of
l-MPH oxidation in the liver
    CONSTANT K5lC                 = 37   !(1/h/kg^0.75) |Metabolism of d-MPH
in the small intestine
    CONSTANT K5dC                 = 0.78 !(1/h/kg^0.75) |Metabolism of l-MPH
in the small intestine
    CONSTANT K5lC_cecum           = 37   !(1/h/kg^0.75) |Metabolism of d-MPH
in the cecum
    CONSTANT K5dC_cecum           = 0.78 !(1/h/kg^0.75) |Metabolism of l-MPH
in the cecum
    CONSTANT K5lC_ascending       = 37   !(1/h/kg^0.75) |Metabolism of d-MPH
in the ascending colon
    CONSTANT K5dC_ascending       = 0.78 !(1/h/kg^0.75) |Metabolism of l-MPH
in the ascending colon
    CONSTANT F                    = 0.8  !(no units)    |Fraction of MPH
metabolized in the gut undergoing hydrolysis

    !! Urinary excretion of RA
```

```
    CONSTANT Ku_RAdC              = 0.305       !(L/h/kg^0.75) |Urinary
excretion of d-RA
    CONSTANT Ku_RAlC              = 0.168       !(L/h/kg^0.75) |Urinary
excretion of l-RA

    !!!!!!!!!!!!!!! Dosing Parameters!!!!!!!!!!!!!!!!!

    CONSTANT TSTOP                = 24         !(h)            |simulatioin
period
    CONSTANT tlen                 = 0.05       !(h)            |Length of oral
gavage exposure
    CONSTANT IVTime               = 0.01       !(h)            |Length of IV
dosing
    CONSTANT IVdoseCd             = 0.0        !(ug/Kg)        |IV dose for
d-MPH
    CONSTANT IVdoseCl             = 0.0        !(ug/Kg)        |IV dose for
l-MPH
    CONSTANT AdosedIR             = 0.0        !(ug)           |Oral dose of
IR-dMPH
    CONSTANT AdosedER             = 0.0        !(ug)           |Oral dose of
ER-dMPH
    CONSTANT AdoselIR             = 0.0        !(ug)           |Oral dose of
IR-lMPH
    CONSTANT AdoselER             = 0.0        !(ug)           |Oral dose of
ER-lMPH

    !!MW, MPHHCL = 269.77; MPH: 233.3062; RA: 219.28

     END ! INITIAL


    DYNAMIC

                                ALGORITHM IALG        = 1!
                                NSTEPS    NSTP        = 1
                                MAXTERVAL MAXT        = 1.0e9
                                MINTERVAL MINT        = 1.0e-9
                                CINTERVAL CINT        = 0.01!

DERIVATIVE

    !scaled blood flows

    QC                         = QCC*BW^0.75
!(L/h)|Cardiac Output
    QFat                       = QFatC*QC                              !(L/h)|Blood
flow to the fat
    QBrain                     = QBrainC*QC                            !(L/h)|Blood
flow to the brain
    Qgonad                     = QgonadC*QC                            !(L/h)|Blood
flow to the gonads
    QLiver                     = QLiverC*QC                            !(L/h)|Blood
flow to the liver
    QHeart                     = QHeartC*QC                            !(L/h)|Blood
flow to the heart
    QS                         = 0.24*QC - QFat-Qheart                 !(L/h)|Blood
flow to the slowly perfused tissues
    QR                         = 0.76*QC - QLiver - Qgonad - Qbrain  !(L/h)|Blood
flow to the rapidly perfused tissues
    QGUT                       = QGUTC*QC                              !(L/h)|Blood
flow to small intestine
    QDUO                       = QDUOC*QC                              !(L/h)|Blood
flow to Duodenum
    QJEJ1                      = QJEJ1C*QC
    QJEJ2                      = QJEJ2C*QC
```

```
    QILL1                   = QILL1C*QC
    QILL2                   = QILL2C*QC
    QILL3                   = QILL3C*QC
    QCECUM                  = QCECUMC*QC
    QASCENDING              = QASCENDINGC*QC

    !Scaled tissue volumes

    VLiver                  = VLiverC*BW
!(L)|Volume of the liver
    VFat                    = VFatC*BW
!(L)|Volume of the fat
    Vgonad                  = VgonadC*BW
!(L)|Volume of the gonads
    VS                      = 0.60*BW -VFat-Vheart
!(L)|Volume of the slowly perfused tissues
    VR                      = 0.33*BW -VLiver-Vplasma
-Vgonad-Vbrain-VMEMDUO-VMEMJEJ1-VMEMJEJ2-VMEMILL1-VMEMILL2-VMEMILL3
-VMEMCECUM-VMEMASCENDING !(L)|volume of the rapidly perfused tissues
    VPlasma                 = VPlasmaC*BW
!(L)|Volume of the plasma
    Vbrain                  = VbrainC*BW
!(L)|Volume of the brain
    Vheart                  = VheartC*BW
!(L)|Volume of the heart
    VMEMDUO                 = VMEMDUOC*BW
!(L)|Volume of the Duodenum
    Vbody                   = VbodyC*BW
!(L)|Volume of the body compartment
    VMEMCECUM               = BW*VMEMCECUMMEMC
    VMEMASCENDING           = BW*VMEMASCENDINGMEMC
    VMEMJEJ1                = VMEMJEJ1C*BW
    VMEMJEJ2                = VMEMJEJ2C*BW
    VMEMILL1                = VMEMILL1C*BW
    VMEMILL2                = VMEMILL2C*BW
    VMEMILL3                = VMEMILL3C*BW

   !!!$$$$$$$$$$$$$$$$$$$$$$$$$$$$$$$$$$$$$$$$$ SET UP GUT PARAMETERS
$$$$$$$$$$$$$$$$$$$$$$$$$$$$$$$$!!!!! ref: Alex Avdeef 2007 solubility of
sparingly-soluble ionizable drugs

    !Ionisation terms
    IF (ION .EQ. 1 .AND. PKABASE1 .EQ. PKABASE2) THEN ! monoprotic base
    HHINT       = 1+(10.0**(PKABASE1 - REFPHSOL))
    HHSTOMACH   = 1+(10.0**(PKABASE1 - PHSTOMACH))
    HHDUO       = 1+(10.0**(PKABASE1 - PHDUO))
    HHJEJ1      = 1+(10.0**(PKABASE1 - PHJEJ1))
    HHJEJ2      = 1+(10.0**(PKABASE1 - PHJEJ2))
    HHILL1      = 1+(10.0**(PKABASE1 - PHILL1))
    HHILL2      = 1+(10.0**(PKABASE1 - PHILL2))
    HHILL3      = 1+(10.0**(PKABASE1 - PHILL3))
    HHCECUM     = 1+(10.0**(PKABASE1 - PHCECUM))
    HHASCENDING = 1+(10.0**(PKABASE1 - PHASCENDING))

  ELSE IF (ION .EQ. 1) THEN ! diprotic base
    HHINT       = 1+(10.0**(max(PKABASE1, PKABASE2) - REFPHSOL) +
10.0**(PKABASE1 + PKABASE2 - 2.0*REFPHSOL))
    HHSTOMACH   = 1+(10.0**(max(PKABASE1, PKABASE2) - PHSTOMACH) +
10.0**(PKABASE1 + PKABASE2 - 2.0*PHSTOMACH))
    HHDUO       = 1+(10.0**(max(PKABASE1, PKABASE2) - PHDUO) + 10.0**(PKABASE1
+ PKABASE2 - 2.0*PHDUO))
    HHJEJ1      = 1+(10.0**(max(PKABASE1, PKABASE2) - PHJEJ1) +
10.0**(PKABASE1 + PKABASE2 - 2.0*PHJEJ1))
    HHJEJ2      = 1+(10.0**(max(PKABASE1, PKABASE2) - PHJEJ2) +
10.0**(PKABASE1 + PKABASE2 - 2.0*PHJEJ2))
    HHILL1      = 1+(10.0**(max(PKABASE1, PKABASE2) - PHILL1) +
```

```
10.0**(PKABASE1 + PKABASE2 - 2.0*PHILL1))
    HHILL2      = 1+(10.0**(max(PKABASE1, PKABASE2) - PHILL2) +
10.0**(PKABASE1 + PKABASE2 - 2.0*PHILL2))
    HHILL3      = 1+(10.0**(max(PKABASE1, PKABASE2) - PHILL3) +
10.0**(PKABASE1 + PKABASE2 - 2.0*PHILL3))
    HHASCENDING = 1+(10.0**(max(PKABASE1, PKABASE2) - PHASCENDING) +
10.0**(PKABASE1 + PKABASE2 - 2.0*PHASCENDING))
    HHcecum     = 1+(10.0**(max(PKABASE1, PKABASE2) - PHcecum) +
10.0**(PKABASE1 + PKABASE2 - 2.0*PHcecum))

  ELSE IF (ION .EQ. -1 .AND. PKAACID1 .EQ. PKAACID2) THEN ! monoprotic acid
    HHINT       = 1+(10.0**(REFPHSOL - PKAACID1))
    HHSTOMACH   = 1+(10.0**(PHSTOMACH - PKAACID1))
    HHDUO       = 1+(10.0**(PHDUO - PKAACID1))
    HHJEJ1      = 1+(10.0**(PHJEJ1 - PKAACID1))
    HHJEJ2      = 1+(10.0**(PHJEJ2 - PKAACID1))
    HHILL1      = 1+(10.0**(PHILL1 - PKAACID1))
    HHILL2      = 1+(10.0**(PHILL2 - PKAACID1))
    HHILL3      = 1+(10.0**(PHILL3 - PKAACID1))
    HHCECUM     = 1+(10.0**(PHCECUM- PKAACID1))
    HHASCENDING = 1+(10.0**(PHASCENDING - PKAACID1))

  ELSE IF (ION .EQ. -1) THEN ! diprotic acid
    HHINT       = 1+(10.0**( REFPHSOL - min(PKAACID1, PKAACID2)) +
10.0**(2*REFPHSOL - PKAACID1 - PKAACID2))
    HHSTOMACH   = 1+(10.0**(PHSTOMACH - min(PKAACID1, PKAACID2)) +
10.0**(2*PHSTOMACH - PKAACID1 - PKAACID2))
    HHDUO       = 1+(10.0**(PHDUO - min(PKAACID1, PKAACID2)) + 10.0**(2*PHDUO
- PKAACID1 - PKAACID2))
    HHJEJ1      = 1+(10.0**(PHJEJ1 - min(PKAACID1, PKAACID2)) +
10.0**(2*PHJEJ1 - PKAACID1 - PKAACID2))
    HHJEJ2      = 1+(10.0**(PHJEJ2 - min(PKAACID1, PKAACID2)) +
10.0**(2*PHJEJ2 - PKAACID1 - PKAACID2))
    HHILL1      = 1+(10.0**(PHILL1 - min(PKAACID1, PKAACID2)) +
10.0**(2*PHILL1 - PKAACID1 - PKAACID2))
    HHILL2      = 1+( 10.0**(PHILL2 - min(PKAACID1, PKAACID2)) +
10.0**(2*PHILL2 - PKAACID1 - PKAACID2))
    HHILL3      = 1+(10.0**(PHILL3 - min(PKAACID1, PKAACID2)) +
10.0**(2*PHILL3 - PKAACID1 - PKAACID2))
    HHCECUM     = 1+(10.0**(PHCECUM- min(PKAACID1, PKAACID2)) +
10.0**(2*PHCECUM- PKAACID1 - PKAACID2))
    HHASCENDING = 1+(10.0**(PHASCENDING- min(PKAACID1, PKAACID2)) +
10.0**(2*PHASCENDING- PKAACID1 - PKAACID2))

  ELSE IF (ION .EQ. 2) THEN !zwitterion
    HHINT       = 1+(10.0**(max(PKABASE1, PKABASE2) - REFPHSOL) +
10.0**(REFPHSOL - min(PKAACID1, PKAACID2)))
    HHSTOMACH   = 1+(10.0**(max(PKABASE1, PKABASE2) - PHSTOMACH) +
10.0**(PHSTOMACH - min(PKAACID1, PKAACID2)))
    HHDUO       = 1+(10.0**(max(PKABASE1, PKABASE2) - PHDUO) + 10.0**(PHDUO -
min(PKAACID1, PKAACID2)))
    HHJEJ1      = 1+(10.0**(max(PKABASE1, PKABASE2) - PHJEJ1) + 10.0**(PHJEJ1
- min(PKAACID1, PKAACID2)))
    HHJEJ2      = 1+(10.0**(max(PKABASE1, PKABASE2) - PHJEJ2) + 10.0**(PHJEJ2
- min(PKAACID1, PKAACID2)))
    HHILL1      = 1+(10.0**(max(PKABASE1, PKABASE2) - PHILL1) + 10.0**(PHILL1
- min(PKAACID1, PKAACID2)))
    HHILL2      = 1+(10.0**(max(PKABASE1, PKABASE2) - PHILL2) + 10.0**(PHILL2
- min(PKAACID1, PKAACID2)))
    HHILL3      = 1+(10.0**(max(PKABASE1, PKABASE2) - PHILL3) + 10.0**(PHILL3
- min(PKAACID1, PKAACID2)))
    HHCECUM     = 1+( 10.0**(max(PKABASE1, PKABASE2) - PHCECUM) +
10.0**(PHCECUM - min(PKAACID1, PKAACID2)))
    HHASCENDING = 1+( 10.0**(max(PKABASE1, PKABASE2) - PHASCENDING) +
10.0**(PHASCENDING - min(PKAACID1, PKAACID2)))
```

```
  ELSE ! Neutrals
    HHINT       = 1.0
    HHSTOMACH   = 1.0
    HHDUO       = 1.0
    HHJEJ1      = 1.0
    HHJEJ2      = 1.0
    HHILL1      = 1.0
    HHILL2      = 1.0
    HHILL3      = 1.0
    HHCECUM     = 1.0
    HHASCENDING = 1.0
  ENDIF

  !Correct solubility for reference PH
  !Solubility intrinsic, int, in mg/L ug/mL
  !SOL is the input solubility in water, fessif or fassif at the reference PH
in mg/L
  SOLINT       = SOL/HHINT

  !SOLWATER corrected for each compartment in mg/l to account for effect of PH
(ionization)
  SOLWATER_STOMACH  = SOLINT*HHSTOMACH
  SOLWATER_DUO      = SOLINT*HHDUO
  SOLWATER_JEJ1     = SOLINT*HHJEJ1
  SOLWATER_JEJ2     = SOLINT*HHJEJ2
  SOLWATER_ILL1     = SOLINT*HHILL1
  SOLWATER_ILL2     = SOLINT*HHILL2
  SOLWATER_ILL3     = SOLINT*HHILL3
  SOLWATER_CECUM    = SOLINT*HHCECUM
  SOLWATER_ASCENDING= SOLINT*HHASCENDING

  !Correct solubility for bile effect
  !Mithani 1996 Estimation of the increase in solubility of drugs as a
function of bile salt concentration

  CONSTANT LOGP = 0.2

  IF (INTESTINAL) THEN
  SOLBILE = 0
  ELSE
  LOGSR            =0.606*LOGP+2.234 !bile salt solubilization ratio (SR)
  SOLBILE_STOMACH  =(exp(LOGSR))*SOLWATER_STOMACH*(MW_WATER/MW)*1e-6
!Solubility in Bile (SOLBILE)= SR*SCaq; !SCaq (aqueous solubilization
capacity)=(SOL*MW_WATER/MW) = convert sol (mg/L) to moles drug/mol water,
unitless, aquesous solubilization capacity
  SOLBILE_DUO      =(exp(LOGSR))*SOLWATER_DUO*(MW_WATER/MW)*1e-6
  SOLBILE_JEJ1     =(exp(LOGSR))*SOLWATER_JEJ1*(MW_WATER/MW)*1e-6
  SOLBILE_JEJ2     =(exp(LOGSR))*SOLWATER_JEJ2*(MW_WATER/MW)*1e-6
  SOLBILE_ILL1     =(exp(LOGSR))*SOLWATER_ILL1*(MW_WATER/MW)*1e-6
  SOLBILE_ILL2     =(exp(LOGSR))*SOLWATER_ILL2*(MW_WATER/MW)*1e-6
  SOLBILE_ILL3     =(exp(LOGSR))*SOLWATER_ILL3*(MW_WATER/MW)*1e-6
  SOLBILE_CECUM    =(exp(LOGSR))*SOLWATER_CECUM*(MW_WATER/MW)*1e-6
  SOLBILE_ASCENDING=(exp(LOGSR))*SOLWATER_ASCENDING*(MW_WATER/MW)*1e-6
  ENDIF

  !Intestinal fluid solubility (SOLIF) in each compartment Bile Con in mM FROM
GASTROPLUS, WHICH IS FROM porter 2007
  CONSTANT BILE_STOMACH = 0
  CONSTANT BILE_DUO     = 2.8
  CONSTANT BILE_JEJ1    = 2.33
  CONSTANT BILE_JEJ2    = 2.03
  CONSTANT BILE_ILL1    = 1.41
  CONSTANT BILE_ILL2    = 1.16
  CONSTANT BILE_ILL3    = 0.14
  CONSTANT BILE_CECUM   = 0
  CONSTANT BILE_ASCENDING = 0
```

```
  !!Ref: estimation of the increase in solubility of drugs as a function of
bile salt concentrations.
  SOLIF_STOMACH   =   (SOLWATER_STOMACH+SOLBILE_STOMACH*MW*BILE_STOMACH)*0.5
  SOLIF_DUO       =   (SOLWATER_DUO+SOLBILE_DUO*MW*BILE_DUO)*0.5
  SOLIF_JEJ1      =   (SOLWATER_JEJ1+SOLBILE_JEJ1*MW*BILE_JEJ1)*0.5
  SOLIF_JEJ2      =   (SOLWATER_JEJ2+SOLBILE_JEJ2*MW*BILE_JEJ2)*0.5
  SOLIF_ILL1      =   (SOLWATER_ILL1+SOLBILE_ILL1*MW*BILE_ILL1)*0.5
  SOLIF_ILL2      =   (SOLWATER_ILL2+SOLBILE_ILL2*MW*BILE_ILL2)*0.5
  SOLIF_ILL3      =   (SOLWATER_ILL3+SOLBILE_ILL3*MW*BILE_ILL3)*0.5
  SOLIF_CECUM     =   (SOLWATER_CECUM+SOLBILE_CECUM*MW*BILE_CECUM)*0.5
  SOLIF_ASCENDING =
(SOLWATER_ASCENDING+SOLBILE_ASCENDING*MW*BILE_ASCENDING)*0.5

  !Permeability estimation  (used the last one for PAMPA in this model)
  !Roche eqn.; HPeff_est = (10^( 0.892 * log(Caco2/1000000) + 0.626 ))
  !GastroPlus eqn.; HPeff_est = (10^( 0.5326 * log(Caco2/100) + 0.9028 ))*1e-4
  !Yu et al (2000) eqn.; HPeff_est = (10^( 0.6532 * log(Caco2) -0.3036 ))*1e-4
  !Di 2013 for MDCK cells
  !Hpeff in 10-4 cm/s
  !Hpeff experimentally determined or provided by the user in the form of 1
not 1e10-4 cm/s
  CONSTANT HPeff_exp = 1.0
  CONSTANT HPeff_exp_cecum = 1.0
  CONSTANT HPeff_exp_ascending = 1.0
  ! Scaling values for calibrating Caco2 Peff
  ! Reference compound
  ! None        = 0,
  ! Atenolol    = 1
  ! Metoprolol  = 2
  ! Propranolol = 3
  ! Cimetidine  = 4
  ! Midazolam   = 5
  ! Verapamil   = 6

  integer  CACO2PEFF_REFERENCE_COMPOUND
  constant CACO2PEFF_REFERENCE_COMPOUND    = 0

  constant CACO2PEFF_ATENOLOL_PUBLISHED    = 3.06
  constant CACO2PEFF_METOPROLOL_PUBLISHED  = 0.19
  constant CACO2PEFF_PROPRANOLOL_PUBLISHED = 21.15
  constant CACO2PEFF_CIMETIDINE_PUBLISHED  = 20.06
  constant CACO2PEFF_MIDAZOLAM_PUBLISHED   = 75.3
  constant CACO2PEFF_VERAPAMIL_PUBLISHED   = 18.8

  constant CACO2PEFF_ATENOLOL_MEASURED     = 3.06
  constant CACO2PEFF_METOPROLOL_MEASURED   = 0.19
  constant CACO2PEFF_PROPRANOLOL_MEASURED  = 21.15
  constant CACO2PEFF_CIMETIDINE_MEASURED   = 20.06
  constant CACO2PEFF_MIDAZOLAM_MEASURED    = 75.3
  constant CACO2PEFF_VERAPAMIL_MEASURED    = 18.8

  if(CACO2PEFF_REFERENCE_COMPOUND .EQ. 1) then
    SCALAR_PEFF = CACO2PEFF_ATENOLOL_PUBLISHED/CACO2PEFF_ATENOLOL_MEASURED
  else if(CACO2PEFF_REFERENCE_COMPOUND .EQ. 2) then
    SCALAR_PEFF = CACO2PEFF_METOPROLOL_PUBLISHED/CACO2PEFF_METOPROLOL_MEASURED
  else if(CACO2PEFF_REFERENCE_COMPOUND .EQ. 3) then
    SCALAR_PEFF =
CACO2PEFF_PROPRANOLOL_PUBLISHED/CACO2PEFF_PROPRANOLOL_MEASURED
  else if(CACO2PEFF_REFERENCE_COMPOUND .EQ. 4) then
    SCALAR_PEFF = CACO2PEFF_CIMETIDINE_PUBLISHED/CACO2PEFF_CIMETIDINE_MEASURED
  else if(CACO2PEFF_REFERENCE_COMPOUND .EQ. 5) then
    SCALAR_PEFF = CACO2PEFF_MIDAZOLAM_PUBLISHED/CACO2PEFF_MIDAZOLAM_MEASURED
  else if(CACO2PEFF_REFERENCE_COMPOUND .EQ. 6) then
    SCALAR_PEFF = CACO2PEFF_VERAPAMIL_PUBLISHED/CACO2PEFF_VERAPAMIL_MEASURED
  else
```

```
    SCALAR_PEFF = 1.0
  endif

  CACO2PEFF_CALIBRATED           = CACO2PEFF*SCALAR_PEFF
  CACO2PEFF_CALIBRATED_cecum     = CACO2PEFF_cecum*SCALAR_PEFF
  CACO2PEFF_CALIBRATED_ascending = CACO2PEFF_ascending*SCALAR_PEFF

  !Hpeff estimated from Caco2 permeability data using three equations
  !Caco2Peff in 10-6 cm/s, which corresponds to Caco2 in the user interface
  !Input Caco2peff is in the form of 1 not 1e-6 cm/s
  integer HPEFF_METHOD
  constant HPEFF_METHOD =4 ! 1 = Roche, 2 = Gastro-Plus, 3 = Yu, et al, 4
=equation for MDCK, 5= experimental
  if(HPEFF_METHOD .eq. 1) then
    HPeff_est             = 10**(0.892 * log10(CACO2PEFF_CALIBRATED/1e6) +
0.626)  ! unit cm/s
    HPeff_est_cecum       = 10**(0.892 * log10(CACO2PEFF_CALIBRATED_cecum/1e6)
+ 0.626)
    HPeff_est_ascending   = 10**(0.892 *
log10(CACO2PEFF_CALIBRATED_ascending/1e6) + 0.626)
  else if(HPEFF_METHOD .eq. 2) then
    HPeff_est             = 10**(0.5326 * log10(CACO2PEFF_CALIBRATED/100) +
0.9028)*1e-4
    HPeff_est_cecum       = 10**(0.5326 *
log10(CACO2PEFF_CALIBRATED_cecum/100) + 0.9028)*1e-4
    HPeff_est_ascending   = 10**(0.5326 *
log10(CACO2PEFF_CALIBRATED_ascending/100) + 0.9028)*1e-4
  else if(HPEFF_METHOD .eq. 3) then
    HPeff_est             = 10**(0.6532 * log10(CACO2PEFF_CALIBRATED) -
0.3036)*1e-4
    HPeff_est_cecum       = 10**(0.6532 * log10(CACO2PEFF_CALIBRATED_cecum) -
0.3036)*1e-4
    HPeff_est_ascending   = 10**(0.6532 *
log10(CACO2PEFF_CALIBRATED_ascending) - 0.3036)*1e-4
  else if(HPEFF_METHOD .eq. 4) then
    HPeff_est             = 10**(0.92 * log10(CACO2PEFF_CALIBRATED/1e6) +0.86)
    HPeff_est_cecum       = 10**(0.92* log10(CACO2PEFF_CALIBRATED_cecum/1e6)
+0.86)
    HPeff_est_ascending   = 10**(0.92*
log10(CACO2PEFF_CALIBRATED_ascending/1e6) +0.86)
  else
    ! experimental
    HPeff_est             = HPeff_exp*1e-4
    HPeff_est_cecum       = HPeff_exp_cecum*1e-4
    HPeff_est_ascending   = HPeff_exp_ascending*1e-4
  endif

  !Estimation of diffusion coefficient
  !Drug diffusion velocity through the gut membrane, DIFF, in cm3/h; ml/h
  !Exchange surface area in cm^2
  !Hpeff is in cm/s
  !Rate in ml/h, apical to basolateral, apparent diffusion velocity (Diff)
cm3/h
  DIFF_duo              = 60*60 *HPeff_est*ESA_duo
  DIFF_JEJ1             = 60*60*HPeff_est*ESA_JEJ1
  DIFF_JEJ2             = 60*60*HPeff_est*ESA_JEJ2
  DIFF_ILL1             = 60*60*HPeff_est*ESA_ILL1
  DIFF_ILL2             = 60*60*HPeff_est*ESA_ILL2
  DIFF_ILL3             = 60*60*HPeff_est*ESA_ILL3
  DIFF_cecum            = 60*60*HPeff_est_cecum*ESA_cecum
  DIFF_ascending        = 60*60*HPeff_est_ascending*ESA_ascending

  !UNIONIZED FRACTION (NI) in each gut compartment
  NI_DUO                = 1/HHDUO
  NI_JEJ1               = 1/HHJEJ1
  NI_JEJ2               = 1/HHJEJ2
```

```
  NI_ILL1                 = 1/HHILL1
  NI_ILL2                 = 1/HHILL2
  NI_ILL3                 = 1/HHILL3
  NI_CECUM                = 1/HHCECUM
  NI_ASCENDING            = 1/HHASCENDING

  !!!!!!!!!!!!!!!!!!!!!!!!!!!!!!!!!ACAT
MODEL!!!!!!!!!!!!!!!!!!!!!!!!!!!!!!!!!!!!!!!!!!

  !Scaled dosing parameters for iv dosing (Not used)

    IVdosed               = IVdoseCd*BW           !(ug)|intravenous dose of
d-MPH
    IVdosel               = IVdoseCl*BW           !(ug)|intravenous dose of
l-MPH
    IVd                   = (IVdosed)/IVtime      !(ug/h)|intravenous dosing
rate of d-MPH
    IVl                   = (IVdosel)/IVtime      !(ug/h)|intravenous dosing
rate of l-MPH
    IVZONE                = RSW(T.GT.IVtime,0.,1.)!(no units)|real (floating
point) switch
    IVrd                  = IVd*IVZONE            !(ug/h)|Scheduled
intravenous dosing of d-MPH
    IVrl                  = IVl*IVZONE            !(ug/h)|Scheduled
intravenous dosing of l-MPH
    AIVd                  = integ(IVrd,0.0)       !(ug)|Amount of d-MPH
received by i.v.
    AIVl                  = integ(IVrl,0.0)       !(ug)|Amount of l-MPH
received by i.v.


  !1cm = 10000um
  !Dissolution diffusion layer model Noyes-Whitney equation, ref: Predicting
Pharmacokinetics of Drugs Using Physiologically Based Modeling???Application
to Food Effects Parrott 2009
  !Dissolution rate constants: should be in the units of L/(mg*hr)
   Kd_IR                  = 3*(DIFFCOEFF_IR/(rho*rparticle*DLT))*60*100

  !!Absorption model
  !C in the lumen in ug/ml, C in the enterocytes in ug/L; SOL in mg/l which
equals ug/ml, DIFF in ml/h, Qmuc in L/h, V for lumen in ml, V for enterocytes
L; T in hr, X in ug
  !Amount in stomach in ug

  !!!%%%%%%%%%%%%%%%%%%%%%%%%%%%%%%%%%%%%%%%%%%%%%%FOR d-MPH
%%%%%%%%%%%%%%%%%%%%%%%%%%%%%%%%%%%%%%%%%%%

!STOMACH

!Lumen solid

X_released_DMPHER_stomach       = integ(D_released_DMPHER_stomach,0.0)
                     !Amount of ER d-MPH released in the stomach
D_released_DMPHER_stomach       = RELEASE_stomach
                     !Rate of ER d-MPH release in the stomach
D_X_STOMACH_FORM_DT_DMPHER      = -D_released_DMPHER_stomach
-X_STOMACH_FORM_DR_DMPHER/Tstomach     !Rate of ER d-MPH (solid) amount change
in the stomach
X_STOMACH_FORM_DR_DMPHER        = INTEG(D_X_STOMACH_FORM_DT_DMPHER, Adoseder)
                     !Amount of ER d-MPH (solid) in the stomach
C_STOMACH_FORM_DMPHER           = X_STOMACH_FORM_DR_DMPHER/Vstomach
                     !Concentration of ER d-MPH (solid) in the stomach

D_X_STOMACH_SOLID_DT_DMPHIR     =
-VSTOMACH*Kd_IR*C_STOMACH_SOLID_DMPHIR*(SOLIF_STOMACH-C_STOMACH_DISS_DMPH)-X_S
TOMACH_SOLID_DMPHIR/Tstomach !Rate of IR d-MPH solid amount change in the
```

```
stomach
X_STOMACH_SOLID_DMPHIR          = BOUND(0.0,ADOSEDIR,
LIMINT(D_X_STOMACH_SOLID_DT_DMPHIR,ADOSEDIR, 0.0, ADOSEDIR))
        !Amount of IR d-MPH solid in the stomach
C_STOMACH_SOLID_DMPHIR          = X_STOMACH_SOLID_DMPHIR/VSTOMACH
                                                              !Concentration
of IR d-MPH solid in the stomach
ADOSED                          = ADOSEDIR+ADOSEDER
                                                              !Total dose of
d-MPH

D_dissolve_solid_DIR_STOMACH    =
VSTOMACH*Kd_IR*C_STOMACH_SOLID_DMPHIR*(SOLIF_STOMACH-C_STOMACH_DISS_DMPH)
                            !Rate of IR d-MPH (solid) dissolution in the
stomach
X_dissolve_solid_DIR_STOMACH    = integ(D_dissolve_solid_DIR_STOMACH, 0.0)
                                                              !Amount of IR
d-MPH (solid) dissolved in the stomach

!!Lumen dissolved
D_X_STOMACH_DISS_DT_DMPH        = D_released_DMPHER_stomach+
VSTOMACH*Kd_IR*C_STOMACH_SOLID_DMPHIR*(SOLIF_STOMACH-C_STOMACH_DISS_DMPH)-X_ST
OMACH_DISS_DMPH/Tstomach   !Rate of  amount change of dissolved IR and ER
d-MPH in the stomach
X_STOMACH_DISS_DMPH             = integ(D_X_STOMACH_DISS_DT_DMPH,0.0)

        !Amount of IR and ER d-MPH dissolved in the stomach
C_STOMACH_DISS_DMPH             = X_STOMACH_DISS_DMPH/VSTOMACH

        !Concentration of IR and ER d-MPH dissolived in the stomach

!DUODENUM
!Lumen_solid

CONSTANT c = 4.0
CONSTANT a = 0.001
CONSTANT b = 10

If (t .LE. c) Then                    !release rate of ER forms
Release_duo           =  0.
Release_jej1          =  0.
Release_jej2          =  0.
Release_ill1          =  0.
Release_ill2          =  0.
Release_ill3          =  0.
Release_cecum         =  0.
Release_ascending     =  0.
RELEASE_stomach       =  0.
else
Release_duo           =
-X_duo_FORM_DMPHER*(exp(-((t-c)**b)/a))*(-b/a)*((t-c)**(b-1))
Release_jej1          =
-X_jej1_FORM_DMPHER*(exp(-((t-c)**b)/a))*(-b/a)*((t-c)**(b-1))
Release_jej2          =
-X_jej2_FORM_DMPHER*(exp(-((t-c)**b)/a))*(-b/a)*((t-c)**(b-1))
Release_ill1          =
-X_ill1_FORM_DMPHER*(exp(-((t-c)**b)/a))*(-b/a)*((t-c)**(b-1))
Release_ill2          =
-X_ill2_FORM_DMPHER*(exp(-((t-c)**b)/a))*(-b/a)*((t-c)**(b-1))
Release_ill3          =
-X_ill3_FORM_DMPHER*(exp(-((t-c)**b)/a))*(-b/a)*((t-c)**(b-1))
Release_cecum         =
-X_cecum_FORM_DMPHER*(exp(-((t-c)**b)/a))*(-b/a)*((t-c)**(b-1))
Release_ascending     =
-X_ascending_FORM_DMPHER*(exp(-((t-c)**b)/a))*(-b/a)*((t-c)**(b-1))
RELEASE_stomach       =  0.
```

```
Endif

totalreleaserate      =
Release_duo+Release_jej1+Release_jej2+Release_ill1+Release_ill2+Release_ill3+R
elease_cecum+Release_ascending
totalreleaseF         =
(X_released_DMPHER_DUO+X_released_DMPHER_jej1+X_released_DMPHER_jej2+X_release
d_DMPHER_ill1+X_released_DMPHER_ill2+X_released_DMPHER_ill3+X_released_DMPHER_
cecum+X_released_DMPHER_ascending) /(Adoseder+1e-34)

X_released_DMPHER_DUO           = integ(D_released_DMPHER_DUO,0.0)
D_released_DMPHER_DUO           = release_duo
D_duo_FORM_DMPHER               = -D_released_DMPHER_DUO +
X_STOMACH_FORM_DR_DMPHER/Tstomach -(X_duo_FORM_DMPHER/TDUO)
X_duo_FORM_DMPHER               = integ(D_duo_FORM_DMPHER, 0.0)
C_DUO_FORM_DMPHER               = X_duo_FORM_DMPHER/VDUO

D_X_DUO_SOLID_DT_DMPHIR         =
-VDUO*Kd_IR*C_DUO_SOLID_DMPHIR*(SOLIF_DUO-C_DUO_DISS_DMPH)+X_STOMACH_SOLID_DMP
HIR/Tstomach -(X_DUO_SOLID_DMPHIR/TDUO)
X_DUO_SOLID_DMPHIR              = BOUND(0.0,
ADOSEDIR,LIMINT(D_X_DUO_SOLID_DT_DMPHIR,0.0, 0.0, ADOSEDIR))
C_DUO_SOLID_DMPHIR              = X_DUO_SOLID_DMPHIR/VDUO

D_dissolve_solid_dir_duo        =
VDUO*Kd_IR*C_DUO_SOLID_DMPHIR*(SOLIF_DUO-C_DUO_DISS_DMPH)
X_dissolve_solid_dir_duo        = integ(D_dissolve_solid_DIR_duo, 0.0)

!Lumen_dissolved
D_X_DUO_DISS_DT_DMPH              =
VDUO*Kd_IR*C_DUO_SOLID_DMPHIR*(SOLIF_DUO-C_DUO_DISS_DMPH)+D_released_DMPHER_DU
O-NI_DUO*DIFF_DUO*(-C_DUO_MEM_DMPH/1000+C_DUO_DISS_DMPH)+X_STOMACH_DISS_DMPH/T
stomach-X_DUO_DISS_DMPH/TDUO
X_DUO_DISS_DMPH                 = INTEG(D_X_DUO_DISS_DT_DMPH, 0.0)
C_DUO_DISS_DMPH                 = X_DUO_DISS_DMPH/VDUO

!Enterocytes
D_MEM_DUO_DT_DMPH               =
QDUO*CAD-QDUO*(MEM_DUO_DMPH/(VMEMDUO*KpGut))*1 - METABOLISM_DUO_DMPH +
NI_DUO*DIFF_DUO*(-C_DUO_MEM_DMPH/1000+C_DUO_DISS_DMPH) ! - EFFLUX_DUO +
INFLUX_DUO  !ug/hr !Rate of d-MPH amount change in enterocytes
MEM_DUO_DMPH                    = INTEG(D_MEM_DUO_DT_DMPH,0.0)           !!ug
 Amount of d-MPH in enterocytes
C_DUO_MEM_DMPH                  = MEM_DUO_DMPH/VMEMDUO
!!ug/L Concentration of d-MPH in enterocytes
R_MEM_DUO_DMPH                  = QDUO*(MEM_DUO_DMPH/(VMEMDUO*KpGut))*1
!!ug/hr  Rate of d-MPH entering portal vein and the liver
A_MEM_DUO_DMPH                  = integ(R_MEM_DUO_DMPH, 0.0)             !!ug
Amount of d-MPH entering portal vein and the liver
METABOLISM_DUO_DMPH             = K5D*MEM_DUO_DMPH
!!ug/hr Rate of d-MPH metabolism in enterocytes
AMETABOLISM_DUO_DMPH            = INTEG(METABOLISM_DUO_DMPH, 0.0)        !!ug
Amount of d-MPH metaboism in enterocytes

D_ABS_DUO_DMPH                  =
NI_DUO*DIFF_DUO*(-C_DUO_MEM_DMPH/1000+C_DUO_DISS_DMPH)     !!Net rate of d-MPH
entering enterocytes from intestinal lumen
X_ABS_DUO_DMPH                  = INTEG(D_ABS_DUO_DMPH, 0.0)
              !!Net amount of d-MPH entering enterocytes from intestinal lumen

D_FG_DUO_DMPH                   =
-QDUO*CAD+QDUO*(MEM_DUO_DMPH/(VMEMDUO*KpGut))*1            !!Net rate of d-MPH
entering blood circulation
X_FG_DUO_DMPH                   = INTEG(D_FG_DUO_DMPH, 0.0)
              !!Net amount of d-MPH entering blood circulation
```

```
Da =
(X_ABS_DUO_DMPH+X_ABS_jej1_DMPH+X_ABS_jej2_DMPH+X_ABS_ill1_DMPH+X_ABS_ill2_DMP
H+X_ABS_ill3_DMPH+X_ABS_cecum_DMPH+X_ABS_ascending_DMPH) !! Total amount of
d-MPH entering enterocytes from intestinal lumen
La =
(X_ABS_DUO_lMPH+X_ABS_jej1_lMPH+X_ABS_jej2_lMPH+X_ABS_ill1_lMPH+X_ABS_ill2_lMP
H+X_ABS_ill3_lMPH+X_ABS_cecum_lMPH+X_ABS_ascending_lMPH) !! Total amount of
l-MPH entering enterocytes from intestinal lumen
Fa = (Da+La)/(AdosedER+AdosedIR+AdoseLER+AdoseLIR+1e-34)
                                                              !! Fraction of
total MPH absorbed

!JEJUNUM1
!Lumen solid
X_released_DMPHER_JEJ1         = integ(D_released_DMPHER_JEJ1,0.0)  !! ug
D_released_DMPHER_JEJ1         = release_jej1
D_JEJ1_FORM_DMPHER             = -D_released_DMPHER_JEJ1
-X_JEJ1_FORM_DMPHER/TJEJ1  +X_DUO_FORM_DMPHER/TDUO
X_JEJ1_FORM_DMPHER             = integ(D_JEJ1_FORM_DMPHER, 0.0)  !! ug
C_JEJ1_FORM_DMPHER             = X_JEJ1_FORM_DMPHER/VJEJ1       !! ug/mL

D_X_JEJ1_SOLID_DT_DMPHIR       =
-Vjej1*Kd_IR*C_JEJ1_SOLID_DMPHIR*(SOLIF_JEJ1-C_JEJ1_DISS_DMPH)-(X_JEJ1_SOLID_D
MPHIR/TJEJ1) + X_DUO_SOLID_DMPHIR/TDUO
X_JEJ1_SOLID_DMPHIR            = BOUND(0.0,
ADOSEDIR,LIMINT(D_X_JEJ1_SOLID_DT_DMPHIR,0.0, 0.0,ADOSEDIR))    !ug
C_JEJ1_SOLID_DMPHIR            = X_JEJ1_SOLID_DMPHIR/VJEJ1

!Lumen_dissolived
D_X_JEJ1_DISS_DT_DMPH           =
VJEJ1*Kd_IR*C_JEJ1_SOLID_DMPHIR*(SOLIF_JEJ1-C_JEJ1_DISS_DMPH)+D_released_DMPHE
R_JEJ1-NI_JEJ1*DIFF_JEJ1*(-C_JEJ1_MEM_DMPH/1000+C_JEJ1_DISS_DMPH)
-X_JEJ1_DISS_DMPH/TJEJ1+X_DUO_DISS_DMPH/TDUO
X_JEJ1_DISS_DMPH               = INTEG(D_X_JEJ1_DISS_DT_DMPH, 0.0)
C_JEJ1_DISS_DMPH               = X_JEJ1_DISS_DMPH/VJEJ1

D_dissolve_solid_dir_jej1      =
VJEJ1*Kd_IR*C_JEJ1_SOLID_DMPHIR*(SOLIF_JEJ1-C_JEJ1_DISS_DMPH)
X_dissolve_solid_dir_jej1      = integ(D_dissolve_solid_dir_jej1, 0.0)

!Enterocytes
D_MEM_JEJ1_DT_DMPH             =
QJEJ1*CAD-QJEJ1*(MEM_JEJ1_DMPH/(VMEMJEJ1*KpGut))*1 - METABOLISM_JEJ1_DMPH +
NI_JEJ1*DIFF_JEJ1*(-C_JEJ1_MEM_DMPH/1000+C_JEJ1_DISS_DMPH) ! - EFFLUX_DUO +
INFLUX_DUO  !ug/hr
MEM_JEJ1_DMPH                  = INTEG(D_MEM_JEJ1_DT_DMPH,0.0)  !!ug
C_JEJ1_MEM_DMPH                = MEM_JEJ1_DMPH/VMEMJEJ1          !!ug/L
R_MEM_JEJ1_DMPH                = QJEJ1*(MEM_JEJ1_DMPH/(VMEMJEJ1*KpGut))*1
!!ug/hr
A_MEM_JEJ1_DMPH                = integ(R_MEM_JEJ1_DMPH, 0.0)    !!ug
METABOLISM_JEJ1_DMPH           = K5D*MEM_JEJ1_DMPH            !!ug/hr
AMETABOLISM_JEJ1_DMPH          = INTEG(METABOLISM_JEJ1_DMPH, 0.0)  !!ug

D_ABS_JEJ1_DMPH                =
NI_JEJ1*DIFF_JEJ1*(-C_JEJ1_MEM_DMPH/1000+C_JEJ1_DISS_DMPH)
X_ABS_JEJ1_DMPH                = INTEG(D_ABS_JEJ1_DMPH, 0.0)

D_FG_JEJ1_DMPH                 =
-QJEJ1*CAD+QJEJ1*(MEM_JEJ1_DMPH/(VMEMJEJ1*KpGut))*1
X_FG_JEJ1_DMPH                 = INTEG(D_FG_JEJ1_DMPH, 0.0)

!JEJUNUM2
!Lumen solid
X_released_DMPHER_JEJ2         = integ(D_released_DMPHER_JEJ2,0.0)  !! ug
D_released_DMPHER_JEJ2         = release_jej2
D_JEJ2_FORM_DMPHER             = -D_released_DMPHER_JEJ2
```

```
+X_JEJ1_FORM_DMPHER/TJEJ1 -X_JEJ2_FORM_DMPHER/TJEJ2
X_JEJ2_FORM_DMPHER             = integ(D_JEJ2_FORM_DMPHER, 0.0)  !! ug
C_JEJ2_FORM_DMPHER             = X_JEJ2_FORM_DMPHER/VJEJ2       !! ug/mL

D_X_JEJ2_SOLID_DT_DMPHIR       =
-VJEJ2*Kd_IR*C_JEJ2_SOLID_DMPHIR*(SOLIF_JEJ2-C_JEJ2_DISS_DMPH) +
X_JEJ1_SOLID_DMPHIR/TJEJ1-(X_JEJ2_SOLID_DMPHIR/TJEJ2)              !
X_JEJ2_SOLID_DMPHIR            = BOUND(0.0,
ADOSEDIR,LIMINT(D_X_JEJ2_SOLID_DT_DMPHIR,0.0, 0.0, ADOSEDIR))    !ug!
C_JEJ2_SOLID_DMPHIR            = X_JEJ2_SOLID_DMPHIR/VJEJ2

!Lumen_dissolived
D_X_JEJ2_DISS_DT_DMPH           =
VJEJ2*Kd_IR*C_JEJ2_SOLID_DMPHIR*(SOLIF_JEJ2-C_JEJ2_DISS_DMPH)+
D_released_DMPHER_JEJ2-NI_JEJ2*DIFF_JEJ2*(-C_JEJ2_MEM_DMPH/1000+C_JEJ2_DISS_DM
PH)  +X_JEJ1_DISS_DMPH/TJEJ1 -X_JEJ2_DISS_DMPH/TJEJ2    !
X_JEJ2_DISS_DMPH               = INTEG(D_X_JEJ2_DISS_DT_DMPH,0.0)!
C_JEJ2_DISS_DMPH               = X_JEJ2_DISS_DMPH/VJEJ2

D_dissolve_solid_dir_jej2     =
VJEJ2*Kd_IR*C_JEJ2_SOLID_DMPHIR*(SOLIF_JEJ2-C_JEJ2_DISS_DMPH)
x_dissolve_solid_dir_jej2     = INTEG(D_dissolve_solid_dir_jej2, 0.0)

!Enterocytes
D_MEM_JEJ2_DT_DMPH            =
QJEJ2*CAD-QJEJ2*(MEM_JEJ2_DMPH/(VMEMJEJ2*KpGut))*1 - METABOLISM_JEJ2_DMPH
+NI_JEJ2*DIFF_JEJ2*(-C_JEJ2_MEM_DMPH/1000+C_JEJ2_DISS_DMPH) ! - EFFLUX_JEJ1 +
INFLUX_JEJ1  !ug/hr
MEM_JEJ2_DMPH                 = INTEG(D_MEM_JEJ2_DT_DMPH,0.0)  !!ug
C_JEJ2_MEM_DMPH               = MEM_JEJ2_DMPH/VMEMJEJ2          !!ug/L
R_MEM_JEJ2_DMPH               = QJEJ2*(MEM_JEJ2_DMPH/(VMEMJEJ2*KpGut))*1
!!ug/hr
A_MEM_JEJ2_DMPH               = integ(R_MEM_JEJ2_DMPH, 0.0)    !!ug
METABOLISM_JEJ2_DMPH          = K5D*MEM_JEJ2_DMPH            !!ug/hr
AMETABOLISM_JEJ2_DMPH         = INTEG(METABOLISM_JEJ2_DMPH, 0.0)  !!ug

D_ABS_JEJ2_DMPH               =
NI_JEJ2*DIFF_JEJ2*(-C_JEJ2_MEM_DMPH/1000+C_JEJ2_DISS_DMPH)
X_ABS_JEJ2_DMPH               = INTEG(D_ABS_JEJ2_DMPH, 0.0)

D_FG_JEJ2_DMPH                =
-QJEJ2*CAD+QJEJ2*(MEM_JEJ2_DMPH/(VMEMJEJ2*KpGut))*1
X_FG_JEJ2_DMPH                = INTEG(D_FG_JEJ2_DMPH, 0.0)

!ILLEUM1
!Lumen solid
X_released_DMPHER_ILL1        = integ(D_released_DMPHER_ILL1,0.0)  !! ug
D_released_DMPHER_ILL1        = release_ill1
D_ILL1_FORM_DMPHER            = -D_released_DMPHER_ILL1
+X_JEJ2_FORM_DMPHER/TJEJ2  -X_ILL1_FORM_DMPHER/TILL1
X_ILL1_FORM_DMPHER            = integ(D_ILL1_FORM_DMPHER, 0.0)  !! ug
C_ILL1_FORM_DMPHER            = X_ILL1_FORM_DMPHER/VILL1       !! ug/mL

D_X_ILL1_SOLID_DT_DMPHIR      =
-VILL1*Kd_IR*C_ILL1_SOLID_DMPHIR*(SOLIF_ILL1-C_ILL1_DISS_DMPH)+X_JEJ2_SOLID_DM
PHIR/TJEJ2  -(X_ILL1_SOLID_DMPHIR/TILL1)                     !
X_ILL1_SOLID_DMPHIR           = BOUND(0.0,
ADOSEDIR,LIMINT(D_X_ILL1_SOLID_DT_DMPHIR,0.0, 0.0, ADOSEDIR))    !ug
C_ILL1_SOLID_DMPHIR           = X_ILL1_SOLID_DMPHIR/VILL1

!Lumen_dissolived
D_X_ILL1_DISS_DT_DMPH          =
VILL1*Kd_IR*C_ILL1_SOLID_DMPHIR*(SOLIF_ILL1-C_ILL1_DISS_DMPH)+D_released_DMPHE
R_ILL1-NI_ILL1*DIFF_ILL1*(-C_ILL1_MEM_DMPH/1000+C_ILL1_DISS_DMPH) +
X_JEJ2_DISS_DMPH/TJEJ2-X_ill1_DISS_DMPH/TILL1
X_ILL1_DISS_DMPH              = INTEG(D_X_ILL1_DISS_DT_DMPH,0.0)
```

```
C_ILL1_DISS_DMPH              = X_ILL1_DISS_DMPH/VILL1

D_dissolve_solid_dir_ILL1     =
VILL1*Kd_IR*C_ILL1_SOLID_DMPHIR*(SOLIF_ILL1-C_ILL1_DISS_DMPH)
X_dissolve_solid_dir_ILL1     = INTEG(D_dissolve_solid_dir_ILL1, 0.0)

!Enterocytes
D_MEM_ILL1_DT_DMPH            =
QILL1*CAD-QILL1*(MEM_ILL1_DMPH/(VMEMILL1*KpGut))*1 - METABOLISM_ILL1_DMPH
+NI_ILL1*DIFF_ILL1*(-C_ILL1_MEM_DMPH/1000+C_ILL1_DISS_DMPH) ! - EFFLUX_DUO +
INFLUX_DUO  !ug/hr
MEM_ILL1_DMPH                 = INTEG(D_MEM_ILL1_DT_DMPH,0.0)  !!ug
C_ILL1_MEM_DMPH               = MEM_ILL1_DMPH/VMEMILL1          !!ug/L
R_MEM_ILL1_DMPH               = QILL1*(MEM_ILL1_DMPH/(VMEMILL1*KpGut))*1
!!ug/hr
A_MEM_ILL1_DMPH               = integ(R_MEM_ILL1_DMPH, 0.0)    !!ug
METABOLISM_ILL1_DMPH          = K5D*MEM_ILL1_DMPH            !!ug/hr
AMETABOLISM_ILL1_DMPH         = INTEG(METABOLISM_ILL1_DMPH, 0.0)  !!ug

D_ABS_ILL1_DMPH               =
NI_ILL1*DIFF_ILL1*(-C_ILL1_MEM_DMPH/1000+C_ILL1_DISS_DMPH)
X_ABS_ILL1_DMPH               = INTEG(D_ABS_ILL1_DMPH, 0.0)

D_FG_ILL1_DMPH                =
-QILL1*CAD+QILL1*(MEM_ILL1_DMPH/(VMEMILL1*KpGut))*1
X_FG_ILL1_DMPH                = INTEG(D_FG_ILL1_DMPH, 0.0)

!ILLEUM2
!Lumen
X_released_DMPHER_ILL2        = integ(D_released_DMPHER_ILL2,0.0)  !! ug
D_released_DMPHER_ILL2        = release_ill2
D_ILL2_FORM_DMPHER            = -D_released_DMPHER_ILL2  +
X_ILL1_FORM_DMPHER/TILL1-X_ILL2_FORM_DMPHER/TILL2
X_ILL2_FORM_DMPHER            = integ(D_ILL2_FORM_DMPHER, 0.0)  !! ug
C_ILL2_FORM_DMPHER            = X_ILL2_FORM_DMPHER/VILL2       !! ug/mL

D_X_ILL2_SOLID_DT_DMPHIR      =
-VILL2*Kd_IR*C_ILL2_SOLID_DMPHIR*(SOLIF_ILL2-C_ILL2_DISS_DMPH)+
X_ILL1_SOLID_DMPHIR/TILL1   -(X_ILL2_SOLID_DMPHIR/TILL2)
X_ILL2_SOLID_DMPHIR           = BOUND(0.0,
ADOSEDIR,LIMINT(D_X_ILL2_SOLID_DT_DMPHIR,0.0, 0.0, ADOSEDIR))    !ug
C_ILL2_SOLID_DMPHIR           = X_ILL2_SOLID_DMPHIR/VILL2

!Lumen_dissolived
D_X_ILL2_DISS_DT_DMPH          =
VILL2*Kd_IR*C_ILL2_SOLID_DMPHIR*(SOLIF_ILL2-C_ILL2_DISS_DMPH)+D_released_DMPHE
R_ILL2-NI_ILL2*DIFF_ILL2*(-C_ILL2_MEM_DMPH/1000+C_ILL2_DISS_DMPH)
+X_ILL1_DISS_DMPH/TILL1-X_ILL2_DISS_DMPH/TILL2
X_ILL2_DISS_DMPH              = INTEG(D_X_ILL2_DISS_DT_DMPH, 0.0)
C_ILL2_DISS_DMPH              = X_ILL2_DISS_DMPH/VILL2

D_dissolve_solid_dir_ILL2     =
VILL2*Kd_IR*C_ILL2_SOLID_DMPHIR*(SOLIF_ILL2-C_ILL2_DISS_DMPH)
x_dissolve_solid_dir_ILL2     = integ(D_dissolve_solid_dir_ILL2, 0.0)

!Enterocytes
D_MEM_ILL2_DT_DMPH            =
QILL2*CAD-QILL2*(MEM_ILL2_DMPH/(VMEMILL2*KpGut))*1 - METABOLISM_ILL2_DMPH
+NI_ILL2*DIFF_ILL2*(-C_ILL2_MEM_DMPH/1000+C_ILL2_DISS_DMPH) ! - EFFLUX_DUO +
INFLUX_DUO  !ug/hr
MEM_ILL2_DMPH                 = INTEG(D_MEM_ILL2_DT_DMPH,0.0)  !!ug
C_ILL2_MEM_DMPH               = MEM_ILL2_DMPH/VMEMILL2          !!ug/L
R_MEM_ILL2_DMPH               = QILL2*(MEM_ILL2_DMPH/(VMEMILL2*KpGut))*1
!!ug/hr
A_MEM_ILL2_DMPH               = integ(R_MEM_ILL2_DMPH, 0.0)    !!ug
METABOLISM_ILL2_DMPH          = K5D*MEM_ILL2_DMPH            !!ug/hr
```

```
AMETABOLISM_ILL2_DMPH          = INTEG(METABOLISM_ILL2_DMPH, 0.0)  !!ug

D_ABS_ILL2_DMPH                =
NI_ILL2*DIFF_ILL2*(-C_ILL2_MEM_DMPH/1000+C_ILL2_DISS_DMPH)
X_ABS_ILL2_DMPH                = INTEG(D_ABS_ILL2_DMPH, 0.0)

D_FG_ILL2_DMPH                 =
-QILL2*CAD+QILL2*(MEM_ILL2_DMPH/(VMEMILL2*KpGut))*1
X_FG_ILL2_DMPH                 = INTEG(D_FG_ILL2_DMPH, 0.0)

!ILLEUM3
X_released_DMPHER_ILL3         = integ(D_released_DMPHER_ILL3,0.0)  !! ug
D_released_DMPHER_ILL3         = release_ill3
D_ILL3_FORM_DMPHER             = -D_released_DMPHER_ILL3
+X_ILL2_FORM_DMPHER/TILL2-X_ILL3_FORM_DMPHER/TILL3
X_ILL3_FORM_DMPHER             = integ(D_ILL3_FORM_DMPHER, 0.0)  !! ug
C_ILL3_FORM_DMPHER             = X_ILL3_FORM_DMPHER/VILL3       !! ug/mL

D_X_ILL3_SOLID_DT_DMPHIR       =
-VILL3*Kd_IR*C_ILL3_SOLID_DMPHIR*(SOLIF_ILL3-C_ILL3_DISS_DMPH)+X_ILL2_SOLID_DM
PHIR/TILL2 -(X_ILL3_SOLID_DMPHIR/TILL3)
X_ILL3_SOLID_DMPHIR            = BOUND(0.0,
ADOSEDIR,LIMINT(D_X_ILL3_SOLID_DT_DMPHIR,0.0, 0.0, ADOSEDIR))
C_ILL3_SOLID_DMPHIR            = X_ILL3_SOLID_DMPHIR/VILL3

!Lumen_dissolived
D_X_ILL3_DISS_DT_DMPH           =
VILL3*Kd_IR*C_ILL3_SOLID_DMPHIR*(SOLIF_ILL3-C_ILL3_DISS_DMPH)+D_released_DMPHE
R_ILL3-NI_ILL3*DIFF_ILL3*(-C_ILL3_MEM_DMPH/1000+C_ILL3_DISS_DMPH)+(X_ILL2_DISS
_DMPH/TILL2)-(X_ILL3_DISS_DMPH/TILL3)
X_ILL3_DISS_DMPH               = INTEG(D_X_ILL3_DISS_DT_DMPH, 0.0)
C_ILL3_DISS_DMPH               = X_ILL3_DISS_DMPH/VILL3

D_dissolve_solid_dir_ILL3      =
VILL3*Kd_IR*C_ILL3_SOLID_DMPHIR*(SOLIF_ILL3-C_ILL3_DISS_DMPH)
x_dissolve_solid_dir_ILL3      = integ(D_dissolve_solid_dir_ILL3, 0.0)

!Enterocytes
D_MEM_ILL3_DT_DMPH             =
QILL3*CAD-QILL3*(MEM_ILL3_DMPH/(VMEMILL3*KpGut))*1 - METABOLISM_ILL3_DMPH +
NI_ILL3*DIFF_ILL3*(-C_ILL3_MEM_DMPH/1000+C_ILL3_DISS_DMPH) ! - EFFLUX_DUO +
INFLUX_DUO  !ug/hr
MEM_ILL3_DMPH                  = INTEG(D_MEM_ILL3_DT_DMPH,0.0)  !!ug
C_ILL3_MEM_DMPH                = MEM_ILL3_DMPH/VMEMILL3          !!ug/L
R_MEM_ILL3_DMPH                = QILL3*(MEM_ILL3_DMPH/(VMEMILL3*KpGut))*1
!!ug/hr
A_MEM_ILL3_DMPH                = integ(R_MEM_ILL3_DMPH, 0.0)    !!ug
METABOLISM_ILL3_DMPH           = K5D*MEM_ILL3_DMPH            !!ug/hr
AMETABOLISM_ILL3_DMPH          = INTEG(METABOLISM_ILL3_DMPH, 0.0)  !!ug

D_ABS_ILL3_DMPH                =
NI_ILL3*DIFF_ILL3*(-C_ILL3_MEM_DMPH/1000+C_ILL3_DISS_DMPH)
X_ABS_ILL3_DMPH                = INTEG(D_ABS_ILL3_DMPH, 0.0)

D_FG_ILL3_DMPH                 =
-QILL3*CAD+QILL3*(MEM_ILL3_DMPH/(VMEMILL3*KpGut))*1
X_FG_ILL3_DMPH                 = INTEG(D_FG_ILL3_DMPH, 0.0)

!Cecum
!Lumen SOLID
X_released_DMPHER_CECUM       = integ(D_released_DMPHER_CECUM,0.0)  !! ug
D_released_DMPHER_CECUM       = release_cecum
D_CECUM_FORM_DMPHER           = -D_released_DMPHER_CECUM
+X_ILL3_FORM_DMPHER/TILL3  -X_CECUM_FORM_DMPHER/TCECUM
X_CECUM_FORM_DMPHER           = integ(D_CECUM_FORM_DMPHER, 0.0)  !! ug
C_CECUM_FORM_DMPHER           = X_CECUM_FORM_DMPHER/VCECUM       !! ug/mL
```

```
D_X_CECUM_SOLID_DT_DMPHIR    =
-VCECUM*Kd_IR*C_CECUM_SOLID_DMPHIR*(SOLIF_CECUM-C_CECUM_DISS_DMPH) +
X_ILL3_SOLID_DMPHIR/TILL3-(X_CECUM_SOLID_DMPHIR/TCECUM)!
X_CECUM_SOLID_DMPHIR         = BOUND(0.0,
ADOSEDIR,LIMINT(D_X_CECUM_SOLID_DT_DMPHIR,0.0, 0.0, ADOSEDIR))
C_CECUM_SOLID_DMPHIR         = X_CECUM_SOLID_DMPHIR/VCECUM

!Lumen_dissolived
D_X_CECUM_DISS_DT_DMPH       =
VCECUM*Kd_IR*C_CECUM_SOLID_DMPHIR*(SOLIF_CECUM-C_CECUM_DISS_DMPH)+D_released_D
MPHER_CECUM-NI_CECUM*DIFF_CECUM*(-C_CECUM_MEM_DMPH/1000+C_CECUM_DISS_DMPH)+
(X_ILL3_DISS_DMPH/TILL3)- (X_CECUM_DISS_DMPH/TCECUM)!
X_CECUM_DISS_DMPH            = INTEG(D_X_CECUM_DISS_DT_DMPH, 0.0)
C_CECUM_DISS_DMPH            = X_CECUM_DISS_DMPH/VCECUM

D_dissolve_solid_dir_cecum   =
VCECUM*Kd_IR*C_CECUM_SOLID_DMPHIR*(SOLIF_CECUM-C_CECUM_DISS_DMPH)
x_dissolve_solid_dir_cecum   = integ(D_dissolve_solid_dir_cecum, 0.0)

!Enterocytes
D_MEM_CECUM_DT_DMPH          =
QCECUM*CAD-QCECUM*(MEM_CECUM_DMPH/(VMEMCECUM*Kpcolon))*1 -
METABOLISM_CECUM_DMPH
+NI_CECUM*DIFF_CECUM*(-C_CECUM_MEM_DMPH/1000+C_CECUM_DISS_DMPH) ! - EFFLUX_DUO
+ INFLUX_DUO  !ug/hr
MEM_CECUM_DMPH               = INTEG(D_MEM_CECUM_DT_DMPH,0.0)  !!ug
C_CECUM_MEM_DMPH             = MEM_CECUM_DMPH/VMEMCECUM         !!ug/L
R_MEM_CECUM_DMPH             = QCECUM*(MEM_CECUM_DMPH/(VMEMCECUM*Kpcolon))*1
!!ug/hr
A_MEM_CECUM_DMPH             = integ(R_MEM_CECUM_DMPH, 0.0)    !!ug
METABOLISM_CECUM_DMPH        = K5D_CECUM*MEM_CECUM_DMPH            !!ug/hr
AMETABOLISM_CECUM_DMPH       = INTEG(METABOLISM_CECUM_DMPH, 0.0)  !!ug

D_ABS_CECUM_DMPH             =
NI_CECUM*DIFF_CECUM*(-C_CECUM_MEM_DMPH/1000+C_CECUM_DISS_DMPH)
X_ABS_CECUM_DMPH             = INTEG(D_ABS_CECUM_DMPH, 0.0)

D_FG_CECUM_DMPH              =
-QCECUM*CAD+QCECUM*(MEM_CECUM_DMPH/(VMEMCECUM*Kpcolon))*1
X_FG_CECUM_DMPH              = INTEG(D_FG_CECUM_DMPH, 0.0)


! ACSENDING COLON
!Lumen SOLID
X_released_DMPHER_ASCENDING  = integ(D_released_DMPHER_ASCENDING ,0.0)  !! ug
D_released_DMPHER_ASCENDING  = release_ascending
D_ASCENDING_FORM_DMPHER      = -D_released_DMPHER_ASCENDING
+X_CECUM_FORM_DMPHER/TCECUM -X_ASCENDING_FORM_DMPHER/TASCENDING
X_ASCENDING_FORM_DMPHER      = integ(D_ASCENDING_FORM_DMPHER, 0.0)  !! ug
C_ASCENDING_FORM_DMPHER      = X_ASCENDING_FORM_DMPHER/VASCENDING       !!
ug/mL

D_X_ASCENDING_SOLID_DT_DMPHIR    =
-VASCENDING*Kd_IR*C_ASCENDING_SOLID_DMPHIR*(SOLIF_ASCENDING-C_ASCENDING_DISS_D
MPH) +X_CECUM_SOLID_DMPHIR/TCECUM-(X_ASCENDING_SOLID_DMPHIR/TASCENDING)
X_ASCENDING_SOLID_DMPHIR         = BOUND(0.0,
ADOSEDIR,LIMINT(D_X_ASCENDING_SOLID_DT_DMPHIR,0.0, 0.0, ADOSEDIR))
C_ASCENDING_SOLID_DMPHIR         = X_ASCENDING_SOLID_DMPHIR/VASCENDING

!Lumen_dissolived
D_X_ASCENDING_DISS_DT_DMPH           =
VASCENDING*Kd_IR*C_ASCENDING_SOLID_DMPHIR*(SOLIF_ASCENDING-C_ASCENDING_DISS_DM
PH)+D_released_DMPHER_ASCENDING-(NI_ASCENDING*DIFF_ASCENDING*(-C_ASCENDING_MEM
_DMPH/1000+C_ASCENDING_DISS_DMPH)) +X_CECUM_DISS_DMPH/TCECUM
-X_ASCENDING_DISS_DMPH/TASCENDING
```

```
X_ASCENDING_DISS_DMPH            = INTEG(D_X_ASCENDING_DISS_DT_DMPH, 0.0)
C_ASCENDING_DISS_DMPH            = X_ASCENDING_DISS_DMPH/VASCENDING

D_dissolve_solid_dir_ascending   = 
VASCENDING*Kd_IR*C_ASCENDING_SOLID_DMPHIR*(SOLIF_ASCENDING-C_ASCENDING_DISS_DM
PH)
x_dissolve_solid_dir_ascending   = integ(D_dissolve_solid_dir_ascending, 0.0)

!Enterocytes
D_MEM_ASCENDING_DT_DMPH          = 
QASCENDING*CAD-QASCENDING*(MEM_ASCENDING_DMPH/(VMEMASCENDING*Kpcolon))*1 - 
METABOLISM_ASCENDING_DMPH
+NI_ASCENDING*DIFF_ASCENDING*(-C_ASCENDING_MEM_DMPH/1000+C_ASCENDING_DISS_DMPH
) ! - EFFLUX_DUO + INFLUX_DUO  !ug/hr
MEM_ASCENDING_DMPH               = INTEG(D_MEM_ASCENDING_DT_DMPH,0.0)  !!ug
C_ASCENDING_MEM_DMPH             = MEM_ASCENDING_DMPH/VMEMASCENDING
!!ug/L
R_MEM_ASCENDING_DMPH             = 
QASCENDING*(MEM_ASCENDING_DMPH/(VMEMASCENDING*Kpcolon))*1  !!ug/hr
A_MEM_ASCENDING_DMPH             = integ(R_MEM_ASCENDING_DMPH, 0.0)    !!ug
METABOLISM_ASCENDING_DMPH        = K5D_ASCENDING*MEM_ASCENDING_DMPH
!!ug/hr
AMETABOLISM_ASCENDING_DMPH       = INTEG(METABOLISM_ASCENDING_DMPH, 0.0)  !!ug

D_ABS_ASCENDING_DMPH             = 
NI_ASCENDING*DIFF_ASCENDING*(-C_ASCENDING_MEM_DMPH/1000+C_ASCENDING_DISS_DMPH)

X_ABS_ASCENDING_DMPH             = INTEG(D_ABS_ASCENDING_DMPH, 0.0)

D_FG_ASCENDING_DMPH              = 
-QASCENDING*CAD+QASCENDING*(MEM_ASCENDING_DMPH/(VMEMASCENDING*Kpcolon))*1
X_FG_ASCENDING_DMPH              = INTEG(D_FG_ASCENDING_DMPH, 0.0)

!Terminal-unabsorbed DMPH_IR
D_Terminal_SOLID_DMPH            = 
X_ASCENDING_SOLID_DMPHIR/TASCENDING+X_ASCENDING_FORM_DMPHER/TASCENDING
A_Terminal_SOLID_DMPH            = BOUND(0.0, ADOSED, 
LIMINT(D_Terminal_SOLID_DMPH,0.0, 0.0, ADOSED))

D_Terminal_DISS_DMPH             = X_ASCENDING_DISS_DMPH/TASCENDING
A_Terminal_DISS_DMPH             = INTEG(D_Terminal_DISS_DMPH, 0.0)

!Add the absorption rate constants up

RBS_DMPH            = 
QDUO*(MEM_DUO_DMPH/(VMEMDUO*KpGut))*1+QJEJ1*(MEM_JEJ1_DMPH/(VMEMJEJ1*KpGut))*1
+QJEJ2*(MEM_JEJ2_DMPH/(VMEMJEJ2*KpGut))*1+QILL1*(MEM_ILL1_DMPH/(VMEMILL1*KpGut
))*1+QILL2*(MEM_ILL2_DMPH/(VMEMILL2*KpGut))*1+QILL3*(MEM_ILL3_DMPH/(VMEMILL3*K
pGut))*1+
QCECUM*(MEM_CECUM_DMPH/(VMEMCECUM*Kpcolon))*1+QASCENDING*(MEM_ASCENDING_DMPH
/(VMEMASCENDING*Kpcolon))*1
ABS_DMPH            = integ(RBS_DMPH, 0.0)

AMETABOLISM_SI_DMPH = 
AMETABOLISM_DUO_DMPH+AMETABOLISM_JEJ1_DMPH+AMETABOLISM_JEJ2_DMPH+AMETABOLISM_I
LL1_DMPH+AMETABOLISM_ILL2_DMPH+AMETABOLISM_ILL3_DMPH+AMETABOLISM_CECUM_DMPH+AM
ETABOLISM_ASCENDING_DMPH
METABOLISM_SI_DMPH  = 
METABOLISM_DUO_DMPH+METABOLISM_JEJ1_DMPH+METABOLISM_JEJ2_DMPH+METABOLISM_ILL1_
DMPH+METABOLISM_ILL2_DMPH+METABOLISM_ILL3_DMPH+METABOLISM_CECUM_DMPH
+METABOLISM_ASCENDING_DMPH

!!!%%%%%%%%%%%%%%%%%%%%%%%%%%%%%%%%%%%%%%%%%%%FOR
L-MPH%%%%%%%%%%%%%%%%%%%%%%%%%%%%%%%%%%%%%%%%%%%%%

X_released_LMPHER_stomach       = integ(D_released_LMPHER_stomach,0.0)
```

```
D_released_LMPHER_stomach        = RELEASE_stomach
D_X_STOMACH_FORM_DT_LMPHER       = -D_released_LMPHER_stomach
-X_STOMACH_FORM_DR_LMPHER/Tstomach
X_STOMACH_FORM_DR_LMPHER         = INTEG(D_X_STOMACH_FORM_DT_LMPHER, Adoseler)

C_STOMACH_FORM_LMPHER            = X_STOMACH_FORM_DR_LMPHER/Vstomach

D_X_STOMACH_SOLID_DT_LMPHIR      =
-VSTOMACH*Kd_IR*C_STOMACH_SOLID_LMPHIR*(SOLIF_STOMACH-C_STOMACH_DISS_LMPH)-X_S
TOMACH_SOLID_LMPHIR/Tstomach ! ug/hr (ok)
X_STOMACH_SOLID_LMPHIR           = BOUND(0.0,AdoselIR,
LIMINT(D_X_STOMACH_SOLID_DT_LMPHIR,AdoselIR, 0.0, AdoselIR))  ! ug  (ok)
C_STOMACH_SOLID_LMPHIR           = X_STOMACH_SOLID_LMPHIR/VSTOMACH
                        ! ug/ml  (ok)

Adosel                           = AdoselIR+AdoselER

!!Lumen dissolved
D_X_STOMACH_DISS_DT_LMPH         =
VSTOMACH*Kd_IR*C_STOMACH_SOLID_LMPHIR*(SOLIF_STOMACH-C_STOMACH_DISS_LMPH)-X_ST
OMACH_DISS_LMPH/Tstomach+D_released_LMPHER_stomach
X_STOMACH_DISS_LMPH              = integ(D_X_STOMACH_DISS_DT_LMPH,0.0)
! ug  (ok)
C_STOMACH_DISS_LMPH              = X_STOMACH_DISS_LMPH/VSTOMACH               !
ug/ml  (ok)


D_dissolve_solid_LIR_stomach     =
VSTOMACH*Kd_IR*C_STOMACH_SOLID_LMPHIR*(SOLIF_STOMACH-C_STOMACH_DISS_LMPH)
x_dissolve_solid_LIR_stomach     = integ(D_dissolve_solid_LIR_stomach, 0.0)

!DUODENUM
!Lumen_solid
X_released_LMPHER_DUO            = integ(D_released_LMPHER_DUO,0.0)  !! ug
D_released_LMPHER_DUO            = release_duo
D_duo_FORM_LMPHER                = -D_released_LMPHER_DUO +
X_STOMACH_FORM_DR_LMPHER/Tstomach-(X_duo_FORM_LMPHER/TDUO)
X_duo_FORM_LMPHER                = integ(D_duo_FORM_LMPHER, 0.0)  !! ug
C_DUO_FORM_LMPHER                = X_duo_FORM_LMPHER/VDUO       !! ug/mL

D_X_DUO_SOLID_DT_LMPHIR          =
-VDUO*Kd_IR*C_DUO_SOLID_LMPHIR*(SOLIF_DUO-C_DUO_DISS_LMPH)+X_STOMACH_SOLID_LMP
HIR/Tstomach -(X_DUO_SOLID_LMPHIR/TDUO)
X_DUO_SOLID_LMPHIR               = BOUND(0.0,
AdoselIR,LIMINT(D_X_DUO_SOLID_DT_LMPHIR,0.0, 0.0, AdoselIR))    !ug
C_DUO_SOLID_LMPHIR               = X_DUO_SOLID_LMPHIR/VDUO

!Lumen_dissolived
D_X_DUO_DISS_DT_LMPH               =
VDUO*Kd_IR*C_DUO_SOLID_LMPHIR*(SOLIF_DUO-C_DUO_DISS_LMPH)+D_released_LMPHER_DU
O-NI_DUO*DIFF_DUO*(-C_DUO_MEM_LMPH/1000+C_DUO_DISS_LMPH)
+X_STOMACH_DISS_LMPH/Tstomach-X_DUO_DISS_LMPH/TDUO
X_DUO_DISS_LMPH                  = INTEG(D_X_DUO_DISS_DT_LMPH,0.0)
C_DUO_DISS_LMPH                  = X_DUO_DISS_LMPH/VDUO

D_dissolve_solid_LIR_duo         =
VDUO*Kd_IR*C_DUO_SOLID_LMPHIR*(SOLIF_DUO-C_DUO_DISS_LMPH)
X_dissolve_solid_LIR_duo         = INTEG(D_dissolve_solid_LIR_duo, 0.0)

!Enterocytes
D_MEM_DUO_DT_LMPH                =
QDUO*CAL-QDUO*(MEM_DUO_LMPH/(VMEMDUO*KpGut))*1 - METABOLISM_DUO_LMPH
+NI_DUO*DIFF_DUO*(-C_DUO_MEM_LMPH/1000+C_DUO_DISS_LMPH) ! - EFFLUX_DUO +
INFLUX_DUO  !ug/hr
MEM_DUO_LMPH                     = INTEG(D_MEM_DUO_DT_LMPH,0.0)  !!ug
C_DUO_MEM_LMPH                   = MEM_DUO_LMPH/VMEMDUO          !!ug/L
```

```
R_MEM_DUO_LMPH                  = QDUO*(MEM_DUO_LMPH/(VMEMDUO*KpGut))*1
!!ug/hr
A_MEM_DUO_LMPH                  = integ(R_MEM_DUO_LMPH, 0.0)    !!ug
METABOLISM_DUO_LMPH             = K5l*MEM_DUO_LMPH             !!ug/hr
AMETABOLISM_DUO_LMPH            = INTEG(METABOLISM_DUO_LMPH, 0.0)  !!ug

D_ABS_DUO_LMPH                  =
NI_DUO*DIFF_DUO*(-C_DUO_MEM_LMPH/1000+C_DUO_DISS_LMPH)
X_ABS_DUO_LMPH                  = INTEG(D_ABS_DUO_LMPH, 0.0)

D_FG_DUO_LMPH                   =
-QDUO*CAL+QDUO*(MEM_DUO_LMPH/(VMEMDUO*KpGut))*1
X_FG_DUO_LMPH                   = INTEG(D_FG_DUO_LMPH, 0.0)

!JEJUNUM1
!Lumen solid
X_released_LMPHER_JEJ1         = integ(D_released_LMPHER_JEJ1,0.0)  !! ug
D_released_LMPHER_JEJ1         = release_jej1
D_JEJ1_FORM_LMPHER             = -D_released_LMPHER_JEJ1
-X_JEJ1_FORM_LMPHER/TJEJ1  +X_DUO_FORM_LMPHER/TDUO
X_JEJ1_FORM_LMPHER             = integ(D_JEJ1_FORM_LMPHER, 0.0)  !! ug
C_JEJ1_FORM_LMPHER             = X_JEJ1_FORM_LMPHER/VJEJ1       !! ug/mL

D_X_JEJ1_SOLID_DT_LMPHIR       =
-Vjej1*Kd_IR*C_JEJ1_SOLID_LMPHIR*(SOLIF_JEJ1-C_JEJ1_DISS_LMPH)-(X_JEJ1_SOLID_L
MPHIR/TJEJ1)+ X_DUO_SOLID_LMPHIR/TDUO
X_JEJ1_SOLID_LMPHIR            = BOUND(0.0,
AdoselIR,LIMINT(D_X_JEJ1_SOLID_DT_LMPHIR,0.0, 0.0, AdoselIR))    !ug
C_JEJ1_SOLID_LMPHIR            = X_JEJ1_SOLID_LMPHIR/VJEJ1

!Lumen_dissolived
D_X_JEJ1_DISS_DT_LMPH           =
VJEJ1*Kd_IR*C_JEJ1_SOLID_LMPHIR*(SOLIF_JEJ1-C_JEJ1_DISS_LMPH)+D_released_LMPHE
R_JEJ1-NI_JEJ1*DIFF_JEJ1*(-C_JEJ1_MEM_LMPH/1000+C_JEJ1_DISS_LMPH)
-X_JEJ1_DISS_LMPH/TJEJ1       +X_DUO_DISS_LMPH/TDUO
X_JEJ1_DISS_LMPH               = INTEG(D_X_JEJ1_DISS_DT_LMPH,0.0)
C_JEJ1_DISS_LMPH               = X_JEJ1_DISS_LMPH/VJEJ1

D_dissolve_solid_LIR_JEJ1      =
VJEJ1*Kd_IR*C_JEJ1_SOLID_LMPHIR*(SOLIF_JEJ1-C_JEJ1_DISS_LMPH)
A_dissolve_solid_LIR_JEJ1      = INTEG(D_dissolve_solid_LIR_JEJ1, 0.0)

!Enterocytes
D_MEM_JEJ1_DT_LMPH           =
QJEJ1*CAL-QJEJ1*(MEM_JEJ1_LMPH/(VMEMJEJ1*KpGut))*1 - METABOLISM_JEJ1_LMPH
+NI_JEJ1*DIFF_JEJ1*(-C_JEJ1_MEM_LMPH/1000+C_JEJ1_DISS_LMPH) ! - EFFLUX_DUO +
INFLUX_DUO  !ug/hr
MEM_JEJ1_LMPH                = INTEG(D_MEM_JEJ1_DT_LMPH,0.0)  !!ug
C_JEJ1_MEM_LMPH              = MEM_JEJ1_LMPH/VMEMJEJ1          !!ug/L
R_MEM_JEJ1_LMPH              = QJEJ1*(MEM_JEJ1_LMPH/(VMEMJEJ1*KpGut))*1
!!ug/hr
A_MEM_JEJ1_LMPH              = integ(R_MEM_JEJ1_LMPH, 0.0)    !!ug
METABOLISM_JEJ1_LMPH         = K5l*MEM_JEJ1_LMPH             !!ug/hr
AMETABOLISM_JEJ1_LMPH        = INTEG(METABOLISM_JEJ1_LMPH, 0.0)  !!ug

D_ABS_JEJ1_LMPH              =
NI_JEJ1*DIFF_JEJ1*(-C_JEJ1_MEM_LMPH/1000+C_JEJ1_DISS_LMPH)
X_ABS_JEJ1_LMPH              = INTEG(D_ABS_JEJ1_LMPH, 0.0)

D_FG_JEJ1_LMPH               =
-QJEJ1*CAL+QJEJ1*(MEM_JEJ1_LMPH/(VMEMJEJ1*KpGut))*1
X_FG_JEJ1_LMPH               = INTEG(D_FG_JEJ1_LMPH, 0.0)

!JEJUNUM2
!Lumen solid
X_released_LMPHER_JEJ2      = integ(D_released_LMPHER_JEJ2,0.0)  !! ug
```

```
D_released_LMPHER_JEJ2      = release_jej2
D_JEJ2_FORM_LMPHER          = -D_released_LMPHER_JEJ2
+X_JEJ1_FORM_LMPHER/TJEJ1 -X_JEJ2_FORM_LMPHER/TJEJ2
X_JEJ2_FORM_LMPHER          = integ(D_JEJ2_FORM_LMPHER, 0.0)  !! ug
C_JEJ2_FORM_LMPHER          = X_JEJ2_FORM_LMPHER/VJEJ2       !! ug/mL

D_X_JEJ2_SOLID_DT_LMPHIR    =
-VJEJ2*Kd_IR*C_JEJ2_SOLID_LMPHIR*(SOLIF_JEJ2-C_JEJ2_DISS_LMPH) +
X_JEJ1_SOLID_LMPHIR/TJEJ1  -(X_JEJ2_SOLID_LMPHIR/TJEJ2)
X_JEJ2_SOLID_LMPHIR         = BOUND(0.0,
AdoselIR,LIMINT(D_X_JEJ2_SOLID_DT_LMPHIR,0.0, 0.0, AdoselIR))    !ug
C_JEJ2_SOLID_LMPHIR         = X_JEJ2_SOLID_LMPHIR/VJEJ2

!Lumen_dissolived
D_X_JEJ2_DISS_DT_LMPH        =
VJEJ2*Kd_IR*C_JEJ2_SOLID_LMPHIR*(SOLIF_JEJ2-C_JEJ2_DISS_LMPH)+D_released_LMPHE
R_JEJ2-NI_JEJ2*DIFF_JEJ2*(-C_JEJ2_MEM_LMPH/1000+C_JEJ2_DISS_LMPH)
+X_JEJ1_DISS_LMPH/TJEJ1 -X_JEJ2_DISS_LMPH/TJEJ2
X_JEJ2_DISS_LMPH            = INTEG(D_X_JEJ2_DISS_DT_LMPH,0.0)
C_JEJ2_DISS_LMPH            = X_JEJ2_DISS_LMPH/VJEJ2

D_dissolve_solid_LIR_JEJ2   =
VJEJ2*Kd_IR*C_JEJ2_SOLID_LMPHIR*(SOLIF_JEJ2-C_JEJ2_DISS_LMPH)
X_dissolve_solid_LIR_JEJ2   = INTEG(D_dissolve_solid_LIR_JEJ2, 0.0)

!Enterocytes
D_MEM_JEJ2_DT_LMPH           =
QJEJ2*CAL-QJEJ2*(MEM_JEJ2_LMPH/(VMEMJEJ2*KpGut))*1 - METABOLISM_JEJ2_LMPH
+NI_JEJ2*DIFF_JEJ2*(-C_JEJ2_MEM_LMPH/1000+C_JEJ2_DISS_LMPH) ! - EFFLUX_JEJ1 +
INFLUX_JEJ1  !ug/hr
MEM_JEJ2_LMPH                = INTEG(D_MEM_JEJ2_DT_LMPH,0.0)  !!ug
C_JEJ2_MEM_LMPH              = MEM_JEJ2_LMPH/VMEMJEJ2          !!ug/L
R_MEM_JEJ2_LMPH              = QJEJ2*(MEM_JEJ2_LMPH/(VMEMJEJ2*KpGut))*1
!!ug/hr
A_MEM_JEJ2_LMPH              = integ(R_MEM_JEJ2_LMPH, 0.0)    !!ug
METABOLISM_JEJ2_LMPH         = K5l*MEM_JEJ2_LMPH            !!ug/hr
AMETABOLISM_JEJ2_LMPH        = INTEG(METABOLISM_JEJ2_LMPH, 0.0)  !!ug

D_ABS_JEJ2_LMPH              =
NI_JEJ2*DIFF_JEJ2*(-C_JEJ2_MEM_LMPH/1000+C_JEJ2_DISS_LMPH)
X_ABS_JEJ2_LMPH              = INTEG(D_ABS_JEJ2_LMPH, 0.0)

D_FG_JEJ2_LMPH               =
-QJEJ2*CAL+QJEJ2*(MEM_JEJ2_LMPH/(VMEMJEJ2*KpGut))*1
X_FG_JEJ2_LMPH               = INTEG(D_FG_JEJ2_LMPH, 0.0)

!ILLEUM1
!Lumen solid
X_released_LMPHER_ILL1      = integ(D_released_LMPHER_ILL1,0.0)  !! ug
D_released_LMPHER_ILL1      = release_ill1
D_ILL1_FORM_LMPHER          = -D_released_LMPHER_ILL1
+X_JEJ2_FORM_LMPHER/TJEJ2 -X_ILL1_FORM_LMPHER/TILL1
X_ILL1_FORM_LMPHER          = integ(D_ILL1_FORM_LMPHER, 0.0)  !! ug
C_ILL1_FORM_LMPHER          = X_ILL1_FORM_LMPHER/VILL1       !! ug/mL

D_X_ILL1_SOLID_DT_LMPHIR    =
-VILL1*Kd_IR*C_ILL1_SOLID_LMPHIR*(SOLIF_ILL1-C_ILL1_DISS_LMPH)
+X_JEJ2_SOLID_LMPHIR/TJEJ2 -(X_ILL1_SOLID_LMPHIR/TILL1)
X_ILL1_SOLID_LMPHIR         = BOUND(0.0,
AdoselIR,LIMINT(D_X_ILL1_SOLID_DT_LMPHIR,0.0, 0.0, AdoselIR))    !ug
C_ILL1_SOLID_LMPHIR         = X_ILL1_SOLID_LMPHIR/VILL1

!Lumen_dissolived
D_X_ILL1_DISS_DT_LMPH        =
VILL1*Kd_IR*C_ILL1_SOLID_LMPHIR*(SOLIF_ILL1-C_ILL1_DISS_LMPH)+D_released_LMPHE
R_ILL1-NI_ILL1*DIFF_ILL1*(-C_ILL1_MEM_LMPH/1000+C_ILL1_DISS_LMPH)+X_JEJ2_DISS_
```

```
LMPH/TJEJ2 -X_ill1_DISS_LMPH/TILL1
X_ILL1_DISS_LMPH             = INTEG(D_X_ILL1_DISS_DT_LMPH,0.0)
C_ILL1_DISS_LMPH             = X_ILL1_DISS_LMPH/VILL1

D_dissolve_solid_LIR_ILL1   =
VILL1*Kd_IR*C_ILL1_SOLID_LMPHIR*(SOLIF_ILL1-C_ILL1_DISS_LMPH)
X_dissolve_solid_LIR_ILL1   = INTEG(D_dissolve_solid_LIR_ILL1, 0.0)

!Enterocytes
D_MEM_ILL1_DT_LMPH            =
QILL1*CAL-QILL1*(MEM_ILL1_LMPH/(VMEMILL1*KpGut))*1 - METABOLISM_ILL1_LMPH
+NI_ILL1*DIFF_ILL1*(-C_ILL1_MEM_LMPH/1000+C_ILL1_DISS_LMPH) ! - EFFLUX_DUO +
INFLUX_DUO  !ug/hr
MEM_ILL1_LMPH                 = INTEG(D_MEM_ILL1_DT_LMPH,0.0)  !!ug
C_ILL1_MEM_LMPH               = MEM_ILL1_LMPH/VMEMILL1          !!ug/L
R_MEM_ILL1_LMPH               = QILL1*(MEM_ILL1_LMPH/(VMEMILL1*KpGut))*1
!!ug/hr
A_MEM_ILL1_LMPH               = integ(R_MEM_ILL1_LMPH, 0.0)    !!ug
METABOLISM_ILL1_LMPH          = K5l*MEM_ILL1_LMPH            !!ug/hr
AMETABOLISM_ILL1_LMPH         = INTEG(METABOLISM_ILL1_LMPH, 0.0)  !!ug

D_ABS_ILL1_LMPH               =
NI_ILL1*DIFF_ILL1*(-C_ILL1_MEM_LMPH/1000+C_ILL1_DISS_LMPH)
X_ABS_ILL1_LMPH               = INTEG(D_ABS_ILL1_LMPH, 0.0)

D_FG_ILL1_LMPH                =
-QILL1*CAL+QILL1*(MEM_ILL1_LMPH/(VMEMILL1*KpGut))*1
X_FG_ILL1_LMPH                = INTEG(D_FG_ILL1_LMPH, 0.0)

!ILLEUM2
!Lumen
X_released_LMPHER_ILL2      = integ(D_released_LMPHER_ILL2,0.0)  !! ug
D_released_LMPHER_ILL2      = release_ill2
D_ILL2_FORM_LMPHER          = -D_released_LMPHER_ILL2 +
X_ILL1_FORM_LMPHER/TILL1-X_ILL2_FORM_LMPHER/TILL2
X_ILL2_FORM_LMPHER          = integ(D_ILL2_FORM_LMPHER, 0.0)  !! ug
C_ILL2_FORM_LMPHER          = X_ILL2_FORM_LMPHER/VILL2       !! ug/mL

D_X_ILL2_SOLID_DT_LMPHIR    =
-VILL2*Kd_IR*C_ILL2_SOLID_LMPHIR*(SOLIF_ILL2-C_ILL2_DISS_LMPH)+
X_ILL1_SOLID_LMPHIR/TILL1-(X_ILL2_SOLID_LMPHIR/TILL2)
X_ILL2_SOLID_LMPHIR         = BOUND(0.0,
AdoselIR,LIMINT(D_X_ILL2_SOLID_DT_LMPHIR,0.0, 0.0, AdoselIR))    !ug
C_ILL2_SOLID_LMPHIR         = X_ILL2_SOLID_LMPHIR/VILL2

!Lumen_dissolived
D_X_ILL2_DISS_DT_LMPH       =
VILL2*Kd_IR*C_ILL2_SOLID_LMPHIR*(SOLIF_ILL2-C_ILL2_DISS_LMPH)+D_released_LMPHE
R_ILL2-NI_ILL2*DIFF_ILL2*(-C_ILL2_MEM_LMPH/1000+C_ILL2_DISS_LMPH)
+X_ILL1_DISS_LMPH/TILL1-X_ILL2_DISS_LMPH/TILL2
X_ILL2_DISS_LMPH            = INTEG(D_X_ILL2_DISS_DT_LMPH,0.0)
C_ILL2_DISS_LMPH            = X_ILL2_DISS_LMPH/VILL2

D_dissolve_solid_LIR_ILL2   =
VILL2*Kd_IR*C_ILL2_SOLID_LMPHIR*(SOLIF_ILL2-C_ILL2_DISS_LMPH)
X_dissolve_solid_LIR_ILL2   = INTEG(D_dissolve_solid_LIR_ILL2, 0.0)

!Enterocytes
D_MEM_ILL2_DT_LMPH            =
QILL2*CAL-QILL2*(MEM_ILL2_LMPH/(VMEMILL2*KpGut))*1 - METABOLISM_ILL2_LMPH
+NI_ILL2*DIFF_ILL2*(-C_ILL2_MEM_LMPH/1000+C_ILL2_DISS_LMPH) ! - EFFLUX_DUO +
INFLUX_DUO  !ug/hr
MEM_ILL2_LMPH                 = INTEG(D_MEM_ILL2_DT_LMPH,0.0)  !!ug
C_ILL2_MEM_LMPH               = MEM_ILL2_LMPH/VMEMILL2          !!ug/L
R_MEM_ILL2_LMPH               = QILL2*(MEM_ILL2_LMPH/(VMEMILL2*KpGut))*1
!!ug/hr
```

```
A_MEM_ILL2_LMPH              = integ(R_MEM_ILL2_LMPH, 0.0)    !!ug
METABOLISM_ILL2_LMPH         = K5L*MEM_ILL2_LMPH            !!ug/hr
AMETABOLISM_ILL2_LMPH        = INTEG(METABOLISM_ILL2_LMPH, 0.0)  !!ug

D_ABS_ILL2_LMPH              =
NI_ILL2*DIFF_ILL2*(-C_ILL2_MEM_LMPH/1000+C_ILL2_DISS_LMPH)
X_ABS_ILL2_LMPH              = INTEG(D_ABS_ILL2_LMPH, 0.0)

D_FG_ILL2_LMPH               =
-QILL2*CAL+QILL2*(MEM_ILL2_LMPH/(VMEMILL2*KpGut))*1
X_FG_ILL2_LMPH               = INTEG(D_FG_ILL2_LMPH, 0.0)

!ILLEUM3
X_released_LMPHER_ILL3      = integ(D_released_LMPHER_ILL3,0.0)  !! ug
D_released_LMPHER_ILL3      = release_ill3
D_ILL3_FORM_LMPHER          = -D_released_LMPHER_ILL3+X_ILL2_FORM_LMPHER/TILL2
-X_ILL3_FORM_LMPHER/TILL3
X_ILL3_FORM_LMPHER          = integ(D_ILL3_FORM_LMPHER, 0.0)  !! ug
C_ILL3_FORM_LMPHER          = X_ILL3_FORM_LMPHER/VILL3       !! ug/mL

D_X_ILL3_SOLID_DT_LMPHIR    =
-VILL3*Kd_IR*C_ILL3_SOLID_LMPHIR*(SOLIF_ILL3-C_ILL3_DISS_LMPH)+X_ILL2_SOLID_LM
PHIR/TILL2 -(X_ILL3_SOLID_LMPHIR/TILL3)
X_ILL3_SOLID_LMPHIR         = BOUND(0.0,
AdoselIR,LIMINT(D_X_ILL3_SOLID_DT_LMPHIR,0.0, 0.0, AdoselIR))    !ug
C_ILL3_SOLID_LMPHIR         = X_ILL3_SOLID_LMPHIR/VILL3

!Lumen_dissolived
D_X_ILL3_DISS_DT_LMPH       =
VILL3*Kd_IR*C_ILL3_SOLID_LMPHIR*(SOLIF_ILL3-C_ILL3_DISS_LMPH)+D_released_LMPHE
R_ILL3-NI_ILL3*DIFF_ILL3*(-C_ILL3_MEM_LMPH/1000+C_ILL3_DISS_LMPH)+X_ILL2_DISS_
LMPH/TILL2 -X_ILL3_DISS_LMPH/TILL3
X_ILL3_DISS_LMPH            = INTEG(D_X_ILL3_DISS_DT_LMPH,0.0)
C_ILL3_DISS_LMPH            = X_ILL3_DISS_LMPH/VILL3

D_dissolve_solid_LIR_ILL3   =
VILL3*Kd_IR*C_ILL3_SOLID_LMPHIR*(SOLIF_ILL3-C_ILL3_DISS_LMPH)
X_dissolve_solid_LIR_ILL3   = INTEG(D_dissolve_solid_LIR_ILL3, 0.0)

!Enterocytes
D_MEM_ILL3_DT_LMPH           =
QILL3*CAL-QILL3*(MEM_ILL3_LMPH/(VMEMILL3*KpGut))*1 - METABOLISM_ILL3_LMPH
+NI_ILL3*DIFF_ILL3*(-C_ILL3_MEM_LMPH/1000+C_ILL3_DISS_LMPH) ! - EFFLUX_DUO +
INFLUX_DUO  !ug/hr
MEM_ILL3_LMPH                = INTEG(D_MEM_ILL3_DT_LMPH,0.0)  !!ug
C_ILL3_MEM_LMPH              = MEM_ILL3_LMPH/VMEMILL3          !!ug/L
R_MEM_ILL3_LMPH              = QILL3*(MEM_ILL3_LMPH/(VMEMILL3*KpGut))*1
!!ug/hr
A_MEM_ILL3_LMPH              = integ(R_MEM_ILL3_LMPH, 0.0)    !!ug
METABOLISM_ILL3_LMPH         = K5L*MEM_ILL3_LMPH            !!ug/hr
AMETABOLISM_ILL3_LMPH        = INTEG(METABOLISM_ILL3_LMPH, 0.0)  !!ug

D_ABS_ILL3_LMPH              =
NI_ILL3*DIFF_ILL3*(-C_ILL3_MEM_LMPH/1000+C_ILL3_DISS_LMPH)
X_ABS_ILL3_LMPH              = INTEG(D_ABS_ILL3_LMPH, 0.0)

D_FG_ILL3_LMPH               =
-QILL3*CAL+QILL3*(MEM_ILL3_LMPH/(VMEMILL3*KpGut))*1
X_FG_ILL3_LMPH               = INTEG(D_FG_ILL3_LMPH, 0.0)

!Cecum
!Lumen SOLID
X_released_LMPHER_CECUM      = integ(D_released_LMPHER_CECUM,0.0)  !! ug
D_released_LMPHER_CECUM      = release_cecum
D_CECUM_FORM_LMPHER          = -D_released_LMPHER_CECUM +
X_ILL3_FORM_LMPHER/TILL3-X_CECUM_FORM_LMPHER/TCECUM
```

```
X_CECUM_FORM_LMPHER           = integ(D_CECUM_FORM_LMPHER, 0.0)  !! ug
C_CECUM_FORM_LMPHER           = X_CECUM_FORM_LMPHER/VCECUM       !! ug/mL

D_X_CECUM_SOLID_DT_LMPHIR    =
-VCECUM*Kd_IR*C_CECUM_SOLID_LMPHIR*(SOLIF_CECUM-C_CECUM_DISS_LMPH) +
X_ILL3_SOLID_LMPHIR/TILL3-(X_CECUM_SOLID_LMPHIR/TCECUM)
X_CECUM_SOLID_LMPHIR          = BOUND(0.0,
AdoselIR,LIMINT(D_X_CECUM_SOLID_DT_LMPHIR,0.0, 0.0, AdoselIR))    !ug
C_CECUM_SOLID_LMPHIR          = X_CECUM_SOLID_LMPHIR/VCECUM

!Lumen_dissolived
D_X_CECUM_DISS_DT_LMPH       =
VCECUM*Kd_IR*C_CECUM_SOLID_LMPHIR*(SOLIF_CECUM-C_CECUM_DISS_LMPH)+D_released_L
MPHER_CECUM-NI_CECUM*DIFF_CECUM*(-C_CECUM_MEM_LMPH/1000+C_CECUM_DISS_LMPH)+
(X_ILL3_DISS_LMPH/TILL3)- (X_CECUM_DISS_LMPH/TCECUM)
X_CECUM_DISS_LMPH             = INTEG(D_X_CECUM_DISS_DT_LMPH,0.0)
C_CECUM_DISS_LMPH             = X_CECUM_DISS_LMPH/VCECUM

D_dissolve_solid_LIR_CECUM   =
VCECUM*Kd_IR*C_CECUM_SOLID_LMPHIR*(SOLIF_CECUM-C_CECUM_DISS_LMPH)
X_dissolve_solid_LIR_CECUM   = INTEG(D_dissolve_solid_LIR_CECUM, 0.0)

!Enterocytes
D_MEM_CECUM_DT_LMPH           =
QCECUM*CAL-QCECUM*(MEM_CECUM_LMPH/(VMEMCECUM*Kpcolon))*1 -
METABOLISM_CECUM_LMPH
+NI_CECUM*DIFF_CECUM*(-C_CECUM_MEM_LMPH/1000+C_CECUM_DISS_LMPH) ! - EFFLUX_DUO
+ INFLUX_DUO  !ug/hr
MEM_CECUM_LMPH                = INTEG(D_MEM_CECUM_DT_LMPH,0.0)  !!ug
C_CECUM_MEM_LMPH              = MEM_CECUM_LMPH/VMEMCECUM         !!ug/L
R_MEM_CECUM_LMPH              = QCECUM*(MEM_CECUM_LMPH/(VMEMCECUM*Kpcolon))*1
!!ug/hr
A_MEM_CECUM_LMPH              = integ(R_MEM_CECUM_LMPH, 0.0)    !!ug
METABOLISM_CECUM_LMPH         = K5L_CECUM*MEM_CECUM_LMPH            !!ug/hr
AMETABOLISM_CECUM_LMPH        = INTEG(METABOLISM_CECUM_LMPH, 0.0)  !!ug

D_ABS_CECUM_LMPH      =
NI_CECUM*DIFF_CECUM*(-C_CECUM_MEM_LMPH/1000+C_CECUM_DISS_LMPH)
X_ABS_CECUM_LMPH      = INTEG(D_ABS_CECUM_LMPH, 0.0)

D_FG_CECUM_LMPH       =
-QCECUM*CAL+QCECUM*(MEM_CECUM_LMPH/(VMEMCECUM*Kpcolon))*1
X_FG_CECUM_LMPH       = INTEG(D_FG_CECUM_LMPH, 0.0)


! ACSENDING COLON
!Lumen SOLID
X_released_LMPHER_ASCENDING      = integ(D_released_LMPHER_ASCENDING ,0.0)  !!
ug
D_released_LMPHER_ASCENDING      = release_ascending
D_ASCENDING_FORM_LMPHER          =
-D_released_LMPHER_ASCENDING+X_CECUM_FORM_LMPHER/TCECUM
-X_ASCENDING_FORM_LMPHER/TASCENDING
X_ASCENDING_FORM_LMPHER          = integ(D_ASCENDING_FORM_LMPHER, 0.0)  !! ug
C_ASCENDING_FORM_LMPHER          = X_ASCENDING_FORM_LMPHER/VASCENDING       !!
ug/mL

D_X_ASCENDING_SOLID_DT_LMPHIR    =
-VASCENDING*Kd_IR*C_ASCENDING_SOLID_LMPHIR*(SOLIF_ASCENDING-C_ASCENDING_DISS_L
MPH)+X_CECUM_SOLID_LMPHIR/TCECUM -(X_ASCENDING_SOLID_LMPHIR/TASCENDING)
X_ASCENDING_SOLID_LMPHIR         = BOUND(0.0,
AdoselIR,LIMINT(D_X_ASCENDING_SOLID_DT_LMPHIR,0.0, 0.0, AdoselIR))
C_ASCENDING_SOLID_LMPHIR         = X_ASCENDING_SOLID_LMPHIR/VASCENDING

!Lumen_dissolived
D_X_ASCENDING_DISS_DT_LMPH           =
```

```
VASCENDING*Kd_IR*C_ASCENDING_SOLID_LMPHIR*(SOLIF_ASCENDING-C_ASCENDING_DISS_LM
PH)+D_released_LMPHER_ASCENDING-(NI_ASCENDING*DIFF_ASCENDING*(-C_ASCENDING_MEM
_LMPH/1000+C_ASCENDING_DISS_LMPH))+X_CECUM_DISS_LMPH/TCECUM
-X_ASCENDING_DISS_LMPH/TASCENDING
X_ASCENDING_DISS_LMPH            = integ(D_X_ASCENDING_DISS_DT_LMPH,0.0)
C_ASCENDING_DISS_LMPH            = X_ASCENDING_DISS_LMPH/VASCENDING

D_dissolve_solid_LIR_ASCENDING   =
VASCENDING*Kd_IR*C_ASCENDING_SOLID_LMPHIR*(SOLIF_ASCENDING-C_ASCENDING_DISS_LM
PH)
X_dissolve_solid_LIR_ASCENDING   = INTEG(D_dissolve_solid_LIR_ASCENDING, 0.0)

!Enterocytes
D_MEM_ASCENDING_DT_LMPH           =
QASCENDING*CAL-QASCENDING*(MEM_ASCENDING_LMPH/(VMEMASCENDING*Kpcolon))*1 -
METABOLISM_ASCENDING_LMPH
+NI_ASCENDING*DIFF_ASCENDING*(-C_ASCENDING_MEM_LMPH/1000+C_ASCENDING_DISS_LMPH
) ! - EFFLUX_DUO + INFLUX_DUO  !ug/hr
MEM_ASCENDING_LMPH                = INTEG(D_MEM_ASCENDING_DT_LMPH,0.0)  !!ug
C_ASCENDING_MEM_LMPH              = MEM_ASCENDING_LMPH/VMEMASCENDING
!!ug/L
R_MEM_ASCENDING_LMPH              =
QASCENDING*(MEM_ASCENDING_LMPH/(VMEMASCENDING*Kpcolon))*1  !!ug/hr
A_MEM_ASCENDING_LMPH              = integ(R_MEM_ASCENDING_LMPH, 0.0)    !!ug
METABOLISM_ASCENDING_LMPH         = K5L_ASCENDING*MEM_ASCENDING_LMPH
 !!ug/hr
AMETABOLISM_ASCENDING_LMPH        = INTEG(METABOLISM_ASCENDING_LMPH, 0.0)
!!ug

D_ABS_ASCENDING_LMPH              =
NI_ASCENDING*DIFF_ASCENDING*(-C_ASCENDING_MEM_LMPH/1000+C_ASCENDING_DISS_LMPH)

X_ABS_ASCENDING_LMPH              = INTEG(D_ABS_ASCENDING_LMPH, 0.0)

D_FG_ASCENDING_LMPH               =
-QASCENDING*CAL+QASCENDING*(MEM_ASCENDING_LMPH/(VMEMASCENDING*Kpcolon))*1
X_FG_ASCENDING_LMPH               = INTEG(D_FG_ASCENDING_LMPH, 0.0)


!Terminal-unabsorbed LMPH_IR
D_Terminal_SOLID_LMPH             =
X_ASCENDING_SOLID_LMPHIR/TASCENDING+X_ASCENDING_FORM_LMPHER/TASCENDING
D_Terminal_DISS_LMPH              = X_ASCENDING_DISS_LMPH/TASCENDING
A_Terminal_SOLID_lMPH             = BOUND(0.0, ADOSEL,
LIMINT(D_Terminal_SOLID_LMPH,0.0, 0.0, ADOSEL))
A_Terminal_DISS_LMPH              = INTEG(D_Terminal_DISS_LMPH, 0.0)

D_TERMINAL_IR_LMPH = X_ASCENDING_SOLID_LMPHIR/TASCENDING
X_TERMINAL_IR_LMPH = INTEG(D_TERMINAL_IR_LMPH, 0.0)

D_TERMINAL_ER_LMPH = X_ASCENDING_FORM_LMPHER/TASCENDING
X_TERMINAL_ER_LMPH = INTEG(D_TERMINAL_ER_LMPH, 0.0)

D_TERMINAL_IR_DMPH = X_ASCENDING_SOLID_DMPHIR/TASCENDING
X_TERMINAL_IR_DMPH = INTEG(D_TERMINAL_IR_DMPH, 0.0)

D_TERMINAL_ER_DMPH = X_ASCENDING_FORM_DMPHER/TASCENDING
X_TERMINAL_ER_DMPH = INTEG(D_TERMINAL_ER_DMPH, 0.0)


!Add the absorption rate constants up

RBS_LMPH  =
QDUO*(MEM_DUO_LMPH/(VMEMDUO*KpGut))*1+QJEJ1*(MEM_JEJ1_LMPH/(VMEMJEJ1*KpGut))*1
+QJEJ2*(MEM_JEJ2_LMPH/(VMEMJEJ2*KpGut))*1+QILL1*(MEM_ILL1_LMPH/(VMEMILL1*KpGut
))*1+QILL2*(MEM_ILL2_LMPH/(VMEMILL2*KpGut))*1+QILL3*(MEM_ILL3_LMPH/(VMEMILL3*K
```

```
pGut))*1+
QCECUM*(MEM_CECUM_LMPH/(VMEMCECUM*Kpcolon))*1+QASCENDING*(MEM_ASCENDING_LMPH
/(VMEMASCENDING*Kpcolon))*1
ABS_LMPH  = integ(RBS_LMPH, 0.0)

AMETABOLISM_SI_LMPH =
AMETABOLISM_DUO_LMPH+AMETABOLISM_JEJ1_LMPH+AMETABOLISM_JEJ2_LMPH+AMETABOLISM_I
LL1_LMPH+AMETABOLISM_ILL2_LMPH+AMETABOLISM_ILL3_LMPH+AMETABOLISM_CECUM_LMPH++A
METABOLISM_ASCENDING_LMPH
METABOLISM_SI_LMPH  =
METABOLISM_DUO_LMPH+METABOLISM_JEJ1_LMPH+METABOLISM_JEJ2_LMPH+METABOLISM_ILL1_
LMPH+METABOLISM_ILL2_LMPH+METABOLISM_ILL3_LMPH+METABOLISM_CECUM_LMPH
+METABOLISM_ASCENDING_LMPH


 !Scaled kinetic parameters
    K5d                    = K5dC*BW^0.75               !(1/h)|Enterocyte
metabolism of d-MPH
    K5l                    = K5lC*BW^0.75               !(1/h)|Enterocyte
metabolism of l-MPH
    K5d_cecum              = K5dC_cecum*BW^0.75         !(1/h)|Enterocyte
metabolism of d-MPH
    K5l_cecum              = K5lC_cecum*BW^0.75         !(1/h)|Enterocyte
metabolism of l-MPH

    K5d_ascending          = K5dC_ascending *BW^0.75         !(1/h)|Enterocyte
metabolism of d-MPH
    K5l_ascending          = K5lC_ascending *BW^0.75         !(1/h)|Enterocyte
metabolism of l-MPH


    Vmaxliverd             = VmaxliverdC*BW^0.75  !(ug/h)|Vmax of d-MPH
hydrolysis in the liver
    Vmaxliverl             = VmaxliverlC*BW^0.75  !(ug/h)|Vmax of l-MPH
hydrolysis in the liver
    Ku_RAd                 = Ku_RAdC*BW**0.75     !(L/h) |Urinary excretion of
d-RA
    Ku_RAl                 = Ku_RAlC*BW**0.75     !(L/h) |Urinary excretion of
l-RA
    Kmetd                  = KmetdC*BW**0.75      !(L/h) |Clearance of d-MPH
in the liver via oxidation
    Kmetl                  = KmetlC*BW**0.75      !(L/h) |Clearance of l-MPH
in the liver via oxidation


    !!!!!!!%%%%%%%%%%%%%%%%%%%%%%%%%%%%%%%%%%%%%%   PBPK model for DMPH
%%%%%%%%%%%%%%%%%%%%%%%%%%%%%%%%!!!

    !----Amount of d-MPH in the plasma
    Rplasmad                =  QC*(CVd -CAd )          !(ug/h)|Rate of d-MPH
amount change in the plasma.
    APlasmad                =  INTEG (Rplasmad ,0.0)   !(ug)  |Amout of d-MPH
in the plasma.
    CAd                     =  Aplasmad /Vplasma       !(ug/L)|d-MPH
concentration in the artery plasma.
    CVd                     =  (CVLiverd*QLiver + CVFatd*QFat + CVRd*QR
+CVSd*QS+ CVgonadd*Qgonad+ CVbraind*Qbrain+CVheartd*Qheart+IVRd)/QC
!(ug/L)|d-MPH concentration in the venous plasma.

    !----Amount of d-MPH in the fat
    RAFatd                  = QFat*(CAd -CVfatd  )     !(ug/h)|Rate of d-MPH
amount change in the fat.
    AFatd                   = INTEG(RAFatd  , 0.0)     !(ug)  |Amout of d-MPH
in the fat.
    CVFatd                  = AFatd /(VFat*PFat)       !(ug/L)|venous blood
concentration of d-MPH leaving the fat.
```

```
    CFatd                    = AFatd /VFat              !(ug/L)|d-MPH
concentration in the fat.


    !----Amount of d-MPH in the gonads
    RAgonadd                 = Qgonad*(Cad  -CVgonadd )   !(ug/h)|Rate of d-MPH
amount change in the gonads.
    Agonadd                  = INTEG(RAgonadd  , 0.0)     !(ug)  |Amout of
d-MPH in the goands.
    CVgonadd                 = Agonadd  /(Vgonad*Pgonad)  !(ug/L)|venous blood
concentration of d-MPH leaving the gonads.
    Cgonadd                  = Agonadd /Vgonad            !(ug/L)|d-MPH
concentration in the goands.

    !----Amount of d-MPH in the liver
    RALIVERD                 =
(QLiver-QDUO-QJEJ1-QJEJ2-QILL1-QILL2-QILL3-Qcecum-Qascending)*CAD -
QLIVER*CVLIVERD  -Rmetd -Rmet_liverd  + RBS_DMPH
    ALiverd                  = INTEG(RAliverd ,0.0)      !(ug)  |Amout of d-MPH
in the liver.
    CVLiverd                 = ALiverd /(VLiver*PLiver)  !(ug/L)|venous blood
concentration of d-MPH leaving the liver.
    CLiverd                  = ALiverd/VLiver            !(ug/L)|d-MPH
concentration in the liver.
    Rmetd                    = Kmetd*CVliverd            !(ug/h)|Rate of d-MPH
oxidation in the liver
    Ametd                    = INTEG(Rmetd , 0.0)        !(ug)  |Amount of
d-MPH oxidation in the liver

    Rmet_liverd              =
Vmaxliverd*CVliverd/(Kmliverd*(1+CVliverl/Kmliverl)+CVliverd)     !(ug/h)|
Rate of d-MPH hydrolysis in the liver
    Amet_liverd              = INTEG(Rmet_liverd,0.0)  !(ug)  |Amount of d-MPH
hydrolysis in the liver

    !----Amount of d-MPH in the brain
    Rbraind                 = Qbrain*(CAd-CVbraind)    !(ug/h)|Rate of d-MPH
amount change in the brain.
    Abraind                 = INTEG(Rbraind,0.0)       !(ug)  |Amout of d-MPH
in the brain.
    CVbraind                = Abraind/(Vbrain*Pbrain)  !(ug/L)|venous blood
concentration of d-MPH leaving the brain.
    Cbraind                 = Abraind/Vbrain           !(ug/L)|d-MPH
concentration in the brain.

    !----Amount of d-MPH in rapidly perfused tissues
    RARd                    = QR*(CAd-CVRd)            !(ug/h)|Rate of d-MPH
amount change in the rapidly perfused tissues
    ARd                     = INTEG(RARd,0.0)          !(ug)  |Amout of d-MPH
in the rapidly perfused tissues
    CVRd                    = ARd/(VR*PRich)           !(ug/L)|venous blood
concentration of d-MPH leaving the rapidly perfused tissues
    CRd                     = ARd/VR
!(ug/L)|d-MPH concentration in the rapidly perfused tissues


    !----Amount of d-MPH in slwoly perfused tissues
    RASd                    = QS*(CAd-CVSd)            !(ug/h)|Rate of d-MPH
amount change in the slowly perfused tissues
    ASd                     = INTEG(RASd,0.0)          !(ug)  |Amout of d-MPH
in the slowly perfused tissues
    CVSd                    = ASd/(VS*PSlow)           !(ug/L)|venous blood
concentration of d-MPH leaving the slowly perfused tissues
    CSd                     = ASd/VS                   !(ug/L)|d-MPH
concentration in the slowly perfused tissues
```

```
    !----Amount of d-MPH in the heart
    Rheartd                = QHeart*(CAd-CVheartd)    !(ug/h)|Rate of d-MPH
amount change in the heart
    Aheartd                = INTEG(Rheartd,0.0)       !(ug)  |Amout of d-MPH
in the heart
    CVheartd               = Aheartd/(Vheart*Pheart)  !(ug/L)|venous blood
concentration of d-MPH leaving the heart
    Cheartd                = Aheartd/Vheart           !(ug/L)|d-MPH
concentration in the heart

    !! Model for d-RA
    Rmet_RAd               = Rmet_liverd*(220/233.3062) !(ug/h)|formation rate
of d-RA in the liver
    Amet_RAd               = Integ (Rmet_RAd, 0.0)      !(ug)  |Amount of d-RA
formed in the liver
    RRAurined              = Ku_RAd*CVRAd               !(ug/h)|rate of d-RA
excretion into the urine
    ARAurined              = integ(RRAurined,0.0)       !(ug)  |Amount of d-RA
excretion into the urine
    RxRAd                  = METABOLISM_SI_DMPH*F*(220/233.3062)
!(ug/h)|Formation and uptake rate of d-RA from the gut into the system
    AxRAd                  = Integ (RxRAd, 0.0)            !(ug)  |Amoutn of
RA formed in the gut and taken up into the system
    Rplasmarad             = Rmet_RAd - RRAurined+RxRAd    !(ug/h)|Rate of
d-RA amount change in the system
    APlasmarad             = INTEG(Rplasmarad, 0.0)        !(ug)  |Amount of
d-RA in the system
    CVrad                  = Aplasmarad/Vbody              !(ug/L)|d-RA
concentration in the system


    !!!!!!! %%%%%%%%%%%%%%%%%%%%%% PBPK model for LMPH
%%%%%%%%%%%%%%%%%%%%%%%%%%%%%%%%%%%%%%%%%%%%%%%%%

    !----Amount of l-MPH in the plasma
    Rplasmal               =  QC*(CVl -CAl )            !(ug/h)|Rate of d-MPH
amount change in the plasma.
    APlasmal               =  INTEG (Rplasmal ,0.0)     !(ug)  |Amout of d-MPH
in the plasma.
    CAl                    =  Aplasmal /Vplasma         !(ug/L)|d-MPH
concentration in the artery plasma.
    CVl                    =  (CVLiverl*QLiver + CVFatl*QFat + CVRl*QR
+CVSl*QS+ CVgonadl*Qgonad+ CVbrainl*Qbrain+CVheartl*Qheart+IVRl)/QC
!(ug/L)|d-MPH concentration in the venous plasma.

    !----Amount of l-MPH in the fat
    RAFatl                 = QFat*(CAl  -CVfatl)     !(ug/h)|Rate of d-MPH
amount change in the fat.
    AFatl                  = INTEG(RAFatl  , 0.0)    !(ug)  |Amout of d-MPH in
the fat.
    CVFatl                 = AFatl /(VFat*PFat)      !(ug/L)|venuos blood
concentration of d-MPH leaving the fat.
    CFatl                  = AFatl /VFat             !(ug/L)|d-MPH
concentration in the fat.


    !----Amount of l-MPH in the gonads
    RAgonadl               = Qgonad*(Cal  -CVgonadl)     !(ug/h)|Rate of d-MPH
amount change in the gonads.
    Agonadl                = INTEG(RAgonadl , 0.0)       !(ug)  |Amout of
d-MPH in the goands.
    CVgonadl               = Agonadl  /(Vgonad*Pgonad)   !(ug/L)|venous blood
concentration of d-MPH leaving the gonads.
    Cgonadl                = Agonadl /Vgonad             !(ug/L)|d-MPH
concentration in the goands.
```

```
    !----Amount of l-MPH in the liver
    RALIVERl              = 
(QLiver-QDUO-QJEJ1-QJEJ2-QILL1-QILL2-QILL3-Qcecum-Qascending)*CAL-QLIVER*CVLIV
ERL  -RmetL -Rmet_liverL  + RBS_LMPH
    ALiverL               = INTEG(RAliverL ,0.0)      !(ug)  |Amout of d-MPH 
in the liver.
    CVLiverL              = ALiverL /(VLiver*PLiver)  !(ug/L)|venous blood 
concentration of d-MPH leaving the liver.
    CLiverL               = ALiverL /VLiver           !(ug/L)|d-MPH 
concentration in the liver.
    RmetL                 = Kmetl*CVliverL            !(ug/h)|Rate of d-MPH 
oxidation in the liver
    AmetL                 = INTEG (RmetL , 0.0)       !(ug)  |Amount of d-MPH 
oxidation in the liver

    Rmet_liverL           = 
VmaxliverL*CVliverL/(KmliverL*(1+CVLIVERD/KMLIVERD) +CVliverL)!(ug/h)| Rate of
d-MPH hydrolysis in the liver
    Amet_liverL           = INTEG(Rmet_liverL , 0.0)  !(ug)  |Amount of d-MPH 
hydrolysis in the liver

    !----Amount of l-MPH in the brain
    RbrainL               = Qbrain*(CAL-CVbrainL)    !(ug/h)|Rate of d-MPH 
amount change in the brain.
    AbrainL               = INTEG(RbrainL,0.0)       !(ug)  |Amout of d-MPH 
in the brain.
    CVbrainL              = AbrainL/(Vbrain*Pbrain)  !(ug/L)|venous blood 
concentration of d-MPH leaving the brain.
    CbrainL               = AbrainL/Vbrain           !(ug/L)|d-MPH 
concentration in the brain.

    !----Amount of l-MPH in rapidly perfused tissues
    RARL                  = QR*(CAL-CVRL)            !(ug/h)|Rate of d-MPH 
amount change in the rapidly perfused tissues
    ARL                   = INTEG(RARL,0.0)          !(ug)  |Amout of d-MPH 
in the rapidly perfused tissues
    CVRL                  = ARL/(VR*PRich)           !(ug/L)|venous blood 
concentration of d-MPH leaving the rapidly perfused tissues
    CRL                   = ARL/VR                   !(ug/L)|d-MPH 
concentration in the rapidly perfused tissues


    !----Amount of l-MPH in slwoly perfused tissues
    RASL                  = QS*(CAL-CVSL)            !(ug/h)|Rate of d-MPH 
amount change in the slowly perfused tissues
    ASL                   = INTEG(RASL,0.0)          !(ug)  |Amout of d-MPH 
in the slowly perfused tissues
    CVSL                  = ASL/(VS*PSlow)           !(ug/L)|venous blood 
concentration of d-MPH leaving the slowly perfused tissues
    CSL                   = ASL/VS                   !(ug/L)|d-MPH 
concentration in the slowly perfused tissues

    !----Amount of l-MPH in the heart
    RheartL               = QHeart*(CAL-CVheartL)    !(ug/h)|Rate of d-MPH 
amount change in the heart
    AheartL               = INTEG(RheartL,0.0)       !(ug)  |Amout of d-MPH 
in the heart
    CVheartL              = AheartL/(Vheart*Pheart)  !(ug/L)|venous blood 
concentration of d-MPH leaving the heart
    CheartL               = AheartL/Vheart           !(ug/L)|d-MPH 
concentration in the heart

    !! Model for l-RA
    Rmet_RAL              = Rmet_liverL*(220/233.3062)   !(ug/h)|formation 
rate of d-RA in the liver
    Amet_RAL              = Integ (Rmet_RAL, 0.0)        !(ug)  |Amount of 
```

```
d-RA formed in the liver
    RRAurineL             = Ku_RAL*CVRAL                 !(ug/h)|rate of d-RA
excretion into the urine
    ARAurineL             = integ(RRAurineL,0.0)         !(ug)  |Amount of
d-RA excretion into the urine
    RxRAL                 = METABOLISM_SI_LMPH*F*(220/233.3062)
!(ug/h)|Formation and uptake rate of d-RA from the gut into the system
    AxRAL                 = Integ (RxRAL, 0.0)           !(ug)  |Amoutn of RA
formed in the gut and taken up into the system
    RplasmaraL            = Rmet_RAL - RRAurineL+RxRAL   !(ug/h)|Rate of d-RA
amount change in the system
    APlasmaraL            = INTEG(RplasmaraL, 0.0)       !(ug)  |Amount of
d-RA in the system
    CVraL                 = Aplasmaral/Vbody             !(ug/L)|d-RA
concentration in the system

   !!!!!!!!!!!!!!!!!!!!!!!!!!! %%%%%%%%%%         MASS BALANCE
%%%%%%%%%%%%%%%%%% !!!!!!!!!!!!!!!!!!!!!!!!!!!!!!!!!!!!!!!!!!!!!!!!!
    !!mass balance based on single dose unless otherwise specified

    ctotal     = cvd + cvl              !(ug/L)|Total concentration of d- and
l-MPH
    ctotalra   = cvrad +cvral           !(ug/L)|Total concentration of d- and
l-RA
    amet       = ametd + ametl          !(ug)|Total amount of MPH subject to
oxidation in the liver
    Qtotal     = Qliver+QFat+QR+QS+ Qgonad +Qbrain + Qheart
                  !(L/h)|total blood flow
    Qbal       = QC-Qtotal
                  !(L/h)|blood flow balance
    BWorgans   = Vliver+ vplasma+ VR+ VS + VFat+ Vgonad + Vbrain + Vheart +
VMEMDUO +VMEMJEJ1+VMEMJEJ2+VMEMILL1+VMEMILL2+VMEMILL3+VMEMCECUM+VMEMASCENDING
!(L)|total tissue volumes
    pBW        = BWORGANS/BW

    !!MASS BALANCE FOR THE SYSTEM

    !!DMPH IN THE LUMEN
    LUMENDMPHDISS      = X_STOMACH_DISS_DMPH+X_DUO_DISS_DMPH
+X_JEJ1_DISS_DMPH+X_JEJ2_DISS_DMPH+X_ILL1_DISS_DMPH
+X_ILL2_DISS_DMPH+X_ILL3_DISS_DMPH
+X_CECUM_DISS_DMPH+X_ASCENDING_DISS_DMPH+A_Terminal_DISS_DMPH
    LUMENDMPHIRform    = X_STOMACH_SOLID_DMPHIR+
X_duo_SOLID_DMPHIR+X_JEJ1_SOLID_DMPHIR+X_JEJ2_SOLID_DMPHIR+X_ILL1_SOLID_DMPHIR
+X_ILL2_SOLID_DMPHIR+X_ILL3_SOLID_DMPHIR+X_ASCENDING_SOLID_DMPHIR+X_CECUM_SOLI
D_DMPHIR
    LUMENDMPHERform    = X_STOMACH_FORM_DR_DMPHER+
X_duo_FORM_DMPHER+X_JEJ1_FORM_DMPHER+X_JEJ2_FORM_DMPHER+X_ILL1_FORM_DMPHER+X_I
LL2_FORM_DMPHER+X_ILL3_FORM_DMPHER+X_CECUM_FORM_DMPHER+X_ASCENDING_FORM_DMPHER
    LUMENDMPHFORM      = LUMENDMPHIRform+ LUMENDMPHERform
+A_Terminal_SOLID_dMPH

    !!LMPHIR IN THE LUMEN
    LUMENLMPHDISS      = X_STOMACH_DISS_LMPH+X_DUO_DISS_LMPH
+X_JEJ1_DISS_LMPH+X_JEJ2_DISS_LMPH+X_ILL1_DISS_LMPH
+X_ILL2_DISS_LMPH+X_ILL3_DISS_LMPH
+X_CECUM_DISS_LMPH+X_ASCENDING_DISS_LMPH+A_Terminal_DISS_LMPH
    LUMENLMPHIRform    = X_STOMACH_SOLID_LMPHIR+
X_duo_SOLID_LMPHIR+X_JEJ1_SOLID_LMPHIR+X_JEJ2_SOLID_LMPHIR+X_ILL1_SOLID_LMPHIR
+X_ILL2_SOLID_LMPHIR+X_ILL3_SOLID_LMPHIR+X_ASCENDING_SOLID_LMPHIR+X_CECUM_SOLI
D_LMPHIR
    LUMENLMPHERform    = X_STOMACH_FORM_DR_LMPHER+
X_duo_FORM_LMPHER+X_JEJ1_FORM_LMPHER+X_JEJ2_FORM_LMPHER+X_ILL1_FORM_LMPHER+X_I
LL2_FORM_LMPHER+X_ILL3_FORM_LMPHER+X_ASCENDING_FORM_LMPHER+X_CECUM_FORM_LMPHER
    LUMENLMPHFORM      = LUMENLMPHIRform+ LUMENLMPHERform
+A_Terminal_SOLID_LMPH
```

```
    !!MPH in THE ENTEROCYTES
    CELLDMPH          =
MEM_DUO_DMPH+MEM_JEJ1_DMPH+MEM_JEJ2_DMPH+MEM_ILL1_DMPH
+MEM_ILL2_DMPH+MEM_ILL3_DMPH +MEM_ASCENDING_DMPH+MEM_CECUM_DMPH
    CELLLMPH          =
MEM_DUO_LMPH+MEM_JEJ1_LMPH+MEM_JEJ2_LMPH+MEM_ILL1_LMPH
+MEM_ILL2_LMPH+MEM_ILL3_LMPH +MEM_ASCENDING_LMPH+MEM_CECUM_LMPH

    !!TOTAL MPH IN THE GUT
    GUTDMPH           = LUMENDMPHform+LUMENDMPHDISS+ CELLDMPH
    GUTLMPH           = LUMENLMPHform+LUMENLMPHDISS+ CELLLMPH

    !!MASS FOR MPH MODEL
    MPHBOX            = GUTDMPH + GUTLMPH+ALiverd +ALIVERl+
Aplasmad+Aplasmal+AFatd +aFATl + ASd+ASl + ARd+ARl + Agonadd+AGONADl +
Abraind+ABRAINl  + Aheartd+AHEARTl
    LossMPHLIVER      = AmetL+Ametd+Amet_liverd + Amet_liverl

    LOSSMPGUT         = AMETABOLISM_SI_LMPH+ AMETABOLISM_SI_DMPH
    BALMPH            =
AIVd+AIVl+ADOSEDIR+ADOSEDER+ADOSELIR+ADOSELER-MPHBOX-LOSSMPHLIVER-LOSSMPGUT

    !!ra
    RABOX             = APlasmaraD + APlasmaraL
    RATOMPH           = RABOX *(233.3062/220)
    balra             = Amet_ral +AMET_RAD+AxRAL
+AxRAD-RABOX-ARAurined-ARAurinel
      !(ug)|Mass balance for RA

    !!TOTAL BALANCE
    BAL               = AIVd+AIVl+ADOSEDIR+ADOSEDER+ADOSELIR+ADOSELER
-RATOMPH-MPHBOX-(ARAurined+ARAurinel)*(233.3062/220)-AmetL-AmetD-AMETABOLISM_S
I_DMPH*(1-F)- AMETABOLISM_SI_LMPH*(1-F)
    Ratio_liver       = (AmetL+Ametd)/(AmetL+Ametd+Amet_liverd +
Amet_liverl+1e-34)
    Ratio_total       =
(AmetL+Ametd+AMETABOLISM_SI_DMPH*(1-F)+AMETABOLISM_SI_LMPH*(1-F))/(AmetL+Ametd
+Amet_liverd + Amet_liverl+1e-34+AMETABOLISM_SI_DMPH+AMETABOLISM_SI_LMPH)
    ratio_gut         =
(AMETABOLISM_SI_DMPH*(1-F)+AMETABOLISM_SI_LMPH*(1-F))/(AMETABOLISM_SI_DMPH+AME
TABOLISM_SI_LMPH+1e-34)


    auccv = INTEG(CTOTAL, 0)

    QSC = QS/QC
    QRC = QR/QC
    VSC = VS/BW
    VRC = VR/BW

    END ! DERIVATIVE
    !  Add discrete events here as needed
    !  DISCRETE
    !  END

    ! code that is executed once at each communication interval goes here
    TERMT (T.GE.TSTOP, 'checked on communication interval: REACHED TSTOP')

    END ! DYNAMIC

    TERMINAL
    ! code that is executed once at the end of a simulation run goes here

    END ! TERMINAL
```

```
END ! PROGRAM
```
