# Supplementary material for: Application of Physiologically Based Absorption Modeling to Characterize the Pharmacokinetic Profiles of Oral Extended Release Methylphenidate Products in Adults
Source: PLoS One. 2016 Oct 10;11(10):e0164641. doi: 10.1371/journal.pone.0164641 (PMC5056674; doi:10.1371/journal.pone.0164641)
Supplement: S1 Table — (DOC) [file pone.0164641.s002.doc]

S1 Table. Distribution of parameters used for population analysis.

| **Parameters** | **Mean** | **SD** | **Lower bound** | **Upper bound** | **Distribution** |
| --- | --- | --- | --- | --- | --- |
| **Blood flows (fraction of cardiac output)** |  |  |  |  |  |
| QCC, L/h/kg^0.75^ | 15.87 | 1.4 | 13.1 | 18.7 | Lognormal |
| QFatC(Male) | 0.053 | 0.016 | 0.022 | 0.084 | Normal |
| QFatC(Female) | 0.091 | 0.027 | 0.037492 | 0.144508 | Normal |
| QLiverC (Male) | 0.255 | 0.077 | 0.105 | 0.405 | Normal |
| QLiverC (Female) | 0.27 | 0.081 | 0.11124 | 0.42876 | Normal |
| QBrainC | 0.11 | 0.033 | 0.045 | 0.17 | Normal |
| QGonadC(Male) | 0.00054 | 0.00016 | 0.00022 | 0.00086 | Normal |
| QGonadC(Female) | 0.00022 | 0.00007 | 9.064E-05 | 0.0003494 | Normal |
| QSC(Male) | 0.149 | 0.045 | 0.061388 | 0.236612 | Normal |
| QRC(Female) | 0.39446 | 0.118 | 0.162518 | 0.62640248 | Normal |
| QSC(Male) | 0.102 | 0.031 | 0.042024 | 0.161976 | Normal |
| QRC(Female) | 0.38 | 0.114 | 0.15656 | 0.60344 | Normal |
| QHeartC (Male) | 0.038 | 0.011 | 0.0157 | 0.0603 | Normal |
| QHeartC (Female) | 0.047 | 0.014 | 0.019364 | 0.074636 | Normal |
| QArteryC | 0.065 | 0.020 | 0.02678 | 0.10322 | Normal |
|  |  |  |  |  |  |
| **Tissues volumes (fraction of body weight)** |  |  |  |  |  |
| Body weight(BW, kg) | 83.4 | 25.400 | 33.616 | 133.184 | Lognormal |
| VPlasmaC | 0.0435 | 0.013 | 0.0179 | 0.0691 | Normal |
| VFatC (Male) | 0.213 | 0.064 | 0.088 | 0.34 | Normal |
| VFatC (Female) | 0.327 | 0.098 | 0.134724 | 0.519276 | Normal |
| VLiverC | 0.026 | 0.008 | 0.011 | 0.041 | Normal |
| VBrainC | 0.02 | 0.006 | 0.008 | 0.03 | Normal |
| VHeartC (Male) | 0.0045 | 0.001 | 0.001854 | 0.007146 | Normal |
| VHeartC (Female) | 0.0042 | 0.001 | 0.0017304 | 0.0066696 | Normal |
| VGonadC(Male) | 0.0007 | 0.0002 | 0.0003 | 0.001 | Normal |
| VGonadC(Female) | 0.0027 | 0.001 | 0.0011124 | 0.0042876 | Normal |
| VSC(Male) | 0.3825 | 0.115 | 0.15759 | 0.60741 | Normal |
| VRC(Male) | 0.229685 | 0.069 | 0.09463 | 0.36473978 | Normal |
| VSC(Female) | 0.2688 | 0.081 | 0.1107456 | 0.4268544 | Normal |
| VRC(Female) | 0.227 | 0.068 | 0.093524 | 0.360476 | Normal |
| VMEMDUOC(Male) | 0.0008 | 0.0002 | 0.0003 | 0.001 | Normal |
| VMEMDUOC(Female) | 0.000877 | 0.00026 | 0.0003613 | 0.0013927 | Normal |
| VMEMJEJ1C(Male) | 0.0019 | 0.001 | 0.0008 | 0.003 | Normal |
| VMEMJEJ1C(Female) | 0.00192 | 0.001 | 0.000791 | 0.003049 | Normal |
| VMEMJEJ2C(Male) | 0.0019 | 0.001 | 0.0008 | 0.003 | Normal |
| VMEMJEJ2C(Female) | 0.00192 | 0.001 | 0.000791 | 0.003049 | Normal |
| VMEMILL1C(Male) | 0.0014 | 0.0004 | 0.0006 | 0.0023 | Normal |
| VMEMILL1C(Female) | 0.00149 | 0.00045 | 0.0006139 | 0.0023661 | Normal |
| VMEMILL2C(Male) | 0.0014 | 0.0004 | 0.0006 | 0.0023 | Normal |
| VMEMILL2C(Female) | 0.00149 | 0.00045 | 0.0006139 | 0.0023661 | Normal |
| VMEMILL3C(Male) | 0.0014 | 0.0004 | 0.0006 | 0.0023 | Normal |
| VMEMILL3C(Female) | 0.00149 | 0.00045 | 0.0006139 | 0.0023661 | Normal |
| VMEMCECUMC(Male) | 0.0004 | 0.00012 | 0.00016 | 0.00064 | Normal |
| VMEMCECUMC(Female) | 0.0004 | 0.00012 | 0.0001648 | 0.0006352 | Normal |
| VMEMASCENDINGC(Male) | 0.00085 | 0.00025 | 0.00035 | 0.00135 | Normal |
| VMEMASCENDINGC(Female) | 0.00093 | 0.00028 | 0.0003832 | 0.0014768 | Normal |
|  |  |  |  |  |  |
| **Partition coefficients for MPH** |  |  |  |  |  |
| Pfat | 1.79 | 0.358 | 1.08832 | 2.49168 | Lognormal |
| Pbrain | 6.07 | 1.214 | 3.69056 | 8.44944 | Lognormal |
| Prich | 5.66 | 1.132 | 3.44128 | 7.87872 | Lognormal |
| Pslow | 2.47 | 0.494 | 1.50176 | 3.43824 | Lognormal |
| Pgonads | 3.12 | 0.624 | 1.89696 | 4.34304 | Lognormal |
| Pheart | 2.19 | 0.438 | 1.33152 | 3.04848 | Lognormal |
| Pliver | 5.66 | 1.132 | 3.44128 | 7.87872 | Lognormal |
| Kpgut | 5.66 | 1.132 | 3.44128 | 7.87872 | Lognormal |
| Kpcolon | 5.66 | 1.132 | 3.44128 | 7.87872 | Lognormal |
|  |  |  |  |  |  |
| **Surface area (cm^2^)** |  |  |  |  |  |
| ESA_DUO | 19995 | 1999.5 | 16075.98 | 23914.02 | Lognormal |
| ESA_JEJ1 | 77482 | 7748.2 | 62295.53 | 92668.47 | Lognormal |
| ESA_JEJ2 | 69217 | 6921.7 | 55650.47 | 82783.53 | Lognormal |
| ESA_ILL1 | 60952 | 6095.2 | 49005.41 | 72898.59 | Lognormal |
| ESA_ILL2 | 52171 | 5217.1 | 41945.48 | 62396.52 | Lognormal |
| ESA_ILL3 | 43906 | 4390.6 | 35300.42 | 52511.58 | Lognormal |
| ESA_CECUM | 1964 | 196.4 | 1579.056 | 2348.944 | Lognormal |
| ESA_ASCENDING | 2961 | 296.1 | 2380.644 | 3541.356 | Lognormal |
|  |  |  |  |  |  |
| **Transit time (hr)** |  |  |  |  |  |
| TSTOMACH(fasted) | 0.25 | 0.025 | 0.201 | 0.299 | Lognormal |
| TSTOMACH(fed) | 1 | 0.100 | 0.804 | 1.196 | Lognormal |
| TDUO | 0.26 | 0.026 | 0.20904 | 0.31096 | Lognormal |
| TJEJ1 | 0.95 | 0.095 | 0.7638 | 1.1362 | Lognormal |
| TJEJ2 | 0.76 | 0.076 | 0.61104 | 0.90896 | Lognormal |
| TILL1 | 0.59 | 0.059 | 0.47436 | 0.70564 | Lognormal |
| TILL2 | 0.43 | 0.043 | 0.34572 | 0.51428 | Lognormal |
| TILL3 | 0.31 | 0.031 | 0.24924 | 0.37076 | Lognormal |
| TCECUM | 4.5 | 0.450 | 3.618 | 5.382 | Lognormal |
| TASCENDING | 13.5 | 1.350 | 10.854 | 16.146 | Lognormal |
|  |  |  |  |  |  |
| **PH values** |  |  |  |  |  |
| PHSTOMACH (fasted) | 1.3 | 0.078 | 1.14712 | 1.45288 | Lognormal |
| PHDUO (fasted) | 6 | 0.180 | 5.6472 | 6.3528 | Lognormal |
| PHJEJ1(fasted) | 6.2 | 0.186 | 5.83544 | 6.56456 | Lognormal |
| PHJEJ2(fasted) | 6.4 | 0.192 | 6.02368 | 6.77632 | Lognormal |
| PHSTOMACH (fed) | 4.9 | 0.294 | 4.32376 | 5.47624 | Lognormal |
| PHDUO (fed) | 5.4 | 0.162 | 5.08248 | 5.71752 | Lognormal |
| PHJEJ1(fed) | 5.4 | 0.162 | 5.08248 | 5.71752 | Lognormal |
| PHJEJ2(fed) | 6 | 0.180 | 5.6472 | 6.3528 | Lognormal |
| PHILL1 | 6.6 | 0.198 | 6.21192 | 6.98808 | Lognormal |
| PHILL2 | 6.9 | 0.207 | 6.49428 | 7.30572 | Lognormal |
| PHILL3 | 7.4 | 0.222 | 6.96488 | 7.83512 | Lognormal |
| PHCECUM | 6.4 | 0.192 | 6.02368 | 6.77632 | Lognormal |
| PHASCENDING | 6.8 | 0.204 | 6.40016 | 7.19984 | Lognormal |
|  |  |  |  |  |  |
| **Luminal Volume (mL)** |  |  |  |  |  |
| VSTOMACH | 50 | 5.000 | 40.2 | 59.8 | Lognormal |
| VDUO | 48 | 4.800 | 38.592 | 57.408 | Lognormal |
| VJEJ1 | 175 | 17.500 | 140.7 | 209.3 | Lognormal |
| VJEJ2 | 140 | 14.000 | 112.56 | 167.44 | Lognormal |
| VILL1 | 109 | 10.900 | 87.636 | 130.364 | Lognormal |
| VILL2 | 79 | 7.900 | 63.516 | 94.484 | Lognormal |
| VILL3 | 56 | 5.600 | 45.024 | 66.976 | Lognormal |
| VCECUM | 53 | 5.300 | 42.612 | 63.388 | Lognormal |
| VASCENDING | 57 | 5.700 | 45.828 | 68.172 | Lognormal |
|  |  |  |  |  |  |
| **Human Peff (cm/s)** |  |  |  |  |  |
| HPEFF_EXP | 9.63E-05 | 0.0000193 | 5.86E-05 | 0.00013405 | Lognormal |
| HPEFF_EXP_CECUM | 0.0000963 | 0.0000193 | 5.86E-05 | 0.00013405 | Lognormal |
| HPEFF_EXP_ASCENDING | 0.0000963 | 0.0000193 | 5.86E-05 | 0.00013405 | Lognormal |
|  |  |  |  |  |  |
| **Bile concentration (mM)** |  |  |  |  |  |
| BILE_DUO(fasted) | 2.8 | 0.280 | 2.2512 | 3.3488 | Lognormal |
| BILE_JEJ1(fasted) | 2.33 | 0.233 | 1.87332 | 2.78668 | Lognormal |
| BILE_JEJ2(fasted) | 2.03 | 0.203 | 1.63212 | 2.42788 | Lognormal |
| BILE_ILL1(fasted) | 1.41 | 0.141 | 1.13364 | 1.68636 | Lognormal |
| BILE_ILL2(fasted) | 1.16 | 0.116 | 0.93264 | 1.38736 | Lognormal |
| BILE_ILL3(fasted) | 0.14 | 0.014 | 0.11256 | 0.16744 | Lognormal |
| BILE_DUO(fed) | 14.44 | 1.444 | 11.60976 | 17.27024 | Lognormal |
| BILE_JEJ1(fed) | 12.02 | 1.202 | 9.66408 | 14.37592 | Lognormal |
| BILE_JEJ2(fed) | 10.46 | 1.046 | 8.40984 | 12.51016 | Lognormal |
| BILE_ILL1(fed) | 7.28 | 0.728 | 5.85312 | 8.70688 | Lognormal |
| BILE_ILL2(fed) | 5.99 | 0.599 | 4.81596 | 7.16404 | Lognormal |
| BILE_ILL3(fed) | 0.73 | 0.073 | 0.58692 | 0.87308 | Lognormal |
|  |  |  |  |  |  |
| **Dissolution** |  |  |  |  |  |
| SOL (mg/L) | 9250 | 925.000 | 7437 | 11063 | Lognormal |
| DIFFCOEFF_IR (cm2/min) | 0.0001 | 0.000 | 8.04E-05 | 0.0001196 | Lognormal |
| RHO (g/mL) | 1 | 0.100 | 0.804 | 1.196 | Lognormal |
| RPARTICLE (µm) | 5 | 0.500 | 4.02 | 5.98 | Lognormal |
| DLT (µm) | 30 | 3.000 | 24.12 | 35.88 | Lognormal |
|  |  |  |  |  |  |
| **Metabolic constants** |  |  |  |  |  |
| Kmliverd (µg/L) | 27600 | 8280 | 11371 | 43828 | Lognormal |
| Kmliverl (µg/L) | 10172 | 3051.6 | 4190.864 | 16153.14 | Lognormal |
| VmaxliverdC (µg/h/kg^0.75^) | 25826 | 7747.8 | 10640.31 | 41011.69 | Lognormal |
| VmaxliverlC (µg/h/kg^0.75^) | 52404 | 15721.2 | 21590.45 | 83217.55 | Lognormal |
| KmetdC (L/h/kg^0.75^) | 0.43 | 0.129 | 0.17716 | 0.68284 | Lognormal |
| KmetlC (L/h/kg^0.75^) | 0.43 | 0.129 | 0.17716 | 0.68284 | Lognormal |
| K5lC (1/h/kg^0.75^) | 37.8 | 11.34 | 15.5736 | 60.0264 | Lognormal |
| K5dC (1/h/kg^0.75^) | 0.79 | 0.237 | 0.32548 | 1.25452 | Lognormal |
|  |  |  |  |  |  |
| **Release of ER** |  |  |  |  |  |
| *MLR-MPH* |  |  |  |  |  |
| A (hr^b^) | 6 | 0.600 | 4.824 | 7.176 | Lognormal |
| B | 4 | 0.400 | 3.216 | 4.784 | Lognormal |
| C (hr) | 4 | 0.400 | 3.216 | 4.784 | Lognormal |
| *Ritalin LA, Medikinet Retard,*  *Metadate CD* |  |  |  |  |  |
| A (hr^b^) | 0.5 | 0.050 | 0.402 | 0.598 | Lognormal |
| B | 2 | 0.200 | 1.608 | 2.392 | Lognormal |
| C (hr) | 4 | 0.400 | 3.216 | 4.784 | Lognormal |
