# Supplementary material for: Application of Physiologically Based Absorption Modeling to Characterize the Pharmacokinetic Profiles of Oral Extended Release Methylphenidate Products in Adults
Source: PLoS One. 2016 Oct 10;11(10):e0164641. doi: 10.1371/journal.pone.0164641 (PMC5056674; doi:10.1371/journal.pone.0164641)
Supplement: S2 Table — (DOC) [file pone.0164641.s003.doc]

S2 Table. Model predicted versus observed pharmacokinetic model parameters for subjects receiving IR MPH under fasting conditions.

|  | **T_max_ (hr)^a^** | |  | **C_max_ (ng/mL)^a^** | |  | **AUC_last_ (ng*hr/mL)^a,b^** | | |
| --- | --- | --- | --- | --- | --- | --- | --- | --- | --- |
|  | **Observation** | **Prediction** |  | **Observation** | **Prediction** |  | **Range (hr)** | **Observation** | **Prediction** |
| ***d*-MPH** |  |  |  |  |  |  |  |  |  |
| *Patrick et al. 2013* | 2.4±1.1 | 1.9±0.36 |  | 10.1±3.1 | 9.2±3.2 |  | 0-12 | 47.9±13.9 | 53.2±18 |
| *Patrick et al. 2007* | 2.3±0.75 | 2.1±0.31 |  | 15.3±3.4 | 9.0±3.2 |  | 0-10 | 75.3±19.7^c^ | 48.7±16.4 |
| *Wong et al. 1998* | 1.5 (1.0-3.0) | 2.1 (0.7-3.5) |  | 17.8±3.9 | 15.9±6.1 |  | 0-18 | 92.3±32^c^ | 101.9±38.1 |
| ***l*-MPH** |  |  |  |  |  |  |  |  |  |
| *Patrick et al. 2013* | 1.8±0.88 | 1.76±0.45 |  | 0.18±0.15 | 0.19±0.12 |  | 0-8 | 0.45±0.54 | 0.76±0.43 |
| *Patrick et al. 2007* | NA | NA |  | NA | NA |  | NA | NA | NA |
| *Wong et al. 1998* | 0.5 (0.5-1.0) | 1.8 (0.4-3.1) |  | 0.82±0.56^d^ | 0.31±0.2 |  | 0-2 | 1.34±0.91^c,d^ | 0.45±0.31 |

a, Values are presented as mean ± SD, except for T_max_ for the study of Wong et al. (1998), which is expressed as the median (range);

b, AUC_last_, area under the curve from time 0 to the last measurable time point which vary among different studies;

c, AUC_last_ values for the studies of Patrick et al. (2007) and Wong et al. (1998) were derived using the equation of AUC_last_ = AUC_0-∞_ - C_last_/k, where AUC_0-∞_ is the reported area under the curve from time 0 to infinity, C_last_ is the last measurable concentration, and k is the elimination rate constant;

d, Recalculated C_max_ and AUC_last_ are 0.23±0.41 ng/mL and 0.45 ng*hr/mL for *l*-MPH based on digitalized data from Figure 2 of Wong et al. (1998).
