# Supplementary material for: Application of Physiologically Based Absorption Modeling to Characterize the Pharmacokinetic Profiles of Oral Extended Release Methylphenidate Products in Adults
Source: PLoS One. 2016 Oct 10;11(10):e0164641. doi: 10.1371/journal.pone.0164641 (PMC5056674; doi:10.1371/journal.pone.0164641)
Supplement: S3 Table — (DOC) [file pone.0164641.s004.doc]

**S3 Table. Model predicted versus observed pharmacokinetic model parameters for subjects receiving MLR MPH under fasting conditions.**

|  | **T_max_ (hr)^a^** | | **C_max_ (ng/mL)^a^** | | **T_max1_ (hr, 0-4)^a^** | | **C_max1_ (ng/mL)^a^** | | **T_max2_ (hr, 4-12)^a^** | | **C_max2_(ng/mL)^a^** | |
| --- | --- | --- | --- | --- | --- | --- | --- | --- | --- | --- | --- | --- |
| **Reference** | ***Obs.*** | ***Pred.*** | ***Obs.*** | ***Pred.*** | ***Obs.*** | ***Pred.*** | ***Obs.*** | ***Pred.*** | ***Obs.*** | ***Pred.*** | ***Obs.*** | ***Pred.*** |
| *Reiz 2008* | 3.71±2.03 | 2.9±2 | 5.07±1.32 | 4.0±2.8 | 2.26±0.64 | 2.1±0.32 | 4.80±1.39 | 3.9±2.8 | 6.02±1.26 | 6.1±1.1 | 4.73±1.09 | 3.3±2.3 |
| *Adjei 2014* | 2.0 | 2.1 | 23.5±11.4 | 13.9±5.3 | NA | NA | NA | NA | NA | NA | NA | NA |

|  | **AUC_last_ (ng*hr/mL)^a,b^** | | | **AUC_1_(ng*hr/mL)^a,b^** | | | **AUC_2_ (ng*hr/mL)^a,b^** | | |
| --- | --- | --- | --- | --- | --- | --- | --- | --- | --- |
| **Reference** | ***Range***  ***(hr)*** | ***Obs.*** | ***Pred.*** | ***Range (hr)*** | ***Obs.*** | ***Pred.*** | ***Range (hr)*** | ***Obs.*** | ***Pred.*** |
| *Reiz 2008* | 0-24 | 52.32±12.67 | 47.6±32.7 | 0-4 | 12.35±3.78 | 11.8±8.5 | 4-12 | 28.57±6.68 | 22±15.3 |
| *Adjei 2014* | 0-24 | 262.7±134.8 | 174.1±61 | 0-4 | 60.1±27.7 | 40.5±16 | NA | NA | NA |

a, Values are presented as mean ± SD, except for T_max_ for the study of Adjei et al. (2014), which is expressed as the median;

b, AUC, area under the curve from time 0 to different time points which vary among different studies.
