# Supplementary material for: Application of Physiologically Based Absorption Modeling to Characterize the Pharmacokinetic Profiles of Oral Extended Release Methylphenidate Products in Adults
Source: PLoS One. 2016 Oct 10;11(10):e0164641. doi: 10.1371/journal.pone.0164641 (PMC5056674; doi:10.1371/journal.pone.0164641)
Supplement: S4 Table — (DOC) [file pone.0164641.s005.doc]

**S4 Table. Model predicted versus observed pharmacokinetic model parameters for subjects receiving Ritalin-LA under fasting conditions.**

|  | **T_max_ (hr)^a^** | | **C_max_ (ng/mL)^a^** | | **T_max1_ (hr, 0-4)^a^** | | **C_max1_ (ng/mL)^a^** | | **T_max2_**  **(hr)^a^** | | **C_max2_(ng/mL)^a^** | |
| --- | --- | --- | --- | --- | --- | --- | --- | --- | --- | --- | --- | --- |
| **Reference** | ***Obs.*** | ***Pred.*** | ***Obs.*** | ***Pred.*** | ***Obs.*** | ***Pred.*** | ***Obs.*** | ***Pred.*** | ***Obs.*** | ***Pred.*** | ***Obs.*** | ***Pred.*** |
| *Lee 2003* | 4.22±2.02 | 2.6±1.2 | 15.23±2.45 | 8.6±3.4 | 1.56±0.74 | 2.1±0.48 | 12.47±2.60 | 8.5±3.4 | 5.44±0.62^b^ | 4.9±0.49^b^ | 14.84±2.71^b^ | 7.3±2.9^b^ |
| *Markowitz 2003* | 5.5±0.825 | 2.6±1.2 | 9.9±4.1 | 4.7±1.7 | 2.1±1.0 | 2.1±0.47 | 7.0±3.29 | 4.7±1.9 | NA | NA | NA | NA |
| *Haessler 2008* | NA | NA | 14.5±3.02 | 7.9±2.9 | NA | NA | 10.0±3.51 | 7.8±2.9 | NA | NA | 14.5±3.02^c^ | 6.7±2.5^c^ |

|  | **AUC_last_ (ng*hr/mL)^a,d^** | | | **AUC_1_(ng*hr/mL)^a,d^** | | | **AUC_2_(ng*hr/mL)^a,d^** | | |
| --- | --- | --- | --- | --- | --- | --- | --- | --- | --- |
| **Reference** | ***Range***  ***(hr)*** | ***Obs.*** | ***Pred.*** | ***Range (hr)*** | ***Obs.*** | ***Pred.*** | ***Range (hr)*** | ***Obs.*** | ***Pred.*** |
| *Lee 2003* | 0-24 | 103.32±19.42 | 80.1±32.5 | 0-4 | 31.16±6.94 | 25.4±10.2 | 4-8 | 40.11±7.15 | 25.0±9.6 |
| *Markowitz 2003* | 0-24 | 75.0±40.5 | 48.3±18.2 | 0-4 | 18.5±8.14 | 14.0±5.8 | NA | NA | NA |
| *Haessler 2008* | 0-24 | 111.7±30.3 | 81±27.2 | 0-4 | 27.6±8.89 | 23.3±8.7 | 4-10 | 57.1±14.36 | 31.7±10.9 |

a, Values are presented as mean ± SD;

b, 4-8 hr;

c, 4-10 hr;

d, AUC, area under the curve from time 0 to different time points which vary among different studies.
