# Supplementary material for: Application of Physiologically Based Absorption Modeling to Characterize the Pharmacokinetic Profiles of Oral Extended Release Methylphenidate Products in Adults
Source: PLoS One. 2016 Oct 10;11(10):e0164641. doi: 10.1371/journal.pone.0164641 (PMC5056674; doi:10.1371/journal.pone.0164641)
Supplement: S5 Table — (DOC) [file pone.0164641.s006.doc]

**S5 Table. Model predicted versus observed pharmacokinetic model parameters for subjects receiving Metadate CD under fasting conditions.**

|  | **T_max_ (hr)^a^** | | **C_max_ (ng/mL)^a^** | | **T_max1_ (hr, 0-4)^a^** | | **C_max1_ (ng/mL)^a^** | | **T_max2_**  **(hr, 4-10)^a^** | | **C_max2_(ng/mL)^a^** | |
| --- | --- | --- | --- | --- | --- | --- | --- | --- | --- | --- | --- | --- |
| **Reference** | ***Obs.*** | ***Pred.*** | ***Obs.*** | ***Pred.*** | ***Obs.*** | ***Pred.*** | ***Obs.*** | ***Pred.*** | ***Obs.*** | ***Pred.*** | ***Obs.*** | ***Pred.*** |
| *Pentikis 2002* | 4.58±1.17 | 5.0±1.3 | 4.58±1.97 | 3.3±1.2 | NA | NA | NA | NA | NA | NA | NA | NA |
| *Gonzalez 2002* | NA | NA | NA | NA | NA | NA | 3.33±0.94 | 2.9±1.2 | NA | NA | 3.89±0.99 | 3.5±1.4 |

|  | **AUC_last_ (ng*hr/mL)^a,b^** | | | **AUC_1_(ng*hr/mL)^a,b^** | | | **AUC_2_(ng*hr/mL)^a,b^** | | | **AUC_3_(ng*hr/mL)^a,b^** | | |
| --- | --- | --- | --- | --- | --- | --- | --- | --- | --- | --- | --- | --- |
| **Reference** | ***Range***  ***(hr)*** | ***Obs.*** | ***Pred.*** | ***Range (hr)*** | ***Obs.*** | ***Pred.*** | ***Range (hr)*** | ***Obs.*** | ***Pred.*** | ***Range (hr)*** | ***Obs.*** | ***Pred.*** |
| *Pentikis 2002* | 0-24 | 41.71±18.80 | 40.2±13.3 | NA | NA | NA | NA | NA | NA | NA | NA | NA |
| *Gonzalez 2002* | 0-24 | 36.31±10.92 | 43±15.5 | 0-4 | 10.01±3.06 | 8.3±3.4 | 0-6 | 17.11±4.65 | 14.4±5.7 | 0-8 | 22.78±6.01 | 20.5±7.8 |

a, Values are presented as mean ± SD

b, AUC, area under the curve from time 0 to different time points which vary among different studies.
