# Supplementary material for: Application of Physiologically Based Absorption Modeling to Characterize the Pharmacokinetic Profiles of Oral Extended Release Methylphenidate Products in Adults
Source: PLoS One. 2016 Oct 10;11(10):e0164641. doi: 10.1371/journal.pone.0164641 (PMC5056674; doi:10.1371/journal.pone.0164641)
Supplement: S6 Table — (DOC) [file pone.0164641.s007.doc]

S6 Table. Model predicted versus observed pharmacokinetic model parameters for subjects receiving Medikinet Retard under fed conditions.

|  | **T_max_ (hr)^a^** | | **C_max_ (ng/mL)^a^** | | **T_max1_ (hr, 0-4)^a^** | | **C_max1_ (ng/mL)^a^** | | **T_max2_**  **(hr, 4-10)^a^** | | **C_max2_(ng/mL)^a^** | |
| --- | --- | --- | --- | --- | --- | --- | --- | --- | --- | --- | --- | --- |
| **Reference** | ***Obs.*** | ***Pred.*** | ***Obs.*** | ***Pred.*** | ***Obs.*** | ***Pred.*** | ***Obs.*** | ***Pred.*** | ***Obs.*** | ***Pred.*** | ***Obs.*** | ***Pred.*** |
| *Haessler 2008* | NA | NA | 19.6±5.95 | 8.3±3 | NA | NA | 16.5±6.69 | 6.6±2.5 | NA | NA | 18.3±5.66 | 8.3±3.0 |
| *Schutz 2009* | 4.06±1.65 | 5±0.4 | 5.26±2.11^b^ | 5±1.5^b^ | 2.82±1.00 | 3.7±0.27 | 4.83±1.87^b^ | 3.9±1.5^b^ | 5.33±0.84 | 5.0±0.38 | 5.02±2.04^b^ | 5.0±1.4^b^ |
| *Fischer 2006* | 2.02^c^ | 5^c^ | 4.52±0.88^b^ | 4.5±1.5^b^ |  |  |  |  |  |  |  |  |

|  | **AUC_last_ (ng*hr/mL)^a,d^** | | | **AUC_1_(ng*hr/mL)^a,d^** | | | **AUC_2_(ng*hr/mL)^a,d^** | | | |
| --- | --- | --- | --- | --- | --- | --- | --- | --- | --- | --- |
| **Reference** | ***Range***  ***(hr)*** | ***Obs.*** | ***Pred.*** | ***Range (hr)*** | ***Obs.*** | ***Pred.*** | ***Range (hr)*** | ***Obs.*** | ***Pred.*** | |
| *Haessler 2008* | 0-24 | 138.7±37.4 | 80.9±27.5 | 0-4 | 42.4±16.54 | 14.7±5.6 | 4-10 | 68.0±19.47 | | 38.5±13.3 |
| *Schutz 2009* | 0-24 | 39.16±13.83^b^ | 49.1±1.4^b^ | 0-4 | 11.72±4.64^b^ | 8.7±1.5^b^ | 4-24 | 26.99±10.07^b^ | 40.2±1.4^b^ | |
| *Fischer 2006* | 0-16 | 35.05±6.75^b^ | 44.5±1.4^b^ |  |  |  |  |  |  | |

a,Values are presented as mean ± SD, except for b, values are expressed as geometric mean ± SD; and c, values are expressed as median;

d, AUC, area under the curve from time 0 to different time points whch vary among different studies.
